# Identified of a novel *cis*-element regulating the alternative splicing of *LcDREB2*

**DOI:** 10.1038/srep46106

**Published:** 2017-04-06

**Authors:** Zhujiang Liu, Guangxiao Yuan, Shu Liu, Junting Jia, Liqin Cheng, Dongmei Qi, Shihua Shen, Xianjun Peng, Gongshe Liu

**Affiliations:** 1 Key Laboratory of Plant Resources, Institute of Botany, the Chinese Academy of Sciences, Beijing, 100093, People’s Republic of China; 2 University of the Chinese Academy of Sciences, Beijing, 100049, People’s Republic of China

## Abstract

Alternative splicing (AS) is an important gene regulation mechanism in plants. Despite the widespread use of AS in plant gene expression regulation, the identification of the *cis*-elements involved in the AS mechanism is rarely reported in plants. To explore the regulation mechanism of the AS of *LcDREB2*, a *DREB2* ortholog from Sheepgrass (*Leymus chinensis*), the genomic sequences of *LcDREB2* and its homologs in Poaceae were aligned, and six mutations were introduced in the conserved sequence of *LcDREB2*. By analyzing the distinct transcript patterns of the *LcDREB2* mutants in transgenic *Oryza sativa*, a novel *cis*-element that affected the AS of *LcDREB2* was identified as Exonic Splicing Enhancer 1 (ESE1). In addition, five serine-arginine rich (SR) proteins were confirmed to interact with ESE1 by electrophoretic mobility shift assay (EMSA). To further explore the expression regulation mechanism of the DREB subfamily, phylogenetic analysis of *DREB2* paralogous genes was performed. The results strongly supported the hypothesis that AS is conserved in Poaceae plants and that it is an evolutionary strategy for the regulation of the functional expression of genes. The findings and methods of our study will promote a substantial step forward in understanding of the plant AS regulation mechanism.

Alternative splicing (AS) of pre-mRNA generates diversity in the transcriptome and proteome of eukaryotic organisms and plays an important role in gene regulation and tissue-specific expression^1^,^2^. The basis of splicing is the recognition of introns and exons by the splicing machinery^3^. The key component in the regulation of this process is the spliceosome, which is composed of several proteins and recognizes the splice site^4^. As a fundamental molecular process, AS is tightly regulated by *cis*-elements within exons and surrounding introns as well as *trans*-acting factors that bind to these *cis*-elements^5^.

The RNA elements involved in AS include exonic and intronic splicing enhancers (ESEs and ISEs) or silencers (ESSs and ISSs)^6^. The *cis*-elements are reported to have conserved sequences. The splicing factor SRSF1 binds exon 11 at evolutionarily conserved sites and blocks the splicing of MDM2^7^. The *cis*-elements could be bound by RNA-binding proteins that regulate AS in the organism. Even a single base mutation of a *cis*-element can cause aberrant splicing in a number of diseases^8^. Though the research on *cis*-elements has been mostly focused on their RNA sequences, the secondary structure also plays an important role in regulating AS. MBNL1 binds a portion of one intron as a stem-loop, whereas U2AF65 binds the same region in a single-strand structure, and they appear to compete by binding to mutually exclusive RNA structures to regulate AS^9^.

The most widely studied *trans*-acting factors regulating AS are proteins of the SR (Ser/Arg-rich) and hnRNP (heterogeneous nuclear ribonucleoprotein) families, as well as numerous tissue-specified AS factors^10^,^11^,^12^. These factors generally regulate AS by recognizing *cis*-elements in exons or introns and by promoting or suppressing the assembly of the spliceosome at adjacent splice sites. SR proteins are RNA-binding proteins that are generally thought to activate splicing by binding to *cis*-elements on exons and recruiting components of the spliceosome^13^. There has been considerable information reported regarding SR proteins. The SR protein-specific kinase was shown to be activated by Akt to phosphorylate SR proteins downstream, and the signals were transduced by the Akt-SRPK-SR axis to regulate AS in the nucleus^14^. The SR protein homolog TgSR3 was shown to affect the AS of over 10,000 genes in *Toxoplasma gondii*^15^. In addition, the high frequency of AS among the SR family of proteins in plants has been linked to important roles in gene regulation during development and in response to environmental stress^16^.

High-throughput DNA sequencing has promoted the advances of AS study. The conserved AS events in flowering plants have been identified by genome-wide analysis. Of the expressed multi-exonic genes, 70.4% undergo AS events in *Amborella trichopoda*. AS events in other taxa are also observed, 64.4% of the expressed multi-exonic genes occur AS in *Vitis vinifera*, 53.2% in *Populus trichocarpa*, 50.2% in *Glycine max*, 46.4% in *Oryza sativa*, 44.9% in *Phaseolus vulgaris*, 44.7% in *Medicago truncatula* and 39.1% in *Solanum lycopersicum*^17^. The most recent data based on whole transcriptome sequencing, which allows *de novo* detection of unknown transcript variants, indicate that 61% of all *Arabidopsis* genes are alternatively spliced^18^,^19^. For comparison, in mammals, approximately 95% of all multi-exon genes undergo alternative splicing^4^. Alternative pre-mRNA splicing occurs from 95% to 100% of human genes and approximately 63% of mouse genes^20^,^21^.

The importance of AS in humans has been dramatically highlighted, due to numerous diseases are closely related to AS^22^,^23^. Similarly, AS in plants has been found during abiotic, biotic stress and development^24^. The importance of AS in plants has been increasingly recognized in the last decade. In *A. thaliana*, the mutation of nuclear speckle RNA-binding protein (NSR) changed the AS pattern of genes and significantly reduced the number and length of lateral roots^25^. Heat stress induced AS of miR-400 caused a lower germination percentage and less hypocotyl elongation in *A. thaliana*^26^.

The alternative splicing mechanism has been well studied in humans and other animals, but there was almost no studies have focused on AS mechanism in plants, particularly on the identification of *cis*-elements. In the plant SR protein gene *SCL33*, an intronic splicing regulatory element involved in AS has been identified^27^. In the third intron of the *A. thaliana* proline synthesis enzyme *P5CS1 (Δ1-pyrroline-5-carboxylate synthetase 1*), a 150-nt sequence has been reported to promote AS and given rise to the production of a nonfunctional transcript^28^.

The DREB (Dehydration-Responsive Element-Binding) transcription factors, a subfamily of the AP2/ERF superfamily that contain a single AP2/ERF domain^29^, are mainly involved in abiotic stress responses^30^,^31^,^32^. *LcDREB2* is a transcription factor that was isolated from *Leymus chinensis*, which is an important perennial forage grass with strong resistance to salt and drought stress^33^. Our previous research has showed that *LcDREB2* produced three transcripts, of which *LcDREB2*a and *LcDREB2*c could regulate downstream genes to resist abiotic stress, while *LcDREB2b* was supposed to have no function^33^. Under normal conditions, *LcDREB2b* was the most abundant transcript, while the amount of *LcDREB2*a and *LcDREB2*c transcripts significantly increased under abiotic stress.

The expression of a gene family for the purpose of increasing its function can be regulated by gene duplication or AS^34^,^35^. The single gene *LcDREB2* employs AS for its increased expression. The orthologous genes of *LcDREB2* in *O. sativa*^36^*, Zea mays*^37^, *Hordeum vulgare*^38^, and wheat^39^ also showed a same AS phenomenon. In contrast, none of the *DREB* genes identified in *A. thaliana* exhibited AS^40^,^41^. Apparently, AS is not the only regulation mechanism of the expression of DREB subfamily genes. An analysis of AS and gene duplication in the DREB subfamily to reveal the phylogenetic relationships of expression mechanisms of the DREB subfamily has not been performed. Moreover, the AS regulation mechanisms of *DREB* genes in plants are also unknown. Thus, to understand the AS mechanism of the *LcDREB2* pre-mRNA, and in particular to reveal the *cis*-elements participating in AS process, we mutated the conserved region of *LcDREB2*′s genomic sequence to identify the *cis*-elements that are essential for *LcDREB2* AS in regulating the expression of three transcripts under abiotic stress. To understand why the *DREB2* pre-mRNA is differentially spliced in *O. sativa* and *Arabidopsis*, which are representative plants of the Poaceae and Brassicaceae families, respectively, we performed phylogenetic analysis to explore the regulation mechanisms of AS and gene duplication in the DREB subfamily from different plant species.

## Results

### AS pattern of *LcDREB2*

**Figure 1:**
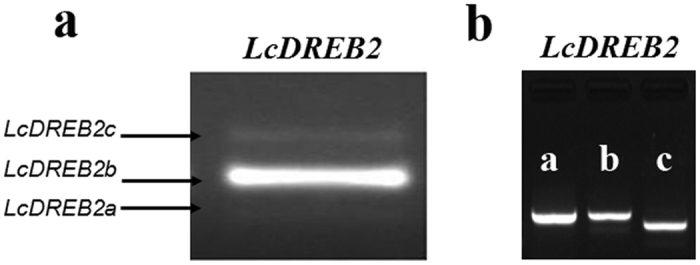
Alternative splicing patterns of the *DREB2* gene in *L. chinensis*. **(a)** AS pattern of *LcDREB2* in *L. chinensis. LcDREB2a, LcDREB2b* and *LcDREB2c* were amplified simultaneously. **(b)** The specific fragment of three AS transcripts of *LcDREB2,* including *LcDREB2a, LcDREB2b* and *LcDREB2c*, which were amplified separately.

The *DREB2* gene of *L. chinensis* produces three transcripts: *LcDREB2a, LcDREB2b* and *LcDREB2c* (Fig. 1a). The *LcDREB2b* transcript, which is 1463 bp in length, was the major AS product. In contrast, *LcDREB2a* and *LcDREB2c*, which are 1410 bp and 1551 bp in length, respectively, are much less abundant. To confirm the expression of the three transcripts, the transcripts were separately amplified by their own primers, which just amplified the specific fragment of their own transcripts. Additionally, the separate bands of each transcript were shown in Fig. 1b.

### Highly conserved sequences were identified on exon 3

**Figure 2:**
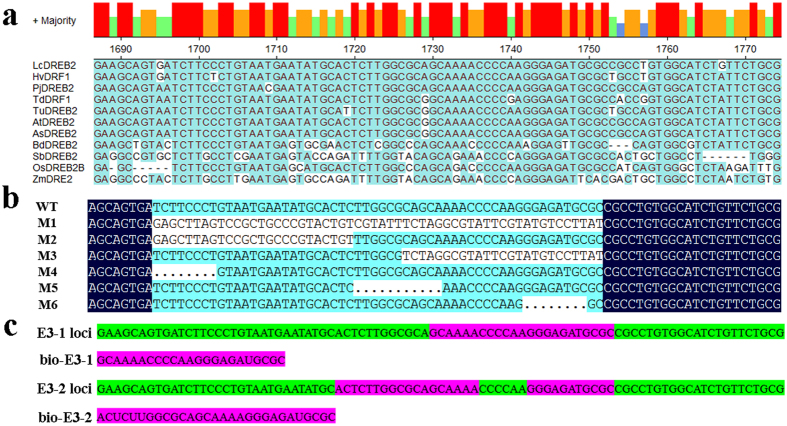
Conserved sequences and mutations of *LcDREB2* exon 3 and RNA probes designed based on the exon 3 sequence. **(a)** The genomic sequences of *LcDREB2, HvDRF1, PjDREB2, TdDRF1, TuDREB2, AtDREB2, AsDREB2, BdDREB2, SbDREB2, OsDREB2B* and *ZmDREB2* were aligned. The red pillars represent highly conserved sequences, and the green pillars represent sequences that are not conserved. The sequences on exon 3 were highly conserved. **(b)** Mutations were carried out on the conserved sequences of exon 3. All the mutations and the wild-type sequence were aligned by DNAMAN 5.2. The location and sequences are shown in the alignment pattern. **(c)** The RNA probes E3-1 and E3-2 were located on exon 3 of *LcDREB2*. The purple-highlighted sequences reflect the RNA probe locations, and the sequences are listed below with RNA bases.

To isolate the site of *cis*-elements that are critical for the AS of *LcDREB2*, we aligned the genomic sequences of *LcDREB2* and its orthologous genes from 10 other gramineous plants (see Supplementary Fig. S1). Because the second and third exons are different between the three transcripts of *LcDREB2*, and a highly conserved motif on exon 3 was screened through sequence alignment (Fig. 2a), six mutations (from M1 to M6) were designed and introduced into the conserved sequence of exon 3 (Fig. 2b). Nearly the entire sequence of exon 3 was mutated in the M1 mutation. The 5′ and 3′ ends of exon 3 were mutated in the M2 and M3 mutations, respectively. The other three mutations were deletions that were evenly distributed in exon 3 (Fig. 2b). Two RNA probes, E3-1 and E3-2, were designed on the alignment of the exon 3 sequence. The location and corresponding sequences of these probes on exon 3 were shown in Fig. 2c.

### A novel *cis*-element ESE1 was confirmed by mutation experiments

**Figure 3:**
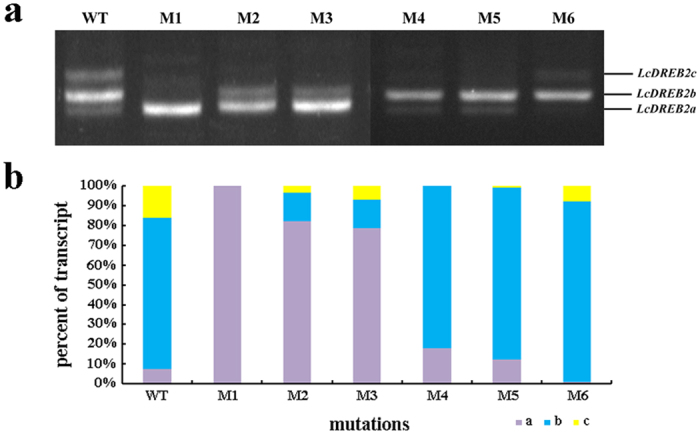
Semi-quantitative and real-time PCR results of *LcDREB2* mutants in transgenic *O. sativa.* **(a)** The transcript patterns of the wild-type exon 3 and all 6 mutants in transgenic *O. sativa* were analyzed by reverse transcription PCR and amplification. The M1, M2 and M3 mutants altered the transcript pattern compared with the wild type, resulting in decreased expression of the major transcript *LcDREB2b* and increased expression of *LcDREB2a*. The M4, M5 and M6 mutants had no effect on the AS pattern of *LcDREB2*. **(b)** The quantity of the three transcripts in transgenic *O. sativa* were detected by real-time PCR. The relative quantity of three transcripts in the wild type and six mutants were compared. The purple, blue and yellow represent *LcDREB2a, LcDREB2b* and *LcDREB2c*, respectively.

Semi-quantitative PCR was used to characterize the effects of these six mutants. Compared with the wild-type, the AS pattern of *LcDREB2* with the mutations was altered. The M1, M2 and M3 mutations led to a change in the ratio of the three transcripts: *LcDREB2a* became the major product among the three transcripts, while the expression of *LcDREB2b* was sharply decreased (Fig. 3a). The M4, M5 and M6 mutations had no obvious changes (Fig. 3a). To further characterize the results and quantify the transcripts of the six different mutants, real-time quantitative PCR was performed. The results in Fig. 3b showed that the M1 mutant only produced *LcDREB2a*, while the other two transcripts were barely detected. The M2 and M3 mutants produced all three transcripts, but *LcDREB2a* was increased substantially, while the primary major transcript of *LcDREB2b* was decreased. Therefore, according to these mutation results, a novel *cis*-element was confirmed in exon 3 of *LcDREB2*. According to the effect of this *cis*-element, it is an exonic splicing enhancer (ESE) and was named as ESE1.

### SR proteins involved in *LcDREB2* AS were screened by an EMSA experiment

**Figure 4:**
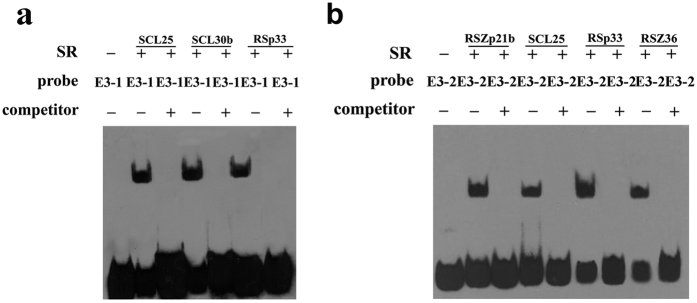
Identification of SR proteins binding to RNA probes by EMSA. EMSA experiments were carried out to screen SR proteins that specifically bound to the RNA probes. The shifted bands indicate probes that were bound by proteins. A competition assay was performed to verify the specific binding of the RNA probes and SR proteins. A 100-fold excess of non-labeled RNA probes was used for the competition. **(a)** The E3-1 RNA probe was specifically bound by the three SR proteins SCL25, SCL30b and RSp33. **(b)** The E3-2 RNA probe was specifically bound by the three SR proteins RSZp21b, SCL25, RSp33 and RSZ36.

SR proteins have been reported to be important AS regulators in previous studies, therefore, we investigated the interaction between SR proteins and the *cis*-element ESE1. In this study, we designed two RNA probes mimicking the novel *cis*-element to screen the SR proteins that may interact with the *cis*-element. The EMSA results showed that three SR proteins, SCL25, SCL30b and RSp33, specifically bound to the E3-1 probe (Fig. 4a) and that four SR proteins, RSZp21b, SCL25, RSp33 and RSZ36, specifically bound to the E3-2 probe (Fig. 4b). Finally, five of 20 SR proteins were screened out from *O. sativa*, namely, Zp21b, SCL25, SCL30b, RSp33 and RSZ36. These proteins showed a positive interaction with the two RNA probes by EMSA (Fig. 4). Among these five SR proteins, two belong to the RS2Z subfamily. The others belong to the 9G8, SC35 type and RS subfamilies, respectively. The other *O. sativa* SR proteins did not show interaction with the probes.

### The secondary structure of the *cis*-element

**Figure 5:**
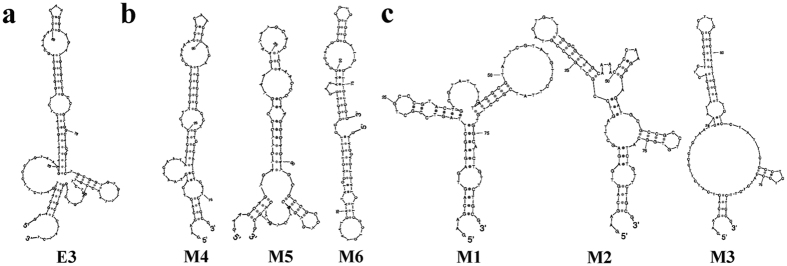
Secondary structure of wild-type and mutated exon 3. **(a)** The secondary structure of wild-type exon 3 for comparison. **(b)** The secondary structures of the M4, M5 and M6 mutations of exon 3: no apparent changes in secondary structure were observed. **(c)** The secondary structures of the M1, M2 and M3 mutations of exon 3: significant changes were observed in these secondary structures.

To identify whether the primary sequence or the secondary structure of the *cis*-element on exon 3 was involved in AS regulation, we analyzed the secondary structure of exon 3 and its mutated sequences by energy minimization modeling using the program Sfold. The exon 3 of wild-type *LcDREB2* had several pseudoknots in the middle sequences of exon 3, and the other sequences formed RNA-RNA pairs. The integral structure was a long strip, and there was a small knot-like protuberance. Both ends of exon 3 were located together at one end of the structure (Fig. 5a). Meanwhile, the secondary structure of exon 3 in M4, M5 and M6 mutants had no obvious changes compared with the wild type (Fig. 5b). They all formed a long strip with several small pseudoknots. However, the secondary structure of exon 3 in M1, M2 and M3 mutants did show obviously changes (Fig. 5c). M1 contained a large pseudoknot, and the integral structure presented a “Y” shape. M2 had more pseudoknots and two small knot-like protuberances, which changed the integral structure. M3 had a large and obvious pseudoknot compared with the wild type.

### Phylogenetic analysis of the DREB subfamily

**Figure 6:**
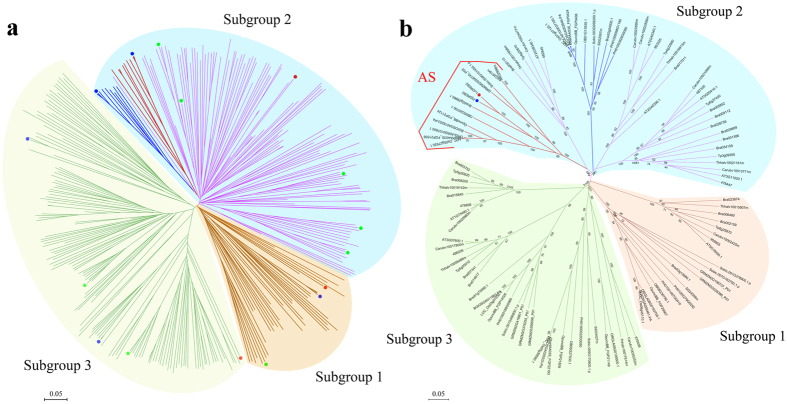
Phylogenetic tree of the A-2 group of the DREB subfamily. **(a)** The phylogenetic tree of the A-2 group of the DREB subfamily proteins of 53 species. The phylogenetic tree is divided into three subgroups. The green, blue and red points denote genes of *A. thaliana*, rice and *Amborella trichopoda*, respectively. The gray branch in subgroup 2 is the AS branch, and the blue branch in subgroup 2 is the one-intron member branch. **(b)** The phylogenetic tree of the A-2 group of the DREB subfamily proteins in Poaceae and Brassicaceae. The A-2 group of Poaceae and Brassicaceae is divided into three subgroups. In subgroup 2, the red branch is the AS branch, and the blue branch is the one-intron branch. The red point represents *LcDREB2*, and the blue point represents *PjDREB2*, which are both located in the AS branch of subgroup 2.

**Figure 7:**
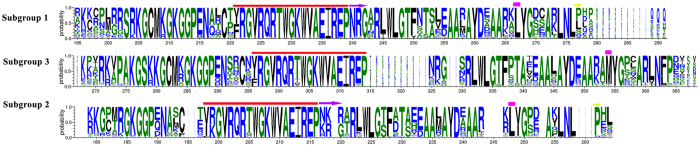
Conserved sequences of three subgroups of the DREBs A-2 group. The conserved AP2 domains are shown in logos. The different signs over the sequences represent obvious differences among the three subgroups. Near the C-terminus, subgroup 3 had a conserved MYG motif, while subgroup 1 and subgroup 2 had an LYG motif. Subgroup 1 had a conserved NRG motif after the common, conserved EIREP, which was not conserved in subgroup 2.

To phylogenetically analyze the expression regulation of the DREB subfamily, all A-2 group DREB proteins from 53 selected species (see Supplementary Table S1) were aligned for phylogenetic analysis. The sequence alignment and phylogenetic analysis results suggested that this group can be further separated into three subgroups, subgroups 1 to 3 (Fig. 6a). In *A. trichopoda*, a basal angiosperm in phylogenetics, we found one member in each subgroup of the A-2 group DREBs. This classification was also supported by the conserved hallmark site of AP2 domains (Fig. 7). The *DREB* genes with AS were only clustered in subgroup 2, and *LcDREB2* belongs to this subgroup.

To further analyze A-2 group DREB proteins in Poaceae and Brassicaceae family, we performed sequence alignment and phylogenetic analysis for this group of DREBs from these two families. Consistent with the phylogenetic tree constructed from the A-2 group DREB of 53 species, the phylogenetic tree of the A-2 group DREBs from Poaceae and Brassicaceae also suggested that the A-2 group DREBs can be classified into three subgroups (Fig. 6b). For all species in these two families, there was one member in subgroup 1 and two members in subgroup 3, except for those species which had undergone genome-wide duplication in their evolutionary history. In subgroup 2, there apparently exhibited diverse in Poaceae and Brassicaceae DREBs. The DREBs with AS clustered into subgroup 2, and all the clustered AS genes belong to Poaceae plants. In addition to the AS clade, the Poaceae plants have another clade with genes possessing one intron and without AS. The Brassicaceae plants possessing gene duplication had three to eight DREB protein clusters in this subgroup (Fig. 6b). The two evolutionary strategies of AS and gene duplication in regulating gene expression therefore co-existed in the DREB subfamily.

## Discussion

Posttranscriptional control of gene expression by regulated pre-mRNA splicing has been attracting more attention, as AS in plants has been found to reshape the transcriptome in particular in response to biotic and abiotic stress^42^,^43^. The *DREB* gene has been studied for years because of its important function in stress resistance^31^,^44^. However, no studies have reported regarding the AS of *DREB*, especially the *cis*-elements regulating AS. Our previous study has showed that the *LcDREB2* gene exhibited an AS phenomenon^33^. It’s homologous genes in other gramineous plants, including *OsDREB2B*^36^, *ZmDREB2A*^37^, *HvDRF1*^38^ and *WDREB2*^39^, also showed a similar AS pattern. *LcDREB2* produces three transcripts, among which *LcDREB2b* has been supposed to have no function, while *LcDREB2a* and *LcDREB2c* have been confirmed to function in salt and drought stress resistance^33^. The elements affecting the AS of *LcDREB2* to produce the three transcripts and regulate their composition ratio have not been explored.

A conserved gene sequence was the characteristic of *cis*-elements identified from previous research^7^,^45^. Compared with previous research in plants, demonstrating, for example, that the *cis*-elements in *SCL33* and *P5CS1* were intronic elements^27^,^28^, the ESE1identified in our study was an exonic element. So, this is a novel kind of *cis*-element on exon 3 of *LcDREB2* in plants. The M1 mutant changed the AS pattern of *LcDREB2* so thoroughly that only *LcDREB2a* was expressed. The M2 and M3 mutants also changed the AS pattern, apparently reducing the amount of *LcDREB2b* and increasing the amount of *LcDREB2a*. Considering the sequences and the location of the mutations, the M1 mutation almost covered M2 and M3. The M2 mutation was located on the 5′ end of M1 and the M3 mutation was located on the 3′ end of M2. The sequences mutated in M2 and M3 corresponded to the sequence of M1. The effect of the M2 and M3 mutations was similar to that of M1, but the effect of the M1 mutation was stronger. The three deletion mutations, M4, M5 and M6, had no effect on the AS pattern compared with the base substitution mutations of M1, M2 and M3. The EMSA results indicated that the E3-2 RNA probe specifically bound to three SR proteins might explain this phenomenon. The E3-2 RNA probe contained the sequence on exon 3 with part of the sequence deleted (Fig. 2c). However, the E3-2 RNA probe could still specifically bind SR proteins (Fig. 4b). The above results implied that the deletion mutations on exon 3 still could be recognized by RNA-binding proteins. Therefore, the EMSA results verified the conclusion by that the deletion mutation on exon 3 had no effect on AS regulation. The *cis*-element identified from *SCL33* was also sequence conservative and specifically bound by SR proteins^27^.

In previous research, the position and sequences of *cis*-elements were closely analyzed^46^,^47^. The secondary structure of *cis*-elements also has an effect on AS^26^. The analysis of the secondary structure of exon 3 and its mutated sequences showed that the M1, M2 and M3 mutations exhibited obvious changes compared with the wild-type sequence (Fig. 5c), while the M4, M5 and M6 mutations had no obvious changes (Fig. 5b). The results of the sequence mutations indicated that M1, M2 and M3 affected the AS patterns of *LcDREB2*, while M4, M5 and M6 had no effect. All the mutations that affected the AS pattern of *LcDREB2* also resulted in changes to the secondary structure of exon 3. The M4, M5 and M6 mutations had no effect on the AS pattern of *LcDREB2* possibly because that there was no obvious change in the secondary structure of exon 3. Thus, we inferred that the sequence of the *cis*-element on exon 3 that regulated the AS of *LcDREB2* was coordinated with its secondary structure.

The expression pattern of the single gene *LcDREB2* is regulated by a *cis*-element, while the expression pattern of the whole DREB subfamily is determined by both AS and gene duplication. Gene duplication and AS have been thought to be two distinct evolutionary mechanisms that increase the expression diversity of the genome^48^.

*LcDREB2* was clustered into the A-2 group of the DREB subfamily by phylogenetic analysis in our previous study^33^. Therefore, we selected A-2 group genes of 53 species to perform phylogenetic analysis, and the AS genes clustered into one clade. The orthologous genes of *LcDREB2* in rice and *Arabidopsis* showed different expression patterns^40^,^41^,^49^, so we chose the two families of Poaceae and Brassicaceae for further analysis. The same clustering situation existed in Poaceae and Brassicaceae (Fig. 6). The phylogenetic analysis of the A-2 subgroup DREBs in Poaceae and Brassicaceae showed that genes with AS were clustered into one clade and that *DREB* genes with one intron and without AS in Poaceae were clustered into another clade. The Brassicaceae genes did not exhibit AS but had more than one duplication. Therefore, we proposed that the *DREB2* genes in Brassicaceae with no intron achieved to increase gene expression though the gene duplication, while the *DREB2* genes in Poaceae have evolved to use AS to produce more transcripts and therefore increase gene expression. The phylogenetic analysis indicated that as Poaceae genes evolved from having no intron to containing at least one intron, and when three introns appeared, the AS emerged. In addition, conservation still existed among these genes with AS, as indicated by the fact that they were clustered into one clade by the phylogenetic analysis. In addition, it is very surprisingly that both AS and gene duplication co-exist in the DREB subfamily. The relationship between AS and gene duplication could be explained by the theory that AS isoforms represent differential functional roles of the duplicated genes^48^,^50^. In addition, gene duplication results in the formation of paralogous gene families^34^. The co-existence of two strategies of gene expression regulation in one gene family has rarely been reported.

The percent of genes with AS in plants and the complexity of AS have been demonstrated to be higher than previously predicted^51^,^52^. Important genes in many key aspects of plant development exhibit AS^27^,^53^,^54^. Meanwhile, one gene can produce several transcripts with different functions. For example, of the transcripts produced from Zinc-Induced Facilitator-Like 1 (ZIFL1), the ZIFL1.1 transporter regulates various root auxin-related processes, while the ZIFL1.3 isoform mediates drought tolerance by regulating stomatal closure^55^. Therefore, the AS regulation mechanism should be identified to facilitate its use in potential stress resistance applications.

In conclusion, a novel *cis*-element has been identified to regulate the AS of *LcDREB2* in our study. Both the primary sequence and secondary structure of this novel *cis*-element play important function in the AS process of the *LcDREB2* gene. An alignment of orthologous genes of the DREB2 subfamily indicated the conservation of the *cis*-element sequence. Phylogenetic analysis also revealed that *DREB2* genes in different plant families have evolved to either gene duplication or AS to increase the diversity of transcripts. The hypothesis that corresponding sequences on homologous genes could also affect AS should be identified in other plant species. This element could be potentially targeted to alter the ratio of the three *LcDREB2* transcripts to increase resistance to abiotic stress through an artificial pathway. A systematic investigation of plant AS mechanisms will help us to understand how plant AS events are regulated and what the function of AS is in plant development and stress responses. In addition, the strategies we used could be a substantial step forward in the exploration of the plant AS mechanism.

## Methods

### *LcDREB2* cloning and amplification

Genomic DNA was extracted from 8-week-old *L. chinensis* (Zhongke No. 1) seedlings using a Plant Genomic DNA Rapid Extraction Kit (Bioteke Corporation, China). The primers used for amplification were designed according to the genomic sequences of *LcDREB2*. The separate primers of the three transcripts were designed according to their specific splicing sites. The primers were listed in Supplementary Table S2.

### Alignment of *LcDREB2* and its orthologous genes in Poaceae

The sequences of the orthologous Poaceae genes of *LcDREB2* (JF754585), specifically *HvDRF1* (AY223807), *PjDREB2* (JF766085), *TdDRF1* (JN571427), *TuDREB2* (KF731664), *AtDREB2* (KF731663), *AsDREB2* (KF731665), *BdDREB2* (Bradi2g29960), *SbDREB2* (JF915838), *OsDREB2B* (JF915842) and *ZmDREB2* (JF915834), were downloaded from the NCBI database (http://www.ncbi.nlm.nih.gov/) and the plant transcription factor database (http://planttfdb.cbi.pku.edu.cn/). These 11 *DREB2* sequences were aligned using the MegAlign software from DNAstar (version 7.10).

### Mutation design and construction

The *LcDREB2* mutations, including deletions and base substitution mutations, were designed based on the conserved motif located in exon 3. The mutations included 3 base substitutions (from M1 to M3) and 3 deletions (from M4 to M6), and they were all located on exon 3. These 6 mutations were created using recombination PCR technology. All mutations and the wild-type *LcDREB2* gene were individually cloned into the pUG1301 vector and expressed under the ubiquitous promoter.

### *O. sativa* transformation

All the 7 recombinant plasmids, including the 6 mutations and one wild type, were introduced into agrobacterium EHA105. A total of 7 agrobacterium strains harboring the recombinant pUG1301 plasmids were introduced into the callus of *O. sativa* plants. The method was referred to a previous report^56^. Positive transgenic plants were selected by hygromycin antibiotics (50 ug·mL^−1^).

### Detection of *DREB2* gene expression in transgenic *O. sativa*

Total RNA from all positive transgenic plant material was extracted using a TRIzol Kit (Invitrogen, USA) according to the manufacturer’s protocol and digested by RNase-free DNase I (TaKaRa, Japan) to remove the genomic DNA. After total RNA was quantified using a NanoDrop 2000 instrument (Thermo Fisher, USA), reverse transcription was performed using a PrimeScript^TM^ RT Reagent Kit (TaKaRa, Japan). The expression level of each transcript was evaluated by semi-quantitative PCR. The AS efficiency of these mutations was measured by real-time PCR. Real-time PCR was conducted on a LightCycler^®^ 480 Real-Time PCR System (Roche, Germany) using a SYBR PrimeScript^TM^ PCR Kit (TaKaRa, Japan). The reaction conditions were as follows: 95 °C for 10 s and 40 cycles of 95 °C for 5 s and 60 °C for 20 s. The primers are listed in Supplementary Table S2. The RNA of wild-type *O. sativa* was extracted to ensure that the homologous gene in *O. sativa* could not be amplified by primers to *LcDREB2*. All *DREB2* transcripts were verified by sequencing.

### Expression and purification of SR proteins

Twenty SR proteins from *O. sativa* were selected for the screening of specific RNA-binding proteins. Individual primers for these 20 SR proteins were designed according to their cDNA sequences. The primers were listed in Supplementary Table S2. The cDNA was amplified by PCR and cloned into the PET30a vector for protein expression. Protein expression was induced by IPTG (0.6 mM) for six hours at either 28 °C or 37 °C. To maintain the activity of the proteins, *Escherichia coli* cells expressing the SR proteins were lysed by lysozymes and purified on Ni columns (Qiagen, Germany). The purified SR proteins were then used for EMSA.

### RNA probes and binding to SR proteins by EMSA

Based on the effects of the mutants, two RNA probes, E3-1 and E3-2, were designed based on the exon 3 sequence. The probes were synthesized in Invitrogen Technologies Company (USA). The sequences of the probes were listed in Supplementary Table S2. To identify specific interactions between SR proteins and the RNA probes, total of 20 *O. sativa* SR proteins were expressed and purified from *E. coli* for the EMSA experiments. The EMSA experiments were conducted by using a LightShift Chemiluminescent EMSA Kit (Pierce, 20158, USA) following the manufacturer’s instructions. The binding reactions were performed with each SR protein and each RNA probe. The SR proteins that shifted the RNA probes were further verified by a competition reaction with a 100-fold excess of unlabeled RNA probe.

### Detection of RNA secondary structure

To analyze the secondary structure of exon 3 and its mutated sequences, we submitted the sequence of wild-type exon 3 and all six mutated sequences of exon 3 to the program Sfold (http://sfold.wadsworth.org/cgi-bin/srna.pl). The secondary structure was predicted by energy minimization modeling using the Sfold program.

### Phylogenetic analysis

To analyze the expression regulation of the DREB subfamily phylogenetically, all A-2 group DREB proteins from 53 selected species were aligned using MEGA 7.0^57^ for phylogenetic analysis (see Supplementary Table S1). Additionally, 111 DREB A-2 group proteins belonging to twelve Poaceae and six Brassicaceae plants were separately aligned to construct a phylogenetic tree. The DREB protein sequences of the 53 plant species were downloaded from the PlantTFDB database (http://planttfdb.cbi.pku.edu.cn/). Based on the alignment results, the sequence logos of the conserved AP2 domains were drawn on the website (http://weblogo.berkeley.edu/logo.cgi) to confirm the reliability of our classification. The group containing AS genes was divided into subgroups according to the distribution of *A. trichopoda* which represents the basal angiosperms. The protein sequences were aligned by MUSCLE in MEGA 7.0 and manually adjusted. The phylogenetic trees were reconstructed based on the multiple sequence alignment using the NJ method (neighbor-joining) in MEGA 7.0 with 1,000 bootstrap replicates.

## Additional Information

**Accession codes:** AY223807, JF766085, JN571427, KF731664, KF731663, KF731665, JF915838, JF915842, JF915834, JF754585 (NCBI database http://www.ncbi.nlm.nih.gov/). Bradi2g29960 (the plant transcription factor database http://planttfdb.cbi.pku.edu.cn/).

**How to cite this article:** Liu, Z. *et al*. Identified of a novel *cis*-element regulating the alternative splicing of *LcDREB2. Sci. Rep.* **7**, 46106; doi: 10.1038/srep46106 (2017).

**Publisher's note:** Springer Nature remains neutral with regard to jurisdictional claims in published maps and institutional affiliations.

## Supplementary Material

Supplementary Dataset 1
