# Supplementary material for: Identified of a novel cis-element regulating the alternative splicing of LcDREB2
Source: Sci Rep. 2017 Apr 6;7:46106. doi: 10.1038/srep46106 (PMC5382683; doi:10.1038/srep46106)
Supplement: Supplementary Dataset 1 [file srep46106-s1.doc]

Identified of a novel *cis*-element regulating the alternative splicing of *LcDREB2*

Zhujiang Liu^1,2^, Guangxiao Yuan^1,2^, Shu Liu^1,2^, Junting Jia^1,2^, Liqin Cheng^1^, Dongmei Qi^1^, Shihua Shen^1^, Xianjun Peng^1＊^, Gongshe Liu^1＊^

**Supplementary fig. S1 The alignment of the genomic sequence of *LcDREB2* with the homologous genes in Poaceae**


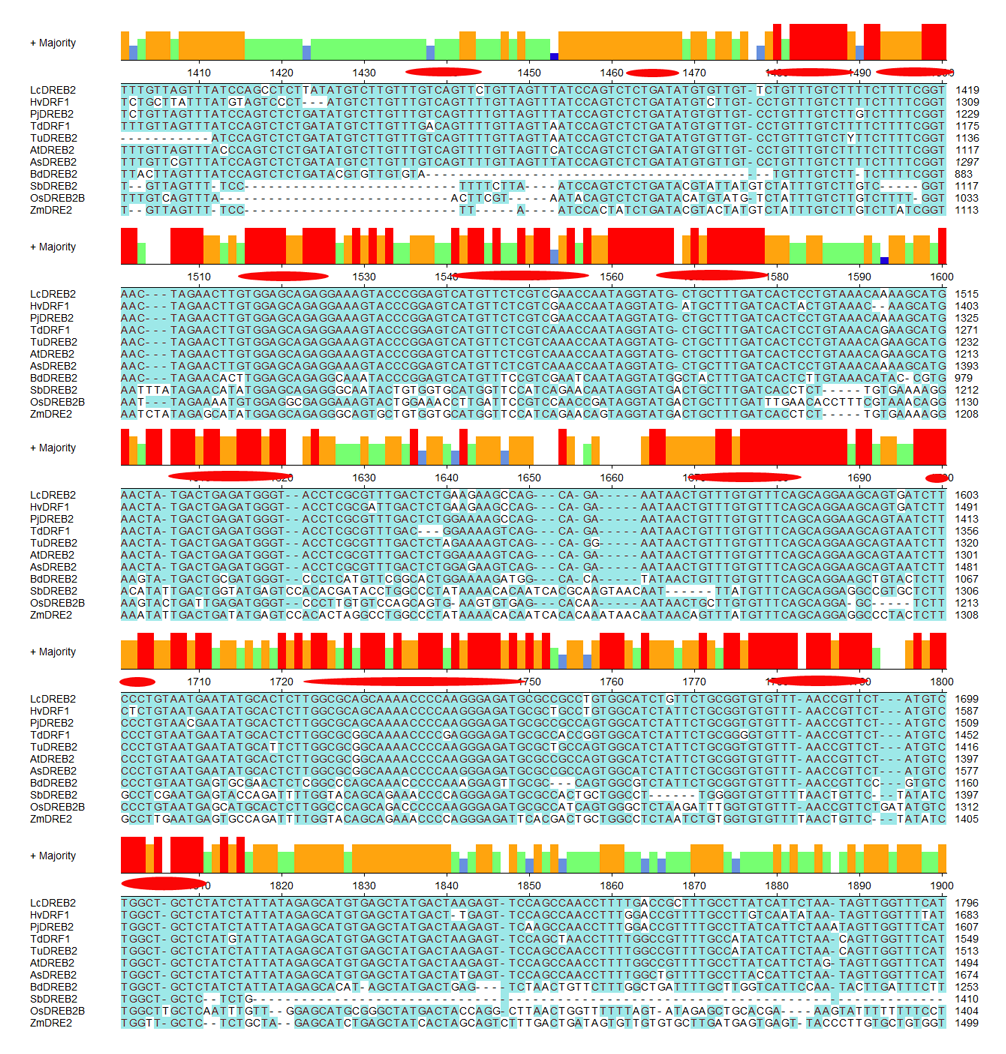


*LcDREB2* and its orthologs from 10 species were aligned by DNAStar 7.10. The bars under the alignment sequences represent the degree of conservation from green to red. The sequences of green bars showed the least conservative and the red bars showed the most conservative. Red ovals over the sequences represent conserved motifs.

**Supplementary Table S1** **The DREB sequences of the A-2 group of the DREB2 subfamily in selected species for phylogenetic analysis**

>evm 27.model.AmTr v1.0 scaffold00022.382 Amborella trichopoda MESCRKNLMKTCKKGPTRGKGGPQNSSCEYRGVRQRTWGKWVAEIREPKKRTRLWLGSFSTAEEAALAYDEAARRLYGPEAHLNLPHIHSNPHSLQKTLSVNWFSSKNFGSNYHSYGVLNLNAQPNVHVIHQRLQEMKKNVNQPPPVSCPLVFSEKGKVNSGPEIEREEKDGLENYGFSAQEKQIDLKEFLEQLGVLKDEQLGVLKEESGSDSSATPPQQILLGNNFPAISETGELDGQDFLWDELEELNGFDHNTDDQTCTIQIEGHEDICLTSSIWDL

>evm 27.model.AmTr v1.0 scaffold00023.14 Amborella trichopoda MLHRRRKTRSRRHGCNSVAETLARWKALNSHPEGSKDAPKWVRKVPAKGSKKGCMRGKGGPENSRCNYRGVRQRTWGKWVAEIREPNRGNRLWLGTFPTALAAALAYDEAARAMYGSCARLNLPDTTESSLTSSISHSNSAEVSSHQIHLDQHSDRGKTGIEGDFDNRGSHALSNTAEVSSHHNATHSSNPAEVSSHHDATHPYPSLDGDKIKIKSEYENISFACRPISSHHTCLEPSDGGKIGIEREFENMGFKRDEIVSNGPGVKLEAAEENLHQRPEVFQVDDMFDVQELLGMMDEEEAGSPSGAHVERDCLASPSALSFQLNNPDAKLLGSLYHMDQLGTPYHMEQGPALDYLGMQDYGFSKAFEEQAEDMNFPDLCF

>evm 27.model.AmTr v1.0 scaffold00039.49 Amborella trichopoda MAMRSSRGSKKGCMKGKGGPENALCAYRGVRQRTWGKWVAEIREPNKGDRLWLGTFATSEEAALAYDTASRKLYGTSARLNLPEFSPFAPKSLISSGGGGNENANVSLASVSNRNLTTSKSQSEQGALEAKASAAFSEIQNSCLLQHCSSPVKNFEPYDDENLFCEDSILDVDVDADLCPVFKIYCDEENQRQELLGLQDLECERISDVVHEISAPDFAADNGGMEAGEEWMNWQEGCDPSHWSWCS

>Aquca 004 00452.1 Aquilegia coerulea MCMSYERKKRSRSGGDVAETLAKWKELHNMNNGSKVINKVPAKGSKKGCMRGKGGPENVNCNYRGVRQRTWGKWVAEIREPNKGKRLWLGTFDTAIEAAKAYDEAAWTMYGTCARLNLPEFIGLNSSSSADLVESSESMESKEFISCPFIKKSEDLKNYIVDAKTENGEVEQIVKVEPKCGQVINCMPNSIDETVMTKDYVKREYPEEVDSLQSNNVAGFGTEQDNSQDIMIDIEELLGQIGYYNDSPPKDFVAMMQNSTDMDSCQPGSTCSDEPQSRPQPSELRDQLQNPAWNVEQANPDYESVWNVEQASRINSYNFLKQNSAQLDYLI

>Aquca 009 00282.1 Aquilegia coerulea MENCRRTPYKPWKKGPTRGKGGPQNASCEYRGVRQRTWGKWVAEIREPKKRTRLWLGSFSTAEEAAMAYDEAARRLYGPDAYLNLPHLHSNFNPLNKSQKLKWFPSKNFISMFPSCSLLNLNGQHNVHVIHQRLQELKRNDTLKQSSMASSSTHDSNYELQIADTKRNSVSLANNVSVANPSAQKQSENFTEKPQLDLTEFLQKLGILKEDSVSDDSNTSASFQVPDFALENNGPVASNEPTFNWDALMEMHGLDHHPSAQATNFPLDDLHEASYSASIWNF

>Aquca 013 00679.1 Aquilegia coerulea MSSDFPERKTNSRSRRNGPNSIAETLAKWKEFNNELESTKDGAKPRKVPAKGSKKGCMRGKGGPDNSRCNYRGVRQRTWGKWVAEIREPNRGRRLWLGTFTTAVDAALAYDAAARAMYGSCAQLNLPDVVREYEMANGSFSAASPSTSESTVTTSNQSGVY

>Aquca 030 00214.1 Aquilegia coerulea MSSDFPERKRKSRSRRNGPNSIAETLAKWKVLNNELESTKDGAKRRKVPAKGSKKGCMRGKGGPDNSRCNYRGVRQRTWGKWVAEIREPNRGSRLWLGTFPTAVDAARAYDAAARAMYGSCARLNLPGVVREYDMSKGSFSAETPSTSESTMTTSNHSGVYGGPEECSVKVECPKMETEHCNAESNETQSQPAPTMYAVKKEVAEENVDADNFNPFKGYNQEYLQDVSVDDMFDVEQLLRTIESEPKNVPVPSEEYNFDSVQYGFSSGGQFQEGGLSNLPYETHKSDANVVEGLYHMDEAAAPAGMDYGYDFLNDSWSPDLGV

>476698 Arabidopsis lyrata MVGANKKQRTVQASSRKGCMRGKGGPDNASCTYKGVRQRTWGKWVAEIREPNRGARLWLGTFDTSREAALAYDSAARKLYGPEAHLNLPESLRSYPETASSQASHTTPSSNTGGKSSDSESPCSSNEMSSCGRVTDEISWEHINVDLPVMDDSSIWEEATMSLGFPWVHEGDNNISRFDTCISGGFSNWDSFHSPL

>478447 Arabidopsis lyrata MAVYEKTGTDTPKKRKSRARADGSTVADRLKKWKEYNEILEVSAIKEGEKPKRKVPAKGSKKGCMKGKGGPDNSHCSFRGVRQRIWGKWVAEIREPNRGQRLWLGTFPTAEEAAAAYDEAASVMYGPLARLNFPQSVGSELTSTSSQSEVCTVENKAVLCGDVCVKHEDIDCESKPCSQILDVREESCVTRPDSCAVGHHDTNSVLNYDLLLEFEQQYWSETLQEKEKPKQEEEEIQQKKRRYSNINSNISNRICLLLQIMILWDPNELFDIDELLGDMNGGMLPGPDQSQDPNHVNSGSYDLHPLHLEPHDGHEFDGLSTLDL

>482845 Arabidopsis lyrata MEKEDNGSKQSSSASVVSSRRRRRRVVEPVEATLQRWEEEGLDKARRVQAKGSKKGCMRGKGGPENPVCRFRGVRQRVWGKWVAEIREPVSHRGANSSRSKRLWLGTFATAAEAALAYDRAASVMYGPYARLNFPEDLGGEMKKDEEAETSGGYWLETEKSGHGVIETKDRKDYVVYNEDAIELGHDKTENPINENPSVKSEENYSFERFKLENGLLYNEPQGSSYHQGGGFDSYLEFFRF

>486209 Arabidopsis lyrata MEKSSSMKQWKKGPARGKGGPQNALCQYRGVRQRTWGKWVAEIREPKKRARLWLGSFATAEEAAMAYDEAALKLYGHDAYLNLPHLQRNTRPSLTNSQRFKWVPSRKFISMFPSCGMLNVNAQPSVHVIQQRLEELKKTGLLSQSYSSSSSSTESKTNTSFLDEKTSKGETDNMFEGADQKKPEIDLTEFLQQLGILKDENQAEPSEVAECHSPPPWNEQEETGSPFKAQNFSWDTLIEMPRTETTTMQFDSSNFRSYDFEDDVSFPSIWDYCGSLD

>487335 Arabidopsis lyrata MAVYDQSGDINRTQLDTSRKRKSRSRADGTTVAERLKRWKEYNENVEEISTKKRKVPAKGSKKGCMKGKGGPENGRCSFRGVRQRIWGKWVAEIREPNRGSRLWLGTFPTAEEAASAYDEAAKAMYGPLARLNFPRSDGSEVTSTSSQSEVCTVETPGCVHVKTEDADCESKPMFCEAKPMYRLENGAEEMKRAVKADKDWLSEFEHNYWRDVLKEKEKQKEQEIVETCQQQEQQDSLSVTDYGWPNDLDQSHLDSSDMFDVDELLRDINGDDVFTGLNQDQYPGNNVTNGSYRLESQQGGFDPLQSLNYGYGLPPFQFEAKDGNGVFDDLSYLDLEN

>870458 Arabidopsis lyrata MDPLASQQHHHHHLDDTDQTLTHNNPQSDSTTDSSTSSAQRKRKGKGGPDNSKFRYRGVRQRSWGKWVAEIREPRKRTRKWLGTFATAEDAARAYDRAAVYLYGSRAQLNLTPSSPSSVSSSSSSVSATSSPSTSSSSTQTLRPLLPRPAAATVGGGATFGPYGIPFNNNIFLNGGTSMLCPSYGLLPHQQQQQQNQMVQVGQFQHQQYQNLHFSTNNNKIGEMELTDVPVVNSTSFHHEVALGQEQGGSGEFVKEITVPIKYIYVEESRRRWWKVVWEFYWFNPSFTVYLI

>903325 Arabidopsis lyrata MGVLEHVANLASMPFDSPRKRKSRGTRDVAEILRQWREYNEQTEADSCIDGGVPKPVRNPPPKGSRKGCMKGKGGPENGICDYRGVRQRTWGKWVAEIREPGRGARLWLGTFSSSYEAALAYDEAAKAIYGQSARLNLPEITNRSSSTAATVSGSVTAFSDESEVCAREDTNERSGFGQVKLEDCNDEYVLLDSSQCIKEELKVKEEVMEEHNSAVGFGIGQDPKREILDAWLMGNGNEQEPLEFGVDETFDINELLGILDDNNVSGQETMQNQVDRQPNFSYQTQFQDANLLGSLNPMEIAHPGVDYGYPYVQPSEMENNGIDLDHHRFNDLDIQDLDFGGEKDVHGST

>909855 Arabidopsis lyrata MEEEQPPAKKRNMGRSRKGCMKGKGGPENATCTFRGVRQRTWGKWVAEIREPNRGTRLWLGTFNTSVEAAMAYDEAAKKLYGHEAKLNLVHPQSLRQQQQQVVVNRNLSFSSHGSGSWASKLDTVPVLDLGLGPASGSRGSWSGRSNFLQEDDDHNYNRRPSSSGSNLCWLLPKQSDSQDQETVNAASGCGVEGGGGSTLTFSTKLKPKNLVVSPNNGLYSGAWSRFLVGQEKKTEHDVSSSCGSSDNKESMAVPSGGGERMHRPEVEERTGYLEMDDLLEIDDLGLLIGKNGDFKNWCCEEFQHPWNWF

>AT1G75490.1 Arabidopsis thaliana MSSIEPKVMMVGANKKQRTVQASSRKGCMRGKGGPDNASCTYKGVRQRTWGKWVAEIREPNRGARLWLGTFDTSREAALAYDSAARKLYGPEAHLNLPESLRSYPKTASSPASQTTPSSNTGGKSSSDSESPCSSNEMSSCGRVTEEISWEHINVDLPVMDDSSIWEEATMSLGFPWVHEGDNDISRFDTCISGGYSNWDSFHSPL

>AT2G38340.1 Arabidopsis thaliana MEKEDNGSKQSSSASVVSSRRRRRVVEPVEATLQRWEEEGLARARRVQAKGSKKGCMRGKGGPENPVCRFRGVRQRVWGKWVAEIREPVSHRGANSSRSKRLWLGTFATAAEAALAYDRAASVMYGPYARLNFPEDLGGGRKKDEEAESSGGYWLETNKAGNGVIETEGGKDYVVYNEDAIELGHDKTQNPMTDNEIVNPAVKSEEGYSYDRFKLDNGLLYNEPQSSSYHQGGGFDSYFEYFRF

>AT2G40340.1 Arabidopsis thaliana MPSEIVDRKRKSRGTRDVAEILRQWREYNEQIEAESCIDGGGPKSIRKPPPKGSRKGCMKGKGGPENGICDYRGVRQRRWGKWVAEIREPDGGARLWLGTFSSSYEAALAYDEAAKAIYGQSARLNLPEITNRSSSTAATATVSGSVTAFSDESEVCAREDTNASSGFGQVKLEDCSDEYVLLDSSQCIKEELKGKEEVREEHNLAVGFGIGQDSKRETLDAWLMGNGNEQEPLEFGVDETFDINELLGILNDNNVSGQETMQYQVDRHPNFSYQTQFPNSNLLGSLNPMEIAQPGVDYGCPYVQPSDMENYGIDLDHRRFNDLDIQDLDFGGDKDVHGST

>AT2G40350.1 Arabidopsis thaliana MPRKRKSRGTRDVAEILRKWREYNEQTEADSCIDGGGSKPIRKAPPKRSRKGCMKGKGGPENGICDYTGVRQRTWGKWVAEIREPGRGAKLWLGTFSSSYEAALAYDEASKAIYGQSARLNLPLLPLCQARLLHFLMNLKFVHVRIQMQDLVLVRSD

>AT3G11020.1 Arabidopsis thaliana MAVYEQTGTEQPKKRKSRARAGGLTVADRLKKWKEYNEIVEASAVKEGEKPKRKVPAKGSKKGCMKGKGGPDNSHCSFRGVRQRIWGKWVAEIREPKIGTRLWLGTFPTAEKAASAYDEAATAMYGSLARLNFPQSVGSEFTSTSSQSEVCTVENKAVVCGDVCVKHEDTDCESNPFSQILDVREESCGTRPDSCTVGHQDMNSSLNYDLLLEFEQQYWGQVLQEKEKPKQEEEEIQQQQQEQQQQQLQPDLLTVADYGWPWSNDIVNDQTSWDPNECFDINELLGDLNEPGPHQSQDQNHVNSGSYDLHPLHLEPHDGHEFNGLSSLDI

>AT3G57600.1 Arabidopsis thaliana MEKSSSMKQWKKGPARGKGGPQNALCQYRGVRQRTWGKWVAEIREPKKRARLWLGSFATAEEAAMAYDEAALKLYGHDAYLNLPHLQRNTRPSLSNSQRFKWVPSRKFISMFPSCGMLNVNAQPSVHIIQQRLEELKKTGLLSQSYSSSSSSTESKTNTSFLDEKTSKGETDNMFEGGDQKKPEIDLTEFLQQLGILKDENEAEPSEVAECHSPPPWNEQEETGSPFRTENFSWDTLIEMPRSETTTMQFDSSNFGSYDFEDDVSFPSIWDYYGSLD

>AT5G05410.1 Arabidopsis thaliana MAVYDQSGDRNRTQIDTSRKRKSRSRGDGTTVAERLKRWKEYNETVEEVSTKKRKVPAKGSKKGCMKGKGGPENSRCSFRGVRQRIWGKWVAEIREPNRGSRLWLGTFPTAQEAASAYDEAAKAMYGPLARLNFPRSDASEVTSTSSQSEVCTVETPGCVHVKTEDPDCESKPFSGGVEPMYCLENGAEEMKRGVKADKHWLSEFEHNYWSDILKEKEKQKEQGIVETCQQQQQDSLSVADYGWPNDVDQSHLDSSDMFDVDELLRDLNGDDVFAGLNQDRYPGNSVANGSYRPESQQSGFDPLQSLNYGIPPFQLEGKDGNGFFDDLSYLDLEN

>AT5G18450.1 Arabidopsis thaliana MEEEQPPAKKRNMGRSRKGCMKGKGGPENATCTFRGVRQRTWGKWVAEIREPNRGTRLWLGTFNTSVEAAMAYDEAAKKLYGHEAKLNLVHPQQQQQVVVNRNLSFSGHGSGSWAYNKKLDMVHGLDLGLGQASCSRGSCSERSSFLQEDDDHSHNRCSSSSGSNLCWLLPKQSDSQDQETVNATTSYGGEGGGGSTLTFSTNLKPKNLMSQNYGLYNGAWSRFLVGQEKKTEHDVSSSCGSSDNKESMLVPSCGGERMHRPELEERTGYLEMDDLLEIDDLGLLIGKNGDFKNWCCEEFQHPWNWF

>Bradi1g72990.1 Brachypodium distachyon MSYGRKRSSWKKGPTRGKGGPQNAACEYRGVRQRTWGKWVAEIREPNKRTRVWLGSFATAEEAALAYDEAARRLYGPDAFLNLPHLRAVASGPAAAQHHQHRGQLVRWLPASSGARASPGGAAGVPAYGLLNLNAQHNVHVIHQRLQEIKNSSSKPASSKIITTPSPSDQLLLHPALPASSPSSTVTTTTNAMPPSAADSSVSCFQALELGVTGAETESAPCSEAHGFGGDKPQLDLKEFLQQIGVLRHDDNDGARGKDNGEAAAADGFGFGGNGGEFDWDALAADMSDIAGGHGVSGGLGLGVGVNGVFNMDDLEQFGCTYMPVPVWDI

>Bradi2g04000.1 Brachypodium distachyon MERGEGKRGAGDCSVQERKKKVRRRSTGPDSIAETIKKWKEQNQKLQGENGPRKAPAKGSKKGCMAGKGGPENSNCAYRGVRQRTWGKWVAEIREPNRGKRLWLGSFPTAVEAAHAYDEAARAMYGAKARVNFSEHSTDANSGCTSAPSLLMSNGPTTASHPSDEKDELESPPFVMSSAPTDGLHQPDAKDEYGSAGTLVHEVKTEVSNDLRSTCEEHKTAEVFQQEGNALHKEVKVSYDYFNVEEVLDMIIVELSADRKMEVHEEYQDGDDGFSLFSY

>Bradi2g29960.1 Brachypodium distachyon MTVDQRSVAAAPLEIPALQPGRTLGAEANTRSHVSVESIGSCTLPCNECELSAQQTPKGVAPVASILRKKRPRRSRDGPNSVSETIRRWKEVNQQLEHDPEGAKRARKPPAKGSKKGCMQGKGGPENTRCKFRGVRQRTWGKWVAEIREPNRVSRLWLGTFPTAETAACAYDEAARAMYGPLARTNFTIQDVPTPAVDIPAVVQRLLPGGSTSCESTMTSNHSGIVASSRVLEISSSLKQSDVGSEHDQRSNQYSSPQAGSSVARSRADDLFEPLEPIANLPDGEDDGFDIEELLRMMEADPVEAAEPMVENSWTGFQDVGANTVVDFDQQEPSYLDDFNPSMLEGMLQLAEPFPTCISEDRVMFNPGLRDADLSEFFEGL

>Bradi3g12680.1 Brachypodium distachyon MAERAASRGRQGNSRKCCPLRRSRKGCMKGKGGPENQRCPFRGVRQRTWGKWVAEIREPNRGARLWLGTFSTALDAARAYDSAAKALYGDCARLNLAGGTNNPVLAPPATAAATSSETQSYSSNSSSGANSDYYYYYNEYLNGNNGGGAWISSAAPPAVEDEDFDTYVRRLPKAEDFGLQAFLQNMPFDVLAEASGTAAGAGIWEPSCDMAAA

>Bra001386 Brassica rapa MSVNEQTGTDTSSKKRKARARADGKTVADRLKQWKDHNEGEESKPRGKVHAKGSKKGCMKGKGGPENTKCSFRGVRQRVWGKWVAEIREPNRVSRLWLGTFPTAEEAACAYDEAAKVMYGSSARLNFPCSEVATTSSQSEVCTVEDKGVVGGGDVCVKREDGDCESRAVSQIVDVKESCGDSRVQDTSVDERRDVVNSRLSSYLLDEFELDYQSRLTKELEEPKEEEDEVIQPQPQPQPELTVADYGWPNDMQNSVADYDWPNDMQNEPGFWDQDDLFDVDELLGDLDVDLLTGTDPSQNQNQEQVHPGGNDSHPFLLEPHNDICQNQEQVQPDGDDSHPFQLEPRDDPCQNQDHVQPGGDDSHPLDLEPHDGHEFFDLSFLDL

>Bra002159 Brassica rapa MEEEQPPAKKRNMGRSRKGCMKGKGGPQNATCTFRGVRQRTWGKWVAEIREPNRGTRLWLGTFNTSVEAAMAYDEAAKKLYGHEAKLNLLHPQQQQQKQCVNRNLSFSGHGSGSWGYKFDTVRGLELGLGTSNEARGSWSGGFSFLQEDDYHNSNRYVSSSGSNLSWLLPKRSSSQDQESVNAASGSGGQTFSTKLKPQNLMMTPSDYGSSNGVWSRFLAGQQENKIEYDVSSSCGSSDNKESVSAPSGESGDAGEGLHRPEVEEETGYLEMENLLEIDDLGLLIGKNGDFKNWCCDEFQHAWNWF

>Bra003752 Brassica rapa MSSIEPKVRMVGANKKQRTVNASSRKGCMRGKGGPENASCTYKGVRQRTWGKWVAEIREPNRGARLWLGTFETSREAALAYDSAARKLYGPEAHLNLPESLRSYPETASPGTQVSQTPSSNTSAKSSEESPRLSNEMSSWGTRSEISWEHMNVDLPVTDDSSIWEEATMSLGFPWVHEGNDDISLFNSCISDGYSNWDSFHSPL

>Bra005113 Brassica rapa MEKEDNGPNQSSSASIEPSRRRRRAAEPVDTTLSRWVKEEEEEEGLKRTRRVQAKGSRKGCMRGKGGPENPVCRFRGVRQRVWGKWVAEIREPVNQRGGNSKRLWLGTFDTAADAALAYDRAASAMYGRYARLNFPDGLGNGQDDEMKKTDEAESSRSYWLETCNVSETGNSVVVDKKDGEDYLYEDCIELGQDKIEKLGRMADNEIVKSEEDYMFDGFELDNGLLYNESGHYHGGGFEPYLEHFRF

>Bra005852 Brassica rapa MAVYFHSGDINKTQLDTSRKRKSRSRRDGTTVAERLQIWKQYNDNVEEASTKKRKVPAKGSKKGCMKGKGGPENGQCSFRGVRQRIWGKWVAEIREPNRGSRLWLGTFPTADEAACAYDEAARVMYGPMARLNFPQSSVTDVTSSSSHSEVCTAPGLVHVKTEDADYESNSFVEADAKNGAYTHEEKVKKDVKVDAGSDWLSEFEQKYWSEVLEEKEKQKKQVETCQKQPASLSVSDYGWPEDLDQSHWEMFDVDELLGDLNGDMFTGLDQSQCLAGSVGGGLSESEKKQIGLYPLQSLDSSYGLPPLQLDAQDDNEFVDLRFLDLER

>Bra006460 Brassica rapa MEEQPPAKKRNMGRSRKGCMKGKGGPENATCTFRGVRQRTWGKWVAEIREPNRGTRLWLGTFNTSVQAAMAYDEAAMKLYGPEARLNLLQPPQKQKQEVKRNLSFSDHGSGSWSYKLDTIRGLDLGLGPSNGSRGSWSGSFSIPQEDDHPNVSWLLPKRSSSQDQESVNDASGLAFSNKMQPRNLMMTPKHGSSNGVWSRFLVGQEKKTEYDVSSSCGSSDNKESILVPSVSGGEGMRRPEVEVEVGTGYLEMDDLLEIDDLGLLIGKDGDFKNWCCDEFQHPWNWFSERF

>Bra007341 Brassica rapa MEKPSSMKQWKKGPARGKGGPQNALCRYRGVRQRTWGKWVAEIREPKKRARLWLGSFATAEEAAMAYDEAALKLYGPEAYLNLPHLQQRKQQGPPLNNTQRFKWVPSKKFISMFPSRGLLNVNAQPSVQVIQQRLDELKRNGLLSQSYSSSSSSTESKSLLDEKTSKDTVLEGDGDKEKPEIDLTEFLQQMGILKDKSQPEASQVAESHPTPPWNEQEEGGSPFTAQSLSWETMIEMPGSETSAMQFDFDFEDDDLGFSSIWNFCGSLDE

>Bra008200 Brassica rapa MVGANKKQRTVHASSRKGCMRGKGGPDNASCTYKGVRQRTWGKWVAEIREPNRGARLWLGTFDTAREAALAYDSAARKLYGSEAHLNLPESLRSYPETVLPGTQASHTPSSNTGGKSSDSINEESPSSSNEMSSWGTTEEISWEHMNADLPVTDDSSIWEEATMSLGFPWVHEGDDISPFSTCINGGYSNWDSFHSPL

>Bra009112 Brassica rapa MAVYEHNKTEFDTTKKRKTRSRRDGTTVADRIKMWKEYNDTVQESPTKKRKVPAKGSKKGCMKGKGGPENGGCSFRGVRQRTWGKWGAEIREPNRGSRLWLGTFPTAEKAAAAYDEAAKAMYGPLARVNFPQASVSDVASTSSRSEVCTVETPGVVHVKTEDADCESPMARVENDVHEGAEEMKMDVNVHAAVDTPSKDWLSEIEQEYWTGLLEEKQKQKEQEMVAAGNFQKQPDALSVSDYGWPADLYQNQWNSLEMFDVSELLGDLNGDIFTDTKQSQCLGDNVGGGLPEPEKEQLQSLGSNYRLSPLQHEAQDGSDFFDLSFLDVKN

>Bra014617 Brassica rapa MEKPSSMKQWKKGPARGKGGPQNALCRYRGVRQRTWGKWVAEIREPKKRARLWLGSFATAEEAAMAYDKAALKLFGHDAYLNLPHLQRKQGPSVNNTQRFKWVPSKKFISMFPSRGLLNVNAQPSLHIIQQRLDELKRNGLLSQSYSSSPSSASFLDEQKKPEMDLNEFLQQMGILKDKSQAEASEVAVSHYTPPWKEQEESGGPFTDQSLSWETMIDEMPGSETSAMHFDCSNFGSYDDLEDDLGFPSIWNFCGSLDE

>Bra015840 Brassica rapa MSSIEPNVMMVGANKKQRTVQASSRKGCMRGKGGPDNASCTYKGVRQRTWGKWVAEIREPNRGARLWLGTFDTSREAALAYDSAARKLYGPEAHLNLPQESVRSYPETTQTPSSNSGGKSDCWVNKESPCSSNEMSSWGTREEISWENMNADLPVTDDSSIWEEATMSLGFPWVHEGDDNFSAFNTCISGGYSNWDSFNFPIFRFH

>Bra017011 Brassica rapa MGLHLWFRILVKEKTLAEKIVTFFSFFPETGLMFFTGHEFVQMGLVKEKTLAEKIVAFLSFFPETALAMRKRKSRDGATSVAETLNKWREDNEITEAASRNDDGCLKPKPIRKAPPKGSRKGCMKGKGGPENGIWNYRGVRQRTWGKWVAEIRQPCRGARLWLGTFPSSYEAALAYDEAAKAMYGESARLNLPDISNVSSLTTAAGSVTTLSNESEVCALEDTNVKEEDGGDEYGFVKSSQCVKEEMGVPDSADTFGYGNEHEAWDFGVDEMFDVDEVMGLLDEINVSGQETTQSQDASLLGSLNHMETAHPGVDCGYPIVQPSERNNSCVDLDRY

>Bra023674 Brassica rapa MEEEQPPAKKRNMGRSRKGCMKGKGGPENATCTFRGVRQRTWGKWVAEIREPNHGTRLWLGTFNTSVEAAMAYDEAAKKLYGHEAKLNLLHPQQQQKEKVNRNLSFSLTGTSWDYKLEKVHGLDLGLVPSSGSRGSWSGSFSFIQEDDKTTSESSVSWLLPKRSDSQDQESVHAASSLTFSTKLKPMMTPDYGLSNGVGSRFLVEQEKKTVYDVSSSCGSSDNKESILVPSVGVGEVMHRTEVEEGTGYLEMDDLLEIDDLGLLIGKNGDFKNWCCDEFQHPWNWFLE

>Bra028759 Brassica rapa MAVYDHSGDINSTQLDASRKRKARSRRDGTTVAERLQLWKDYNDNIEEASSPKKRRVPAKGSKKGCMKGKGGPENSQCSFRGVRQRIWGKWVAEIREPNRGSRLWLGTFPTAEEAALAYDEAARVMYGPLARVNFPHKSVSDDQSEVCTAGSPGRVEVKTEDVDCESEEVKKDVKADWLSEFEQRYWSEVLEEKEKHKKQELVVESCPKQPESLSVADYGWPEDLNQSQWDSSEMFDVSELLGDLDGDIFTGLDQVSYPLDNVAGGLPETEKLQGLDSSYGLPPFQLEAQDDNEFFDLSFLDLEK

>Bra029889 Brassica rapa MAVYDQTGTHTVSISRKRKSRARADGTTVADRLKKWKEYNDSVNASSIKQGEKPKRKPPAKGSKKGCMKGKGGPENSHSSFRGVRQRVWGKWVAEIREPNKVSRLWLGTFPTAEEAASAYDEAAMAMYGPLARLNFPQQCVGSESLTSTSSQSEVCRDENKAVLDVKQEDVDCKTRPVSEIKYVKEVCGDHAYTRLNEFDEEYWSRLSNGVEKPKEEEVIQPQQELDMLTVADYGWLSDMQNEQGFWDPDDSFDIDELLRDIDVGLLTGHDPSQNQSQVVHPGGYDSHPLQMEPQDNNHELFNLSSLDGSLTKF

>Bra034159 Brassica rapa MAIYEQTGINTSSKKRKSRARADGTTVADRLKKWKEYNVIADASSTKKRKVPAKGSKKGCMKGKGGPENTHCSFRGVRQRVWGKWVAEIREPNRVSRLWLGTFPTAEEAASAYDEAAKVMYGPLARLNFPQQCVVASEFLASTSSQSEVCSVEVKPVLVGDVHCESRPVSQISDGNTRMSSDLLNEFDEEYWGRVAKEIEKPKEGEEEVLTVADYGWSNDMLSEQDLWDPNEVFDVDELLGDIDECIMLTGTGVDEDQNGINPGGYDSHIPLQLEPHDGHEFFDLSSQDL

>C.cajan 05569 Cajanus cajan MGAYDQVSLKPLDSSRKRKSRSRGDGSRSVAETIAKWKEYNEHLYSGKDDGRPTRKAPAKGSKKGCMKGKGGPQNSQCNYRGVRQRTWGKWVGEIREPNRGNRLWLGTFSTAQEAALAYDEAARAMYGPCARLNFPHISDYASVKESLKESSLAASSSCSSAATAVSETTTTTSNQSEVCAAEDVKEKSREATHDAGQVAEDVNRDQMDFSWIDNFDFNDDYLKSFSTDELFQVDELLGHLDNNPIDDTGLMQGLDFGQMGFPGNGNNPQVDTPSSFFYQLQNPDAKLLGSLPHMDHTSSGVDYGLDFLKIVEPGDYNAGEPPQFLNLDDDLNNDSKGIQAMKHD

>C.cajan 09497 Cajanus cajan MDTCKKSLKPWKKGPTRGKGGPQNASCQYRGVRQRTWGKWVAEIREPKKRTRLWLGSFATAEEAAMAYDEAARRLYGPDAYLNLPHMQPTSTSPSGKFKWIPSKNFISMFPSYGLLNVNAQPSVHLIHQKLQELKRNSVVNQSPSSSSNEPKAETQNVNSKNDGEDPLKDAQTSSEELLGDLQEKEKPQIDLHEFLQQLGILREEREFGRTDSSVSSTVPEAVLRDDNAHQLEVFSDNCVNWEALIEMHGIAGIHQESEATQFEAYDPNDDLTFSTSIWNF

>C.cajan 17676 Cajanus cajan ERKSRKRRSGVSDSVEDTLEKWKEYNRKQQLGCRENGVEVIHKVPAKGSRKGCMRGKGGPQNSDCKFRGVRQRIWGKWVAEIREPINGKLVGEKANRLWLGTFSTAHEAALAYDEAAKAMYGPCARLNFPESNGSSLSSSGCDQKSPTASEVGDFEEETEEKPCVADESIEELKEMVTTGFEHQTSEECKVMSLSSVVKSQGFGRELEEVVRNLGEEGNQLQKEPMDIRDDYCSSFDAAEHEILVKSEETRGESVESFRSYSNPRPNSEACVAKKNTEEVISEILGLCHSKCLEMTPNEQYRNGDSNHPSMESMSQVEALNNINNTNRNSLHGFSSGHSRKLCDLSQQLHKHRNNMQFAELEVGYDYSFLRPDYDFGLLEEKKLLDVCFPRIGS

>C.cajan 18696 Cajanus cajan MGIEERKQLKKPAQASSRKGCMRGKGGPENASCTYKGVRQRTWGKWVAEIREPNRGARIWLGTFETSHEAARAYDAAALKLYGSDAKLNLPDLFTKPQCHSQFPPSPPPPPPPPSAAISQVENLQHSQIEDNIDMYPCSNFNGHPPVTMASQQVGVEPIYTSDSIMSLPLDTDPKPVETYPKPVWGTMNEGLPVFDDSIWTEAAMSLDFPLLAAETEIYALENMAEVMQSPWCM

>C.cajan 18697 Cajanus cajan MKSGVGIEERKQVKKRAPSRSRKGCMRGKGGPENASCTYKGVRQRTWGKWVVEIREPNRGARIWLGTFETSHQAALAYDAAARRLYGSDAKLNLPHLSIKPQSHSHSSQNNININMAACSNFNTNTNTMVPLPLDTNPKPLENEAEFRLWTPPVFDDSIWTEAAMSLDFPLLAPDTSENNSYIIN

>C.cajan 24232 Cajanus cajan MEGCKKSPLKPWKKGPTRGKGGPQNAACEYRGVRQRTWGKWVAEIREPKKRTRLWLGSFATAEEAALAYDEAARRLYGPEAYLNLPHMQPNFTTSKSQKFKWFPSRNFISMFPSRGLLNLNAQPSLHVIHQRLQQLRQNGVVAIHQSPDHSSSSTDPKVVQLDNLGCQNHAETLPPVQHDHAQTSPQKTLGDSEEKPQIDLLEFLQQMGILKEERGSEKTESTGSSTLSEAASRDDSEQPGVFSDMSVNWEELIEMHDHGVADNFLASEGIQFEACDINEDLTFSSSIWNY

>C.cajan 26554 Cajanus cajan MRKLVKKHNKGDGSKSLADTLAKWKEYNALLESSNEAEKPVRKVPAKGSKKGCMKGKGGPENSRCNYRGVRQRTWGKWVAEIREPNRGNRLWLGTFPTAISAALAYDEAARAMYGSCARLNFPNVQVSTLSEESSRNSPVAKQTGSALTILESSEFLVLPNNSRADTAEDDDMEGLSLSLSVKHEEGEGESGSGSSYPSVS

>C.cajan 33750 Cajanus cajan MHMLAKAHNKGDGSKSLAKILARWKEYNAQLDSCNDADKPVRKVPAKGSKKGCMKGKGGPENSRCNYRGVRQRTWGKWVAEIREPNRGNRLWLGTFSTAIGAALAYDEAARAMYGSCARLNFPNVSVSSFSEESSKDSPVANRSGSSMAVSANTGSMISPSNSGGGIEEDIDLEPISLSLSVKHENGEGESGG

>C.cajan 39702 Cajanus cajan MRGKGGPENAACTYKGVRQRTWGKWVAEIREPNRGARLWLGTFETSRDAALAYDAAAKKLYGSDAKLNLPEVHQPPNSNPPPSPPTPQMSPQPQIHNTTFDVVPDVNPSDFTNNMVNSNNPVLSMASHQVGDVPVYTSDSIVSLPFETTHNNVTLPYETINDHVSLPYGTNTHTNFSFYSNSFWGMMNETVPMLDESIWTDDAMTLDFPFVADDEIFGTGNFPDVGGWNYGEDDNVDDNSPPPPWM

>Carubv10001099m Capsella rubella MEPFVAVLDVKPPNKESCLLRKKIKSFTEKKIGARTLNTEATYLSKGREFDFDQTSSFPISSPIGCVVTRKEMAVYDQSGDINKTQLDTSRKRKSRSRRDGTTVAERLKRWKEYNDTVEEASTKKRKVPAKGSKKGCMKGKGGPENGRCSFRGVRQRIWGKWVAEIREPNRGSRLWLGTFPTAEEAASAYDEAAKAMYGPLARLNFPPSDGSEVTSTSSQSEVCTVETPGCAHVKTEDADCESKPLFGEANPMCHLESGADVNKKDVKADVDWLSEFENNYWSDVLKEKEKEKKEQEIVETCKQQPDSLSVADYGWPNDLDQSHWDPSEMLDVDELLGDINGDLFTGLNHDQYAGNNVDIGSFKPERRQGGYQSLQSLDYGLPPFQLEAKDGNGFFDDLSFLDLEN

>Carubv10002425m Capsella rubella MEEEQPPAKKRNMGRSRKGCMKGKGGPENATCTFRGVRQRTWGKWVAEIREPNRGTRLWLGTFNTSVEAAKAYDEAAKKLYGHEAKLNLLHPQQQQQQQQQQQVVNRNLSFSGHGSGSWSYKLDTVRGLDLGLGPVSGSRSSWSGRSSFLQEDDDHHYDRCLPSSDSNLCWLLPKQSDSKGQESVDAASGGGGSSLTFSTTLKPRVNPNYGLSNGAWSRFLVGQEKKTEHDVSSSCGSSDNKESMLVHSSGVERLHKPEVEEGTGYLEMDDLLEIDDLGLLIGKNGDFKNWCCEEFQHPWNWF

>Carubv10013771m Capsella rubella MLYIPLLFLNILFSGKKHEREEREESFRRRFDFCRFLTTNNIEQLREKEDFLFPIKEEVRLGILESFTIDSVIATKRKMAIYDQTGNNIPKKRKSRARADGLTVADRLKKWKEYNEIVDASTIIEGEKPRRRVPAKGSKKGCMKGKGGPENPHCSFRGVRQRIWGKWVAEIREPNRGSRLWLGTFPTAEEAASAYDEAARVMYGPLARLNFPQCVGSEFTSTSSQSEVCTLEDKAVLCGDICVKQEDTDCESKQYSQVLDFKEESGETRRDNCGVGHQDADTVLDYDLLKEFEQRYWGEAMTEEERLKQEEEERPKQEEEEKVKQEEEEMQQHQHQQQQQQLDLLTVGDYGWPWPNDMENEQSSWDPNEFFDIDELLGDMSEGMLHGPGQSEDQNNIDSGSYDLHPLHLEPHDGHEFDGLSSLDI

>Carubv10017800m Capsella rubella MEKSSSMKQWKKGPARGKGGPQNALCQYRGVRQRTWGKWVAEIREPKKRARLWLGSFATAEEAAMAYDEAALKLYGHDAYLNLPHLQRNTRPSLSNSQRFKWVPSRKFISMFPSCGMLNVNAQPSVHIIQQRLEELKKTGLLSSQSYSSSSSSTESKTNTSLLDEKTSKGETNNMFQGGDQKKPEIDLTEFLQQLGILKDESHAEASEVAECHSPPPWNEQEETGSPFGDQNFSWDTLIEMPRSETETMQFDSINFGSYDFEDDVSFPSIWDYCGSLD

>Carubv10020960m Capsella rubella MSSIEPKVMMVGANKKQRTVQASSRKGCMRGKGGPDNASCTYKGVRQRTWGKWVAEIREPNRGARLWLGTFDTSREAALAYDSAARKLYGPEAHLNLPESLRSYPETASSQASQTAPSSNTGGKSSDSESPCSSNEMSLCGRVAEEISWEHINVDLPVMDDSSIWEEATISLGFPWVNEGDNDISRFDTCISGGYSNWDSFHSPL

>Carubv10023556m Capsella rubella MGVLEQVANLASMPFDPPRKRKSRGRRDVAEILRQWREYNEKAETDSCIDGVLPKPIRKAPAKGSKKGCMRGKGGPENGICGYRGVRQRIWGKWVAEIREPGRGNRLWLGTFSSSHEAALAYDEAARAMYGQSARLNLPDYTNGSSSTAATVSGSVTAFSDESEICARQDTNVQVKLEDSSDEYVTLDSSQCMKEKLNVKEEERELNSVNAFGIGQESKKEILDDWVMGNGNDQEPLVFDMDETFDINELLGILDDNNASGQDTMQGQVDRQPNFSYQMQFPDANFLGSLNPMEIANPGIDYGYPYTQPSEMENNGIGSDHRRFEDLDIQDLDFGGGKDVHDSTT

>Carubv10023590m Capsella rubella MPSNIVERKRKSRGRRDVAEILRQWREYNEKAETDSCIDGVLPKPIRKAPAKGSKKGCMRGKGGPENGICGYRGVRQRIWGKWVAEIREPGRGNRLWLGTFSSSHEAALAYDEAARAMYGQSARLNLPDYTNGSSSTAATVSGSVTAFSDESEICARQDTNVQVKLEDSSDEYVTLDSSQCMKEKLNVKEEERELNSVNAFGIGQESKKEILDDWVMGNGNDQEPLVFDMDETFDINELLGILDDNNASGQDTMQGQVDRQPNFSYQMQFPDANFLGSLNPMEIANPGIDYGYPYTQPSEMENNGIGSDHRRFEDLDIQDLDFGGGKDVHDSTT

>Carubv10024477m Capsella rubella MDKEDNGSKQSSSASVGSSRRRRRAVEPVEATLQRWGKEEDGEGLERVRRVQAKGSKKGCMRGKGGPENPVCRFRGVRQRVWGKWVAEIREPVSHRGANSTRSKRLWLGTFATAAEAALAYDRAASVMYGRYARLNFPEGLLENEPGGEMKKKDEAGSSGSYWFEPDNVSEARDGMIETKDGKDYLLYDNHIELGQDKIENLDPTDTKIVETMAYNNPAVKAEEDYSFDRFELDSGLLYNEPEGPSYYQGGGSDSYLEFFRF

>Carubv10025253m Capsella rubella MDPLASKQQQQHHLDDTNQTLTHNNPPSDSTTTTTDSTSSAQRKRKGKGGPDNSKFRYRGVRQRSWGKWVAEIREPRKRTRKWLGTFATAEDAARAYDRAAVYLYGSRAQLNLTPSSPSSVSSSSSSVSAASSSPSSSSSSTQTLRPLLPRPAAATVGGGAAFGPYGIPFNNNIFLNGGTSMLCPSYGLLPHQQQQNQMVQVGQFQQQQYQNLHPANNNKIGDIELTDVPVANSTSFHHEVALGQEQGGSGCNNINNNNSMEDLNSLAGSVGSSLSISHPPPVVDPVCSMGLDPGYMVGDGSSTIWPFGGEDEYSHWGNIWDFIDPILGEFY

>evm.model.supercontig 1288.2 Carica papaya MEPPLPPPEPNNTTDTKSSDTTTSDNTTNTTNTTKNEDTVAKNDTTNSGSSGSSSTRRCKGKGGPDNSKFRYRGVRQRSWGKWVAEIREPRKRTRKWLGTFSTAEEAARAYDRAALILYGSRAQLNLQPSGSTSSSSSSSRGGSASSSSTTSSTQTLRPLLPRPSAFAFTFSSASSTSTLLMSTQHALQTPVAAELAASSSGFMPFGAYQNVVVGGSSAALLCPSMVMTSTSTATTQLPVFYQHYLQHHHHQYLPSDQINVGDISTHYHHHGPTSATTSYNQNPTPHRHHHHHQPQQQEEQEGCVFDVNSLVGSVDSSLSLSTQSAEAVIAPPPLPPANPDPGFSVGHGSTPVWPLTGSAEDDYPPCSIWDYPDPFGFLDLDF

>evm.model.supercontig 19.121 Carica papaya MGTFDQAPNVTSIAVDSSRKRKSRSRRDGTSVAETLKKWKEYNEHLDASGDGGKPARKVPAKGSKKGCMKGKGGPENSRCNYRGVRQRTWGKWVAEIREPNRGSRLWLGTFPTAVEAALAYDEAAKAMYGPCALLNLPHAEDLVCFGTASGSSVATPSGSDSTTTSSHSEVCAADDIKQEFVAIPGLKKEDGQGEARPNAYPHAVIETATPSSTMKLEAKDGDANIKKLHSGEYHGIKGEAVDYSKDVKSEGKEDESQDYWQTFSMDEMFDVDDLLGAIDNDPLVGTGLKQEFDYPPVQVGITDSRLQSEKSSALAYQLQNPDAKLLGSLHHMEQLPSTVDYGLALLQAQEHGDNNIELDGQGFMDMDIGLEDLDF

>evm.model.supercontig 5.232 Carica papaya MGKQSRGNCGESGSTSRSGRRRNGRDSVEDTLAKWKQLRFQLVGHGGNQKLPVKIQAKGSKKGCMTGKGGPQNSSYGYRGVRQRRWGKWVAEIREPISMDEVCTGKPGRLWLGTFSTAHEAALAYDHAATAMYGPFAHLNFPNPSVEALDNSSDKLGSSSSATATTHHLIPILHL

>evm.model.supercontig 51.23 Carica papaya MANTRRSSSSSAAAVSSGAKKPSMGRSRKGCMKGKGGPENALCTYRGVRQRTWGKWVAEIREPNRGTRLWLGTFNTSLEAATAYDEAAKKLYGASAKLNLQHNHIHHHHHHHQNYFPTTFSNNGNIGSDSSSSAGSSTESSVQGDQDLMMMTTNMVGAVSVDEHTRVNCSADGFLFSSSVVGEDQNMYWVPELAVENDFLELTDIGVLMGGGGNDDHHDELKGWNIGLQYPWSL

>evm.model.supercontig 92.16 Carica papaya MVVGEDKANSVLGVSLRERERMRMAWEKGEKKLKNQAVACEKRELVKMENVLRGDDMQAYHMSKAMFGTHRINKCVNVYITRISRRDDMQAYHMSNAMFGTHLLLLPQKTLPMENYRRSPFKQWKKGPTRGKGGPENALCQYRGVRQRTWGKWVAEIREPKKRSRLWLGSFATAEEAAMAYDEAARRLYGPDAYLNLPHLQPNINNNCPVKSQKFKWIPSKNFISMFPPCGLLNINAQPSVQVIHQRLQELQKSGVLSHTSSSSSSSCESKTTIQTLSDKTYDKHPASKDNDMEISSDKMLKDQEKPQIDLNEFLQQLGILKEEKQEEVNNTRENFKDEESSMKDHCELMAFADKSFNWDALIEMHGLPATQGVESSSFRVCNSQEEPTFTTSVWNF

>evm.model.supercontig 99.20 Carica papaya MCLPIFSNNTPKKKKKKRTRNNHSFPAAADRVLCLCWHLYTCIYIFIYVHINMSKSVMESSSRKGCMRGKGGPENALCTYKGVRQRTWGKWVAEIREPNRGARLWLGTFDTSHEAALAYDAAARKLYGPEAKLNLPQQPSNSSNNTHLIIPQMGNQFPNHRTSAPSCSSNNDSTVPCPDSKVESRGNLVDSNVKFGKDEDGMEEIWENMSLNLPVFDDSIWAEAAMSLGFPTMEDPGILGGNFVDTTGWDSLHSPPWCS

>XP 004487359 Cicer arietinum MIVKACDKGEVEGSKSVGKILARWKKYNAQLDSCNDADKPVRKVPAKGSKKGCMKGKGGPENSRCNYRGVRQRTWGKWVAEIREPNRGKRLWLGTFLTAIGAALAYDEAARAMYGSCARLNFPNVSVPRFSEESSNDSPAANHSNSSMTGSANSESMIMPNNLGIGAKDGNDMEPISLTLSVKQENEEGVN

>XP 004491005 Cicer arietinum MGAAYEQSGNVFLAPIETSRKRKTRSRGKGTRSVAETIAKWKEYNEHIYAAAGKDDAKQKRKAPAKGSKKGCMKGKGGPQNSECNYRGVRQRTWGKWVGEIREPNRGKRLWLGTFSTAQEAALAYDEAARAMYGPSARLNFPHVSDYSSIKESLKESTAASYSCSSVATTPATSEITTVSSHSDVFAVEDVKEIPKPVNMKNDNTVAVYREVYEASSPISRMKQEPKDEPADIIDPGGGEIQDGKSEGNQTQTLTHDVQIGEGVCNGQMDLSWIDDFDFNGDYLKSFSMDELFQVDELLGHIDNNPIDESGLVRSLDFGQMGFPEQSNPQVGTSSSFFYELENPDAKLLGSLPHMEHTTSGVDYGLDFLKTEEPGNFNIGVEDTPFLNLDYDMNHDMSHDSRGI

>XP 004492501 Cicer arietinum MKIDTVSGERKSRKRRSGGRTDSVEDTLEKWKKYNRQQQQKLGFGDNGVELVHKVPAKGSRKGCMRGKGGPQNSDCNFRGVRQRIWGKWVAEIREPINGKHVGEKANRLWLGTFTTAHDAALAYDKAAKAMYGPSARLNFPNGSPPSSTAGSAESLNGEDLAKAEELESNLRQSHEENKIFSNDFAVDDSEEAKEVIRNKKSKKMVHQGSYKNVKFETPRENERLESELEKVLENSGIGGECFHVQKEAINTDMNLGADCSSSDGVENGTLVKSEETAGGLVEDLKFFELSCSNHFLGNLQNMLPDSNTRPNSEHYDIKTKDSFEKKHKNEVTSAEIKEFCRGTGKSSRISHGQSQNEQNKNGHFDGMKSELKGLECKLRGQSNDEAQIVVPYMPGIHLFGGDSVGPIERMSQVEDLNYNTNKNTKLKEKGNNGSTLHGFSSGKSRKLSDLSQQLQKLGSYLPENWNNRQFADLEVGYDYSFLSPDYDFGLLEEKKLLDVCFSHIGS

>XP 004493523 Cicer arietinum MMDNCKKSPLKPWKKGPTRGKGGPQNASCEYRGVRQRTWGKWVAEIREPKKRTRLWLGSFATAEEAAMAYDEAARRLYGPDAYLNLPHLQPHSNSRIKTGKFKWLPSKNFISMFPSCGLLNVNAQPSVHLIHQRLQEFKQNAVATSQSSLSSSSNDPKAEEIQNVDGKKNHAENPIMEKDTQASSPNKMVGDFQEKPQIDLHEFLQQMGILKDDTNSEQTTDSSGSSTVHEAVSRDENDQLGIFSDMSINWEALIEMHGIAGTQESEATQLDGYDLTDQLNFSTSIWNF

>XP 004499266 Cicer arietinum MLKSGIGIEERKQLKKPAQASSRKGCMRGKGGPENASCTYKGVRQRTWGKWVAEIREPNRGARLWLGTFETSHEAALAYDAAARKLYGSDAKLNLPELSVTTNSTTTQQIPQINIPHNNNNIKNFSVSSTVHNNNSCFNNNNNNTNNSVFGAIYSSSDNSIVSIPLEQQVVESSYTKHMDHSDSFFGTVNYESTMPVVDDDSIWTEAAMSLDAAISMDFPMISDDDGIYSTGNFSEVAAWDSLQTPWCM

>Cla001733 Citrullus lanatus MPKLEKLQTRRPGSLSVAETLAKWKDYNDHLDSCNDEAKLARRVPAKGSKKGCMKGKGGPENMRCNYRGVRQRTWGKWVAEIRAPNRGSRLWLGTFPTAIDAALAYDEAARAMYGTLARLNFPNISIPTLLKEKESPTKDSPEIKRSSISFLGSTSLCLSSESTITSDHSEDCAVEDV

>Cla006605 Citrullus lanatus MALSDQYSDAISLPKNSIRKRKSRSRRDRSTVAETLAKWKAYNECFDSCNDGSKPIRKAPAKGSKKGCMKGKGGPLNSHCNYRGVRQRTWGKWVAEIREPNRGSRLWLGTFPTAIEAALAYDEAARTMYGHSARLNLPNIKNRGQLQGILLEDYLGLRNSDSSTATSTCSESTTTTSNQSEVCVPEEFAKRSQLVPSNIKIEDGEGESRTGDHSDGTATPMPLENEVKHEDCNDRAAVLGTEFPSLDQSQNFPMDEIFEPRTGNQADHMAMPMILEKQVKDEDLDAVYRDWSNDQAVLPEAGISSLNNLQNFQMDEMFDVEELLNLISSDSLYDPTTNILKGNADGYTNVAPSQVSNLGSEKPSSWSYQFQNPGAKLLGSLQQTEQAPADVDYGFDFLKQGREEDLNAAADDCVRYLNEMGDLGF

>Cla012000 Citrullus lanatus MTSENSSGEKISRKRRNGYVSVVDTLNKWKKLNNQLEDLAKDGVEEIRRVPAKGSKKGCMRGKGGPQNSDCNFRGVRQRTWGKWVAEIREPIASNNTRVKKKGSRLWLGTFATAHQAALAYDEAAKAMYGPFARLNFPDSSSSPLKPVIVEHSDSISPVASSSSSSSIFNGLSAEKMNCCYSMDKQENCGYESMEELKVKVEETARNRVNYTDATFYDSNIGNRLTEGNIKEGLADVVRSRDINRDSHDQNDPSELCFKFEAMDTNGCNDLNECNQYVLQKFQSDPYARTYWIPAEWEIGDLGSAPVMEGKPMEIESYGDCRAFNRDLSLLLDRQKHMGVGDQRVDDCNFEFLRPDYDFGLEEERKWLDLWFRG

>Cla017504 Citrullus lanatus MENYRDNKKPHSSSSSASSSSSSSSSLHKPWKKGPSRGKGGPQNASCEYRGVRQRTWGKWVAEIREPKKRTRLWLGSFSTAEEAAMAYDQAATRLYGPHAYLNLPHLANNNHHNNGSNSKSNNFLKWVPSKNFISLFPHTNRAAMAATGSFMLNLNAQPSLHLIHQRLQQLKPPHAFLSSNSLLSPSKKLEDEGEKEKDDEALVREEKTTKTTDTRSMEEEKPQIDLNEFLQQLGILKEEEEEEEKLVIELGEERNNNDDDGGYCLGSSESNNCDYSDEVEVLSDKSFNWDSIMEMDPNIEYHHFGSFQVYDHYVNYEDDLSFPNSIWDFEEDHSTRIIH

>Cla020801 Citrullus lanatus MDKKARNTTNVAQASSRKGCMRGKGGPDNASCTFKGVRQRTWGKWVAEIREPNRGARLWLGTFDTAHEAALAYDAAARKLYGAEAKLNLPQTTHRPPPHELPHQLERAAQSPSIIDSSPTATSSSSSSSPIGGLWGSENVNFDESIWREAVMSLDFPIIQDDQGIFFDGVGNWDTLQWCM

>Cla022458 Citrullus lanatus MRGKGGPENASCTYKGVRQRTWGKWVAEIREPNRGARLWLGTFDTSHDAALAYDNAARRLYGSDAKLNLPHLSPVSASSSSSDNVAANSNSNSNANAIVEDVASSVSHETNNNNTVSPKKRFREEEEEQIGGLWRAMSISLDDSIWVEAAMSLDFPIVMEHEQDHHHQGFFAANLMETNGNGGCGWDTLQWCL

>Ciclev10002280m Citrus clementina MSKALMGGIGEKKQLKKPAQASSRKGCMRGKGGPENALCTYKGVRQRTWGKWVAEIREPNRGARLWLGTFDTSHEAAMAYDAAARKLYGPDAKLNLPELPSAQSHHLPASSVNCTQHVAQRRNQPQMIHNSGITCSSNSPLPRSSNNDATQVYNFNYNNSAMSFLDNGSVGSHGKLAENEVKFSLNEQETDGFWENLNVNSPVFDESIWAEAAMSVDYPVMGEQGLLNANFVDASCCGWDMLPTPWCV

>Ciclev10005549m Citrus clementina MESYKKSPLKPWKKGPTRGKGGPLNASCQYRGVRQRTWGKWVAEIREPKKRTRLWLGSFATAEEAAMAYDEAARRLYGPDAYLNLPHLHTDNNNPSSNNKSHNKFKWVPSKNFISMFPSCGLLNVNAQPSVHVIHQRLEELKKNGVFNQTSSSSSSSGESKVEAPTTCIGDKTLLEAVSVREKEVEISSGNMLGWREEKKQIDLNEFLQQLGILKEESQAEVTEGTEGSSGAAESESSMKDYGELAAFADTSFNWDALIEMHGNGIAGHHQLGGFQLYDVQEEVTLPTSIWNF

>Ciclev10022244m Citrus clementina MEKVSEAASLSQKGTKKPNMGRSRKGCMKGKGGPENVLCTYKGVRQRTWGKWVAEIREPNRGARLWLGSFNTSLDAALAYDEAARKLYGDYAKLNLPQVQQHNRPSSNNINNNNHITASQVFTTDHIVPIQQQQQIESSNESVGNSSRFGESSVPGHFTSWISGEDNLYWPESLEANDFLDVNDSGVSLSDLMMGGNDLDWDGLQAPRSL

>Ciclev10032029m Citrus clementina MAIQSKDSSPSLMMPLSSDRKRKRRDGVNVAETLERWRRYNESLESGNGEDKPMRRVPAKGSKKGCMKGKGGPENGRCDYRGVRQRTWGKWVAEIREPNRGNRLWLGTFPSAVEAALAYDHAARAMYGPCARLNLPDVSRLNESSKDSDSTTSSNQSEIEDAKVKNDAREAESKIIAQPEAELLSSPVKPKAKDEAEDNEKYYWGEQLDCKPEARDEPILGSICDVKPETGDERKDATGIDWLEGYDWSKDEMFDVNDLLDLLDNNPLCGSEQQSELSYYNLQAGSLDQSQFGYEKPLDPFFQLRNPDMELPVGVNYAEHGASDYGLQFLKEEESDFGF

>orange1.1g019546m Citrus sinensis MAIQSKDSSPSLMMPLSSDRKRKRRDGVNVAETLERWRRYNESLESGNGEDKPMRRVPAKGSKKGCMKGKGGPENGRCDYRGVRQRTWGKWVAEIREPNRGNRLWLGTFPSAVEAALAYDHAARAMYGPCARLNLPDVSRLNESSKDSDSTTSSNQSEIEDAKVKNDAREAESKIIAQPEAELLSSPVKPKAKDEAEDNEKYYWGEQLDCKPEARDEPILGSICDVKPETGDERKDATGIDWLEGYDWSKDEMFDVNDLLDLLDNNPLCGSEQQSELSYYNLQAGSLDQSQFGYEKPLDPFFQLRNPDMELPVGVNYAEHGASDYGLQFLKEEESDFGF

>orange1.1g036655m Citrus sinensis MGGIGEKKQLKKPAQASSRKGCMRGKGGPENALCTYKGVRQRTWGKWVAEIREPNRGARLWLGTFDTSHEAAMAYDAAARKLYGPDAKLNLPELPSAQSHHLPASSVNCTQHVAQRRNQPQMIHNSGITCSSNSPLPRSSNNDATQVYNFNYNNSAMSFLDNGSVGSHGKLAENEVKFSLNEQETDGFWENLNVNSPVFDESIWAEAAMSVDYPVMGEQGLLNANFVDASCCGWDMLPTPWCV

>orange1.1g042353m Citrus sinensis MESYKKSPLKPWKKGPTRGKGGPLNASCQYRGVRQRTWGKWVAEIREPKKRTRLWLGSFATAEEAAMAYDEAARRLYGPDAYLNLPHLHTDNNNPSSNNKSHNKFKWVPSKNFISMFPSCGLLNVNAQPSVHVIHQRLEELKKNGIFNQTSSSSSSSGESKVEAPTTCIGDKTLLEAVSVREKEVEISSGNMLGWREEKKQIDLNEFLQQLGILKEESQAEVTEGTEGSSGAAESESSMKDYGELAAFADTSFNWDALIEMHGNGIAGHHQLGGFQLYDVQEEVTLPTSIWNF

>MELO3C003785P1 Cucumis melo MDRKSRTNNNVAQASSRKGCMRGKGGPDNASCTFKGVRQRTWGKWVAEIREPNRGARLWLGTFDTAHEAALAYDAAARKLYGSEAKLNLPQTTTTPHELPQLEHGGQSPSNNNIIASPSPTSSTTTMSSSPIERIGGLWENENVNFDESIWREAVMSLDFPIIEDDQGIFFDGAGSWDTLQWCM

>MELO3C008318P2 Cucumis melo MVEKMGHSDQYSDAISLPTNSIRKRKSRSRRDRSTVAETLAKWKAYNECFDSSNDGGKLIRKAPAKGSKKGCMKGKGGPLNSHCNYRGVRQRTWGKWVAEIREPNRGSRLWLGTFPTAIEAALAYDEAARTMYGQTARLNLPNIKNRGQLQGILLEDYLGLRNSDSSTTTSTCSESTTTTSNQSEVCVPEEFTVRPQLVPLNVKSEDGEGESRTGDHGDETATPMCLENQVKHEDCNGQTAALVAEFPCLDQLQNFQMAEIFEPRTGPQTGPMLTPLSLEKQVKDEDLDAVYRERSDDQAVLSEAGISSLYDLQNFEMDEMFDVEELLSLISSDSLHDPTNILKGNADGYTSMVPSQVGSIGSEKPPNSSYQMQNPDAKLLGSPQQMERTPADVDYGFDFLKQGREEDLNAAADDCVRYLNEIGDLGF

>MELO3C009669P1 Cucumis melo MENGRDNKKPHTSPFHHKPWKKGPSRGKGGPQNASCEYRGVRQRTWGKWVAEIREPKKRTRLWLGSFSTAEEAAMAYDQAATKLYGPHAYLNLPHLANHNNDSSDYKSNLLKWVPSKNFISLFPHTNRAAAAVATTGSFMSLHLIHQRLQQLKPPHSFLSSNSLSSPSKKLEDKGEKEKDEEASVREETTTKTTATRSVEEEEKPQIDLNEFLQQLGILKEEEDEKLEIELEGEKGNDNNKDKDNDNDDRGCCLGSSEVSNNCDYSDEVEVLSDKSFNWDSIMEIHPNIEDNHFGNFQLYDHHFLNYEDDLSFPNSIWDFEEDHSTRIIH

>MELO3C010977P1 Cucumis melo MLKSCSSNNKNNKKASSRKGCMRGKGGPENASCTYKGVRQRTWGKWVAEIREPNRGARLWLGTFDTSYDAALAYDNAARRLYGSDAKLNLPLISSSTSVSVSSSSSTTSDNNNNNSVVATASTYNNKVNPMVEDVASSSSSISNNKRFREDEEEQIGGLWRAMSISLDDSIWVEAAMSLDFPLLMEQQTSFSPNLVDTNPNPGWDSSIPWY

>MELO3C019763P1 Cucumis melo MPKLGKLQTRLPGSLSVAETLAKWKDYNDHLDSCTDEAKLARRVPAKGSKKGCMKGKGGPENMRCNYRGVRQRTWGKWVAEIRAPKRGSRLWLGTFPTAIDAALAYDEAARAMYGNLARLNFPNVSIPTLLKEKELSTKDSPDEIKRSSLSFLGSTSLSLSSESTITSDHSEDCAVEDV

>MELO3C026024P1 Cucumis melo MTSENSSGGKTSRKRRNGYVSVVDTLNKWKKLNNQLEDLAKDGGVEETRRVPAKGSKKGCMRGKGGPQNSDCNFRGVRQRTWGKWVAEIREPIASNNNTRLKKKGTRLWLGTFSTAHQAAHAYDEAAKAMYGPFARLNFPDSSSPPVMKPLTSEHSDTISPVASSSSSSSFFNGVPAEKMKGCYSMDKQENCEYESMEELKVKVEETARSRVHCTDIKPNSFYDSNIGNRSEGNIMEGLADVLRSHDQNSPSELCFKFEAMDTNGCNDLNECNQYVLQKLQSDPYGRTYWIPAEWEIGDLGSATVMEGKPMEIESYGDCMAFNRDLGLLLDRQKHMGVGDQRVDDCNNFEFLRPDYDFGLEEERKWLDLCFHG

>Cucsa.119440.1 Cucumis sativus MPKLEKLQTRRPGSLSVAETLAKWKDYNDHLDSCTDEPKLTRRVPAKGSKKGCMKGKGGPENMRCNYRGVRQRTWGKWVAEIRAPNRGSRLWLGTFPTAIDAALAYDEAARAMYGTLARLNFPNVSIPTLLKGKELSRKDSRDEIKRPSLSFLGSTSLSLSSESTITSDLSEDCAVEDV

>Cucsa.130200.1 Cucumis sativus MTSENSSGGKTSRKRRNGYVSVVDTLNKWKKLNNQLEDLAKDGGVEETRKVPAKGSKKGCMRGKGGPQNSDCNFRGVRQRTWGKWVAEIREPIASNNNTRLKKKGTRLWLGTFSTAHQAAHAYDEAAKAMYGPFARLNFPDSSSPLMKPLTSEHSDTISPVASSSSSSSFFNGVPAEKMKDCYSMEKQENCEYESMEELKVKVEETERSRVNYTDIKPNSFYDSNIGNRSEGNMKEGLADVLRSHDQNSPSELCFKFETMNTKGCNDLNGCNQYVLQKLQSDPYARTYWIPAGWEIGDLGSATVMEAKPMEIESYGDCMAFNRDLGLLLDRQKHMGVGDQRVDDCNNFEFLRPDYDFGLEEERKWLDLCFHG

>Cucsa.136780.1 Cucumis sativus MISETIERKRKSRSRRDRSTVAETLAKWKAYNECFDSSNNGGKLIRKAPAKGSKKGCMKGKGGPLNSHCNYRGVRQRTWGKWVAEIREPNRGSRLWLGTFPTAIEAALAYDEAARTMYGQTARLNLPNIKNRGQLQGILLEEYLGLRNSDSSTTTSACSESTTTTSNQSEVCVPEEFTMRPRLVSLNVKTEDGEGESKTCDHGDETATPMNQVKHEDRNDQLVALGAEFPCLDQLENFQMDEMFEPRTGTQAGHMVMPVSLEKQVKDEDLDAVYCGRSDDQAVLSEAGVPSLYDLHNFQMDELFDVEELLSLINSDSLHDPTNIVKGNADAYTNMAPSHVGSVGSEKPPNRSYQIQNPDAKLLGSPQQMERTLADVDYGFDFLKQGREEDLNAAADDCVRYLNEIGDLGF

>Cucsa.231820.1 Cucumis sativus MLKSCSSNNKNNKKASSRKGCMRGKGGPENALCTYKGVRQRTWGKWVAEIREPNRGARLWLGTFDTSHDAALAYDNAARRLYGSDAKLNLPLISSSISVSVSSSSSTTTSDNNNNNSVVATANTYNNDVNPTVEDVASSSSLSNNKRFREDEDQEQIGGLWRAMSISLDDSIWVEAAMSLDFPLLMEQQTFFSPNLVDTNPNAGLDSSLQWY

>Cucsa.336890.1 Cucumis sativus MENGRDNKKPHTSPFHHKPWKKGPSRGKGGPQNASCEYRGVRQRTWGKWVAEIREPKKRTRLWLGSFSTAEEAAMAYDQAATKLYGPHAYLNLPHLANQNNDSSDYKSNFVKWVPSKNFISLFPHTNRAAATVATTGSFMSLHLIHQRLQQLKPPHPFLSSNSLTSPSKKLEDKGEKEKDKDEEASVREETTTKTTATSSGKEEEKPQIDLNEFLQQLGILKEEEEKLVIELEEEKGNNNNKDNDIDNYNDDGGCCLGSSEISNNCDYSDEVEVLSDKSFNWDSIMEIHPNIEDNHFGNFQLYDHHFLNYEDELGFPNSIWDFEEDHSTRIIH

>Cucsa.342750.1 Cucumis sativus MDRKSRTNNNVAQASSRKGCMRGKGGPDNAACTFKGVRQRTWGKWVAEIREPNRGARLWLGTFDTAHEAALAYDAAARKLYGSEAKLNLPQTTTTSHELPQLEHGGQSPSNNNIIASPSPTX

>Eucgr.A02390.1 Eucalyptus grandis MDSSRKSPMKPWKKGPARGKGGPLNASCSYRGVRQRTWGKWVAEIREPKKRTRLWLGSFATAEEAAMAYDEAARKLYGPDAYLNLPHLCHQPPPDSSVFSSNRPLHHKFKWFPSKNFISMFPSCGLLNLSAQPNVHVIHQRLEELRKNGGFGGAASPSSSSSDSRIDTRRRTDLEVGKLKEKEVEILSEKAPATGHREEKPQIDLNEFLEQLGVLKEDNQSKEVVDVNGTEEGMFKDYEQIVAFDEKGINWDSLMEMHGYADQQIYAEGNTCQVYDISEEFALPSTIWNF

>Eucgr.F01607.1 Eucalyptus grandis MLKPGVGERKQGRKPAQASSRKGCMRGKGGPENASCSYKGVRQRTWGKWVAEIREPNRGARLWLGTFDTSHDAAVAYDAAARKLYGLEAKLNLPELYAAAPTTNLQPPAPAPTIANQAVHPLGNQAQIFHNLVLNPPSAPPVPVTQAMPISNDGLQAQGNDPVDPVPDNLKFGQNIEENDLWGNLNVNLPFLDESIWEEAAMSVDFPVLEDPVNFAGNFLDGNPWESSLPSP

>Eucgr.G03094.1 Eucalyptus grandis MSPEIVERKRSRSRKEGSMSVAETLAKWKQYNDQLDPQANGDKPARRVPAKGSKKGCMKGKGGPENTTFNYRGVRQRTWGKWVAEIREPNRGSRLWLGTFPTAIEAAKAYDEAATAMYGPCARLNFPTASHPMSFGDLDARATTPVGTTANTTGTPSTGYSAGTPSTGYSAGTPSEATTLSSQSELTGGPEVKDGDRKGESNADGGEMEPKSESIVDFDWLSDYAVDDLFDVDELLRTLESDPKFSSDVKMEH

>Eucgr.G03094.2 Eucalyptus grandis MSVAETLAKWKQYNDQLDPQANGDKPARRVPAKGSKKGCMKGKGGPENTTFNYRGVRQRTWGKWVAEIREPNRGSRLWLGTFPTAIEAAKAYDEAATAMYGPCARLNFPTASHPMSFGDLDARATTPVGTTANTTGTPSTGYSAGTPSTGYSAGTPSEATTLSSQSELTGGPEVKDGDRKGESNADGGEMEPKSESIVDFDWLSDYAVDDLFDVDELLRTLESDPKFSSDVKMEH

>Eucgr.K02952.1 Eucalyptus grandis MSPARQASFSVGEFGDKRLKKRRNGCDSVQDTLARWKDYYDSQAGLPEEEGGGGGGGGGGAVVRAARRVPAKGSKKGCMRGKGGPENSVCSYRGVRQRTWGKWVAEIREPIDKGSGAGQGKASRLWLGTFSTALEAALAYDAAARAMYGPLARVNFPGNSASSTPCRSDSSARRNFDDRVRFPA

>mrna01985.1-v1.0-hybrid Fragaria vesca MTNLKKVHTKQDGSSPVAEILAKWKEYNDHLESSNDGSKPTRKVPAKGSRKGCMKGKGGPENSRCNYRGVRQRTWGKWVAEIRSPNRGSRLWLGTFPTAIEAALAYDKAARAMYGSAARLNFPKVTFSSSGRATVLAPSCVATSAGSESSGTSGHSEDFSPQDKVNSHSPHIVNNDVIDLNQMDLNQTEEEG

>mrna08479.1-v1.0-hybrid Fragaria vesca MDTICLVHLDKYHCSHSRSHVSVFCMMKMLKSGMGSSVGVAERKTMKKPAQASSRKGCMRGKGGPENAMCTYKGVRQRTWGKWVAEIREPNRGARLWLGTFDTSSEAAMAYDAAARKLYGSEAKLNLPDQIVPQFPNPPPDHNNNLHMAPVVSNSSSNTAACFPPDISVPIMLQDRTNFPVQIPFPNPGMQTSYGIAYEQANSSSSSSGSNTTMPMDTKEEKYEGVDKRFWENVSTNVLPVFDDSIWAEAAMSLDFPVIENHGIFASNFVDGNAWEALPASWCA

>mrna13783.1-v1.0-hybrid Fragaria vesca MMPESSDRDSKKGKRLKVAKTLVKLEKCSESLGCAKDGLVNPVPRRMPTFGSRKGCMRGKGGPENMNYSYRGVRQRIWGKWVSEIRVPSSNTHGLKSKKVNRLWLGTFNTAVEAALAYDKAAKAIYGPVARLNFPESLETSMLDETTELGDNASEFEDGMVEGAKQEDEVLDATVKDPVNEWHCEANSVEELAINSVRESVEECHSELKYVKLELETKDEIAKDDVEVQAVAINNVWESEDEWPREPKYVKCELENEDQLVRNDVEVTVTTNNGREYENVWHWEPKYVNCDLETQDKLLQSNAEVERLAMNNVTDEEFQAGSYVGSSNNSHDNHLDKYLQVGKFDSVPNEKPCTNLDFSKILSRYSNDYLDTALVDVKCNSENDCNPSNDVIVDAPVMSKPMEKESVVILESGDNSLHMEPIYVASNNLIAKLGEYEKNVLPPNEFQGDIAETIKPNGHNGFGDNNICLQEHDEQIDVAYIPRPDIKPSKDIETQTDIDNNFSHLHSLSFDETYGTELHNLPKQLQAADGMPGSFNYREEECSGGDFNVDWLRQNYFSGEFEDNALPDSWFSYPHGGSS

>mrna16710.1-v1.0-hybrid Fragaria vesca MGRSRKGCMKGKGGPENATCTFRGVRQRTWGKWVAEIREPKRGARLWLGTFNTSTEAALAYDDAARKLYGSSAKLNLPDQSSSSASTSSKTSSGSSSNDKGGGGEHNNIKQEAYNSCDDVHRGWNNDYNNGEISDYCLWESNPVLSADWSGVCGEREFLEMNDIGVMMGGNEFVSCNMLL

>mrna21047.1-v1.0-hybrid Fragaria vesca MGAYNQGSNGFSQPLDSSRKRRRSKNDAGSVEETLSRWKEINRLKEAGIYGDKIRKVQSKGSKKGCMKGKGGPENQRCNYRGVRQRTWGKWVAEIREPNRGSRLWLGTFPTAVDAALAYDEAAKAMYGANARLNQPNVNNRNSYVSSWNESSHETSSVTTPSGCSTVAKQGCCESTTMSNQSDHSEVCAEEESVKVKTEDGEGELTSRAWSNAYTEPSASNATQDVDVKSEVPMDHAHTDQPGVQEQALDWIGFDVNYLNQFSLEELLFVDDLVGPTDDKPIPTLKLQQDTSSDVGRSQFADTAPVESATPSNSSYQVQYPDGTTSGSVQHMDQEPSVADYGFDLLNLQLQDGNVGVDNHRYFNMDDFDFHFPGGADN

>mrna26463.1-v1.0-hybrid Fragaria vesca MDNLRKSPLKPWKKGPTRGKGGPQNASCEYRGVRQRTWGKWVAEIREPKKRTRLWLGSFATAEEAAMAYDEAARRLYGPDAFLNLPHLQSNSSNLPLKSQKFKWFPSQNFISMFPPCGMLNINAQPSVHVIHQRLQELKQNGVFGQTTPSSSSSSCDSKPEAHVTMEKAQKENVAENKEKDVEITSEKVVGGLEEKPQIDLNEFLQQLGVLKNERQSEESDTTSFAAPESSITETSDGFGPFADKNFNWDALIEMHAMENQGAETGTFHIYDVNEELSFPTSIWNF

>Glyma02g31350.1 Glycine max MEGCKMFPPKPWKKGPTRGKGGPQNSSCEYRGVRQRTWGKWVAEIREPKKRTRLWLGSFATAEEAALAYDEAARRLYGPDAYLNLPHMMMQPTIFTNSTATKSHKYFKWFPSKNFISMFPSRGGLLNLNAQPSVHVIHQKLQELKQNGVVATSQTQSSPHTSSLSTHPKVVDLENFDIQNHAEILPPLQEGNSRVSSQKTIVGDFEEKPQIDLLEFLQQMGILKEEREEEKTNSSGSSTTVSEAASRDESSEQAGVFSDMSSVNWEELIEMHEHDHGVADSFYRASEEIQFEAYDINEDLTFSPSIWNY

>Glyma02g42960.1 Glycine max MGAYDQVSLKPLDSSRKRKSRSRGDGSKSVAETIAKWKEYNEHLYSGKDDSRTTRKAPAKGSKKGCMKGKGGPQNSQCNYRGVRQRTWGKWVGEIREPNRGSRLWLGTFSSAQEAALAYDEAARAMYGPCARLNFPKITDYPSVKESLKDSSMAASSSCSSAATAASDTTTTTSNQSEVCAVEDVIEKPANVNDKFNDCHKAYVSASPTSRMKQEPKDEAVDHMDTGAGEIQDVGLEGTHDTGQVAENVNKDQMDLSWIDGFDFIDDYLKSFSADELFQVDELLGLIDNNPIDNSVLMQGLDFGQMGFPGDGNPQVDDTPSSFIYQLQNPDAKLLGSLPHMEQTPSGVDYGLDFLKTVEPGDYNGGGEEPPFLNLDDDLNHDSNGMQARKGG

>Glyma03g29530.1 Glycine max MDTCKKFPLKPWKKGPTRGKGGPQNASCEYRGVRQRTWGKWVAEIREPKKRTRLWLGSFATAEEAAMAYDEAARRLYGPDAYLNLPHLQPRSTSTTITSSGKFKWFPSKNFISMFPSCGLLNVNAQPSVHLIHQRLQELKRNSVVSQSPPNSSNEPKEETQTVGSKKDGENPPKDDQMLSEEVLGDLQEKPQIDLHEFLQQMGILKEERESERTDSSGSSSTMPEALLRDDNDHHLGVFSDNSVNWEALIDMHGIAGIQESEVTQLEAYDPNDDLTFSTSIWNF

>Glyma04g03070.2 Glycine max MLKSGIGIEERKQLKKPAQASSRKGCMRGKGGPENASCTYKGVRQRTWGKWVAEIREPNRGARLWLGTFETSHEAALAYDAAARKLYGSDAKLNLPELSIKSQSQCPPPSPPPPSSVTNNTQMENLQQIQNNYIDMATCSNFNNINNPEVSMASQQVGVGPIYTSDSIVSLPLDTNPKPVENEVEFRPSWATMNEGLPVFDDSIWAEAAMSLDFPHIAAETAIYASGNNLADVGVWDSLQTPWCM

>Glyma06g03110.1 Glycine max MLKSGMGIEERKQVKKPAQASSRKGCMRGKGGPENASCTYKGVRQRTWGKWVAEIREPNRGARLWLGTFETSHEAALAYDAAARKLYGSDAKLNLPELSIKSQSQCPPPPSSVSNNTQIPQMENLQQQIQNNYMDMATCSNNFNNNTNNPSEVSMASQQVGVGVPIYTSDSIVSLPLDTNPKPVENDGDFRPSWGTMNEGLPVFDDSIWAEAAMSLDFPQIAAETAIYSSGNNLADVGVWDSLQTPWCM

>Glyma06g45680.1 Glycine max MHMLVKNHNKGDGSKSLADTLAKWKEYNAWLESNNEAEKPARKVPAKGSKKGCMKGKGGPENSRCNYRGVRQRTWGKWVAEIREPNRGSRLWLGTFPTAISAALAYDEAARAMYGSCARLNFPNVQVSTLSEESSRNSPAAANRSRNSPAASQSGHALMILESSECMILPNNSGGDAAEDDDMEDLSLSLSVKRVKHEEGEDESGTSSSYLSLS

>Glyma07g19221.1 Glycine max MNLDKLKVPRERKSRKRRSGESDSVEDTLEKWKEYNRQQQLGSRENGVEVIHKVPAKGSRKGCMRGKGGPQNSDCKFRGVRQRIWGKWVAEIREPINGKLVGEKANRLWLGTFSTALEAALAYDEAAKAMYGPCARLNFPEPIDSNGSSSSSGSDKKSPSGSSENGGDVAIAEELEVNHRRCHEDRPRFSKVGCVADDSIEELKEISNGFEQCQTSEECKATSLNDVKSEVPRENEGIEKELEEVLKNSGIGGEGRHLQKEPMDIPMNTRSNFCSSSASSDAATILIKSEETRGESVETLNSYELSCSNHCLGYMHNNMLPDTNPRPNSETSIAKKHTDHNKRLKISHSQSPNEQYRRSEHFDEMKTRLKGLECKLRAHSIHYKNQAPIVGDSNHPSMQGIHLFGGGTVGPIEGMSQVEALNNINTNRNSLPGFSSGHGRKLCDLSQQLHKLGGYLPEQWNSMQFPDLDAGHDYSFLNPDYDFGLYEEQKLLDICFPHVGS

>Glyma10g21850.2 Glycine max MEGCKMSPQKPWKKGPTRGKGGPQNSSCEYRGVRQRTWGKWVAEIREPKKRTRLWLGSFATAEEAALAYDEAARRLYGPDAYLNLPNVMMMQKIQPTASFTNSTITTTKSHKYFNWFPSKNFISMFPSSGGLLNLNAQPSVHVIHQRLQELKQNGVVATSQNQSSPHASSLSTHPKVVVELENLDIQNHAETLVPPLQEGNSQVSSQKTIIGGDFEKPQIDLLEFLQQMGILKEEREEEKTDSSGSSTTVSEAVSRDDQSEQQPGVFSDDMSSVNWEELMEMHEHDRGVVDNYHALEEIQFEAYDINEDLTFSTSIWNY

>Glyma12g11150.3 Glycine max MHMLVKNHNKGDGSKSLADTLAKWKEYNAWLESNNEAEKPVRKVPAKGSKKGCMKGKGGPENLRCNYRGVRQRTWGKWVAEIREPNRGSRLWLGTFPTAISAALAYDEAAMAMYGFCARLNFPNVQVSTFSEEPSRNSPAAAYQSRNSPSAKESGSALVILERSECMMLWNNSGGDAAEDDGMEDLSLSLSVKHEEGEDESGTSSSYLSLS

>Glyma12g32400.1 Glycine max MLAKTHNKGDGSKSLAKILAKWKEYNAQIDSSSDADKPVRKVPAKGSKKGCMKGKGGPENSRCNYRGVRQRTWGKWVAEIREPNRGNRLWLGTFPTAIGAALAYDEAARAMYGSCARLNFPNVSVSSFSEESSKDSPVANHCGSSMAVSANESMISPSNSGVGAEDDVDMEPISLSLTVKHENGEGESGISSSPPSP

>Glyma13g38030.1 Glycine max MLAKAHNKGDGSKSLAKILAKWKEYNAQIDSSSDADKPIRKVPAKGSKKGCMKGKGGPENSRCNYRGVRQRTWGKWVAEIREPNRGNRLWLGTFPTAIGAALAYDEAARAMYGSCARLNFPNVSVSSFSEESSKDSPSANHCGSSMAVSANESMISPSNSGVDAEEDVDMEPISLSLSVKHENGEGESGISSSPPSSS

>Glyma14g06080.1 Glycine max MGAYDQVSLKPLDSSRKRKSRSRGYGTGSVAETIAKWKEYNEHLYSGKDDSRTTRKAPAKGSKKGCMKGKGGPQNSQCNYRGVRQRTWGKWVGEIREPNRGSRLWLGTFSSAQEAALAYDEAARAMYGPCARLNFPGITDYASFKESLKESPMAASSSCSSAETATSDTTTTSNQSEVCAAEDVKENPRLVNVNDKVNDCHKAYEAASPTSRMKQEPKDEAVDHMVPGAGKILDVRPEGTHDAGQVAEDVNKDQMDLPWIDGFDFSDNYLNRFSTDELFQVDELLGLIDNNPIDESALMQSLDFGQMGFPGDGNPQVDDTLSSFIYQLQNPDAKLLGSLPHMEQTPSGFDYGLDFLKTVESGDYNGGGEEPRFLNLDDDLNPDSKGMQARKDD

>Glyma14g07620.1 Glycine max MGIQERKKVKKPAQASSRKGCMRGKGGPENAKCTYKGVRQRTWGKWVAEIREPNRGARLWLGTFDTAREAALAYDAAARKLYGPDAKLNLAELSVPAPALAAVNPSHMQQQPQVVPEMLIQQQQPLVHPTFDLVNVTSHPSGFNNILNNNPVVVSSMASSFLADAAVPVYTSCDSILSLPSATTNYTNTNTVPMEMENYSSSIPTVESNGDNMFRSFWGPTMDDTMPVFDQSIWTEAAMSLDLPIIADNGFYGRGVGGNKGGGNFFLDEVAAWDSLHTPSCCM

>Glyma14g32210.1 Glycine max MEERANKGEGGPQNASCEYRGVRQRTWGKWVAEIREPKKRTRLCLGSFATAEEAAMAYDEAARRLYGPDAYLNLPHLQPMSTSTIMSGKFKWFPSKNFISMFPSCGLLNRLQELKRNSVMSQSSSSSSNDPKAEIQNVDSKNHGEDENPPKDVQTSLEEVLGDLQEKPQIDLHEFLQQMGILKEERQSKRTDSSGSSTVCEAVLTDDCDNLGVFSDKSVNWEALIEMDGLAGIQESEVTQLEAYEPNDDLTFSTSIWNF

>Glyma17g37350.2 Glycine max MLKSGMGIQERKKVKKPAQASSRKGCMRGKGGPENATCTYKGVRQRTWGKWVAEIREPNRGARLWLGTFDTAREAALAYDAAARKLYGPDAKLNLPELSVPYAAGVMNPSHMQQQQQQQQQPQVPQMMMQQQQPLVHPTFDLVNVTPHSDFNNNSINILNNNPVVSSMASSFLADAAAPVYTSCDSIVSLPLSATNYTTNNTVPMEMDGYYYSSIAVESKEDDMFRPFWGPTMDDTMPVSDESIWTEAAMSLDYPIVGGGGGFGNNRKFFFGEATAWDSLQTPSFCM

>Glyma18g43750.2 Glycine max MKTNNNVSGERKSRKRRNGGSDYVEDTLEKWKEYNRQQQLGSRENGVEVIHKAPAKGSRKGCMRGKGGPQNSDCKFRGVRQRIWGKWVAEIREPINGKLVGEKANRLWLGTFSTALEAALAYDEAAKALYGPCARLNFSESIDSNGSSSSSGSDKKSPSGFSENGCDVAKAEELEVNRHRCHEEKPRFSKVGVFEETEEKPILSGGCVADDSIEELKEITTGFEQCQTSEECMATTLKNVKSEVPGESEEMERELEEVVKNSGIGGEVNHLRKEPMDIAMNARGNYCSSPGSSDAATEHEILLKSEETRGESVETLNSCELSCSNHCLGFMHNNMLPDTNLRPNPEASIAKKHTEEVISEILGLCQGKCLKISHGQSPNEQFRRSEHFDEMKTRLKGLECKLRAHSIHYKNQAPIVGDSNHPSMQGIHLFGGGTVGPIESMSQVEALNNINTNRNSLPGFSSGHGRKLCDLSQQLHKLGGYLPEHWNSVQFPDLEVGHDYSFLNPDYDFGLYEEQKLLDICFPHVGS

>Glyma19g04461.1 Glycine max MNTCKKSPLKPWKKGPTRGKGGPQNASCEYRGVRHRTWGKWVAEIREPKKRTRLWLGSFATAKEAAMAYDEAARRLYGPDAYLNLPHLQPRSTSTITLGKLKWFPSKNFISMFPSCGLLNVNAQPSRNSVVSQSSSSSSNDPMAEIQNVDSKNHGEDENPPKDVQTSSEEVLGDLQEKPQIDLHEFLQQMGILKEERQLERTDSSGSSTVREAVLTNDCDHLGGFSDKSVNREALIEMHGLAGIQESEVTQLEAYEPNDDLTFSTSSWNF

>Glyma19g32380.1 Glycine max MDTCKKSPLKPWKKGPTRGKGGPQNASCEYRGVRQRTWGKWVAEIREPKKRTRLWLGSFATAEEAAMAYDEAARRLYGPDAYLNLPHLQPRSTSTITSGKFKWFPSKNFISMFPSCGLLNVNAQPSVHLIHQRLQELKRNSVVSQSSSSSSNDPKAEIQNVDSKNHGEDENPPKDVQTSSEEVLGDLQEKPQIDLHEFLQQMGILKEERQSERTDSSGSSTVREAVLTDDCDHLGVFSDKSVNWEALIEMHGLAGIQESEVTQLEAYEPNDDLTFSTSIWNF

>Gorai.005G110100.1 Gossypium raimondii MCLSVINHFLSSPFPYSPQVPCWNNVNINDGWWLWRKKQARKPAQASSRKGCMRGKGGPENALCTYKGVRQRTWGKWVAEIREPNRGARLWLGTFDTSHEAAMAYDAAARKLYGSDAKLNLPELCANNPQSQPSSAANPQMAPMVNYQPQNVSNSVMPSFPNESIDLQGNVDAKFGQITEDGIEGFWENMNANLPLLDDSIWAETAMSLEFPMMGDPGSFASNLMEATGWDALQSPWCM

>Gorai.007G271900.1 Gossypium raimondii MEAQGKTNGERPSMGKSRKGCMRGKGGPENAMCRYRGVRQRTWGKWVAEIREPNRGSRLWLGTFNTSFEAALAYDAAARKLYGSSAKLNLPQPHDDHQTPCFTSLPGNFVASCKETGITMRQPLGNGLCVVESPPGSSVSSSQSSEERLVRREINTNEFKGSSLGDDGEEVFNWPEFSLENDFLEMSDIDVLMGQEFRDNWNGNEIAGIQNQWFF

>Gorai.007G359400.1 Gossypium raimondii MDSYKRSPLKPWKKGPTRGKGGPQNASCQYRGVRQRTWGKWVAEIREPKKRTRLWLGSFATAEEAAMAYDDAARRLYGPEAYLNLPHLQRIPTTTTTNLSNKSSTQKFKWIPSNNFVSMFPSSSPGLLNINAQPSVHVIHQRLQELKNNGVLKTKTDTQSKNIADDVQMKEKDVVGVSSDKPQIDLHEFLQQLGIKKDEKKQCEEEAVVNNNDDAESTLTAMDSTMKDYYDELVPFGENSSFNWDAMIEMHGGVDGFHGVEASFQVHDHGYEEDLAFPTIWNF

>Gorai.009G197800.1 Gossypium raimondii MSTSVMGGFGERKQIRRPAQASSRKGCMRGKGGPENALCTYKGVRQRTWGKWVAEIREPNRGARLWLGTFDTSHEAAMAYDAAARKLYGSEAKLNLPELCVGPRYPPPSTNTQAAAMGNQPQNLNNSGTCSSNSPTIIRTNDVQPVYNNDSVISFDSFPNENIDSQGNPAENNAKFGQNEDGIDGFWENMCVNLPVLDESIWAEAAISLDFPVMDDPGSFASSLVDVTGWDALQTPWCILYSYSNSHEYIMGFSGFVLA

>Gorai.009G271400.1 Gossypium raimondii MGGSGSGEKKLVKKRAQASSRKGCMRGKGGPENALCTYKGVRQRTWGKWVAEIREPNRGSRLWLGTFDTALEAAMTYDSAARKLYGSDAKLNLPHLCVNSRFPPPSSNTQVAPIGNQPLNATTSSPNSPIIIRVDEVTPVSNSESDMSQGNMVANNAKFGQNQEENGGFWENMTMNLPVLDDSIWAEAVMSLDFPVMDDPSIFGTSLVDTTGWDALQTPWCM

>Gorai.011G235900.1 Gossypium raimondii MGFKDNHNPEMDSSLFPGSSRKRKRRDGLSVADTLKLWSENSEAKQSRKAPAKGSKKGCMKGKGGPQNQNCNYRGVRQRTWGKWVAEIRAPNKGKRLWLGTFPTAVEAASAYDEAAKAMYGDKAILNMPQGSDSDSVATPSHGFSQATTTTTATCGGSESAMDGPVDSEAPSTSGAMDMKGEGGEVDRRGDDTDYSWLEGLESLQFFDDIPMDYVGNNSVWDNCELFDIDEFLA

>Gorai.013G025100.1 Gossypium raimondii MGFSENQNPNVGIPLFLDSSRKRKRRNGLSVADTLKQWSENSDTKKALKPPAKGSKKGCMKGKGGPQNQNCIYRGVRQRTWGKWVAEIRVPNKGKRLWLGTFPTAFQAALAYDEAAKTMYGEKAILNMPHGSDTTTTTSASSESTTATASDVKAEVREEAKVLAEEDESMKEMDYSWLNGLEFKDDIALNCGANNNISVWDGGWLFNEDDCFSIDELLG

>Jcr4S00053.270 Jatropha curcas MSKSIMTNSTIPEKKQLRKPAQASSRKGCMRGKGGPENALCTYKGVRQRTWGKWVAEIREPNRGARLWLGTFDTSHEAAMAYDAAARKLYGPEAKLNLPELHPNNNSRFPVETHSAQIQMGNQSQGQHNSSTMSSSPSSSSGSPIIRTNDVTPAYTHESSNTESNGTVVEEEANLWQNQQGIDDLWGNLNVNLPLFDDSIWAEAAMSIDFPAMEDTGIFASNLMDVTGWDALHTPWCM

>Jcr4S00137.180 Jatropha curcas MGQGYNVSSMPMEHTRKRRRRDSTCSVAETIQKWKEYNEFLDSCPNGDKPVRKIPAKGSRKGCMRGKGGPDNSRCNYRGVRQRTWGKWVAEIREPNRGPRLWLGTFPTAYDAALAYDEAAKAMYGNLARLNFPELSNSTSSSKDYMSSTTPSGYSSVATPAGSDSTTTSNHSEVCAFDDTKEHVVKIENGEGESKIKPEYPAVTDLVSPRCTPKQEVKNEEDDAKIPETKVPMKDDHKIPEIEVAVKDDLQHESKDDGTLNIEDYGWPNGLEGADFWKNFTVDEMFSVDELLGAIDKNPLDLDGQMLSTDNSDLQNDVVTPPLDLSFQLHSPYSKFNGNGNGDYGFDFLEPGRQEDNNIPLDDQGFFSLGGFET

>Jcr4S01985.20 Jatropha curcas MGQGYNVSSMPMEHTRKRRRRDSTCSVAETIQKWKEYNEFLDSCPNGDKPVRKIPAKGSRKGCMRGKGGPDNSRCNYRGVRQRTWGKWVAEIREPNRGPRLWLGTFPTAYDAALAYDEAAKAMYGNLARLNFPELSNSTSSSKDYMSSTTPSGYSSVATPAGSDSTTTSNHSEVCAFDDTKEHVVKIENGEGESKIKPEYPAVTDLVSPRCTPKQEVKNEEDDAKIPETKVPMKDDHKIPEIEVAVKDDLQHESKDDGTLNIEDYGWPNGLEGADFWKNFTVDEMFSVDELLGAIDKNPLDLDGQMLSTDNSDLQNDVVTPPLDLSFQLHSPYSKFNGNGNGDYGFDFLEPGRQEDNNIPLDDQGFFSLGGFET

>Jcr4S03169.20 Jatropha curcas MENCRKSPLKPWKKGPTRGKGGPQNAMCEYRGVRQRTWGKWVAEIREPKKRSRLWLGSFATAEEAAMAYDEAARRLYGPDAYLNLPHLQPSLINNKSNNSNKFKWIPSKNFISMFPSSCGLLNIHAQPSVHVIHQRLQEFKKNSNVSSSSSSDSKIIITEKPHVENPPLMEKQVEITTSEKPQIDLNEFLQQMGIIKVDRQQEEESDTTESCLVQESQVKEDEFYNNYNNNNNLGALADKSFNWDSLIDMHGIGGGGDYDQGVESSGTLQVYDAQDELAAFPASIWNF

>Jcr4S04186.20 Jatropha curcas MGERKVSSCAEIVSSKNKKIPRKRVEDTLEEWRSEIDEIPKRRPKGSRKGCMRGKGGPENQSCNYRGVRQRIWGKWVAEIREPAGKGPRNRHWLGTFETAVEAAQAYDNAAKTMYGSNAVLNFPDYSSEISSSKLNLLDCSGFDGFEQLRDEKLENCISTTDCRISHGVEQTNAEFAEDGYGSSQGETKITKAELHMDFDDATTDYMSFNEAEMPIMRKVMEGEISRTESDFMVRHDNVSNDCTDINCYTETDIKPSTHQDGNENSVLKPEGNYDYKDFELGFTNFQQGNCSSNISCEMQNPSANLPDSLNHTKERNRNFESNFDPMEPDFNWDSVEEELGLSIEPWFPEFGF

>Jcr4S09760.20 Jatropha curcas MRRSRKGCMKGKGGPENALCTYRGVRQRTWGKWVAEIREPNRGNRIWLGTFNTSDEAARAYDQAALKLYGSSATLNLPQYCFNLPIGTASNSNISAGTCQNVIGSSNNEEISSSCASMCQDLIGSCSSEVITGGARNSNTCAIGCESCSSSSCLIGGLGDNDFYWPQDELDIENHFMDNTFFGGAGIGGGTGIGGDGFSWDGSGNTWGGAWGF

>Jcr4S13987.10 Jatropha curcas MDSNPSQDPITTATTTSETITTTTIAAGVTSDTTSDSNNTSSDSAGKKCKGRGGPDNSKFRYRGVRQRSWGKWVAEIREPRKRTRKWLGTFASAEDAARAYDRAAIILYGSRAQLNLQPLNSSSAQNSSSSSSSRGGGSGGNGSSSSSTQTLRPLLPRPSGFGFSFSLSSTSMASPAAVAAASSGFVPYGVYQNLPNVVGSSTVLCTNNIVQNPQEQQITMSSNIPYQYQYQNPLSDYTSSLSAGHDPSSIPTTSYQNLNYDHNPHHQQQQEHGLYEDISSLVGSVGSSLSLSSNTQPIIAPANQDPVMHVGPGSPSLWPLTNDDEYPPSSIWDYGDPSLFDL

>Lus10002855 Linum usitatissimum MEDFSSIQKRTQRRRSCGGEESVEETLERWKKQTKLLKKKNPPKGSKKGCMRGKGGPENLTCSYRGVRQRTWGKWVAEIREPVLITGSTKKKGSRLWLGTFDTAVEAAMAYDNAARSMYGGSAVLNFPNGVVNDDNQSSSPATETETEISSASLDAGVVTTEELIKREVAEEELEVQNTKMMNSTEGENLQDEGLDVELEEFMKYCSSWALILNTEEEEEEEKMVKLEDVNGNEQTNSGGTTTTTTTTDAAYHTDFGRETSLNPDVYPCGGSKAFEEPFRIDTGVEWSGAEVIDWSMVEEEPGVPSDMMLFHDPEFGCY

>Lus10003581 Linum usitatissimum MEQDQQGSKRPKKALVVRSSRKGCMRGKGGPENANCSYRGVRQRTWGKWVAEIREPNRGNRLWLGTFNTSLEAARAYDRAALSLYGPSVTLNLPEENVGSSSASSSVAAVDIAVHDDSQEVVVKGDDGGEGLVDWMEFANEFVELDSEWRQHGVGMGSCLNSWDGGGFVENCPWSF

>Lus10006127 Linum usitatissimum MPTATSSSTSDHCPKPRASSGSSRKGCMRGKGGPENALCTYKGVRQRTWGKWVAEIREPNRGARLWLGTFDTSHDAALAYDAAARKLYGPDAKLNLPHFHNSPNNTNNNINIVPHVDVHVPLSVGCSSLTSSGGPTLSPSFTSTSSSSNNGPTFTSNNDDVAPSADVGYRGFDLALHGSGGGQWVDHYSSSDWNNTDSSSAAAADASCNYNYKVRGNEVWSNLNANLPLMDDSIWVEAAMSLDYFQVAAMQDSNGWDTGSLPPSNTTPWCM

>Lus10010632 Linum usitatissimum MSTSSSSTGEPGSNWNPHMKKAAQASSRKGCMRGKGGPENALCSYKGVRQRTWGKWVAEIREPNRGARLWLGTFDTSHDAALAYDTAARKLYGPHAKLNLPNHNDVVHVPKQDSGDATTTTSSSFDMPAGYYNNYSSNSSSEEGISSSSYDNYNEIGVGNEFWSNLQVNLPLFDESIWAEAAMSLDYFPVVAMDGGQLAGGWDAASASAPN

>Lus10011319 Linum usitatissimum MENCRNSLKPWKRGPTRGKGGPLNATCEYRGVRQRTWGKWVAEIREPKKRSRIWLGSFATAEEAAMAYDEAARRLYGPEAYLNLPHLLQQPTHHHHPSSTTTAAFHRYRWMVPSSRTSSVSSLFPPRGLLNLHAQPSVHVIHQRLQELKDKKNNSQKNNTPPPIIGSSQDSRAEPSSRVPEAMEEAEKPQIDLNEFLQQMGILKDERVVAADVPVEEEASRCYDNGELVGNSSLSFGEDKSLSYWDAFVSEDRGYYEGDNFQFRQDAVQEEELPFSAHIWNF

>Lus10012210 Linum usitatissimum MENCRNSLKPWKRGPTRGKGGPLNATCEYRGVRQRTWGKWVAEIREPKKRSRIWLGSFATAEEAAMAYDEAARRLYGPEAYLNLPHLLQQPPHHHHHHLSSTTTAAFHRYRWMVPSSRTSSVSSLFPPRGLLNLHAQPSVHVIHQRLQELKDKKNNSQKNNTPPIIGLSQDSRGAEPSTRVPKAMEEAKEKPQIDLNEFLQQMGILKDENRVAAADVPVEEEASRCYDNGELIGSSSLSFGEDKSLSYWDAFVSEDRGYYQGDNFQFLQDTVQEEELPFSAHIWNF

>Lus10012226 Linum usitatissimum MEDFSSIQKRTQRRRICGGDESVEETLERWKKQTKLLKKKNPPKGSKKGCMRGKGGPENLTCRYRGVRQRTWGKWVAEIREPVLMTGSTKKKGSRLWLGTFETAVEAAMAYDNAARSMYGGSAVLNFPNGVVNDDDQSNNPSSSPATETGTEISSASFDEGVVTMEELIKREVAEEEPEVQNTKMMNGTEGVNLKDEGLDGELEEFMKYCSSWALNSNTEEEEEEEEERMVKLEEVNGNEQTNSGGTTTTSDVAYHTDFGRETSLNPDVYPCGGSSFEEPFRIDTGVEWSGAEVIDWSMVEGEPGVPPDMMLFHDPEFGFY

>Lus10023633 Linum usitatissimum MAAFNQASSSDSSRKRRRRRTGSESVAETLKKWKQYNEYLDSENNKSGRRVPAKGSKKGCMKGKGGPENSKCKYRGVRQRTWGKWVAEIREPNKGPRLWLGTFPTAYEAALAYDDAARAMYGPGARLNLPAQASSTCDSATTTTTTRSEVCGNDQIVPGGDGEGESKLAKQESAEEEENDHLVLTGAKLAPQVKGEPLDVKDYEWQNFTGDEVFSVEELLGELENHPLDDAGLLLGDNNNTHTQGYLQCSNSDPDPDPRFIGGSQHKEPGPLMSFDHGFEFLEEDKNLVAPVDFSFGLGDLVFEQFDA

>Lus10024298 Linum usitatissimum MPNATSSSTSDHCPKPRASSGSSRKGCMRGKGGPENALCTYKGVRQRTWGKWVAEIREPNRGARLWLGTFDTSHDAALAYDAAARKLYGPDAKLNLPHFHNSPNNTNHNNIVPHVDVHVPLSLISSGGPTLSPSFTSSSSSNNGPTFTSNNADVGYRGFDLPLHGGDGGQWVDHYSSSDWKNTDSSAAAAAADASCNYNYKVGGNEVWSNLNANLPLMDDSIWVEAAMSLDYFQVAAMQDSNGWDTAALPPSNTTPWCI

>Lus10034902 Linum usitatissimum MAAFNQASSSDSSRKRRRRRTGSESVAETLKKWKQYNEYLDSENSSKSGRRVPAKGSKKGCMKGKGGPENSKCKYRGVRQRTWGKWVAEIREPNKGPRLWLGTFPTAYEAALAYDDAARAMYGPCARLNLPAQTSSTCDSSTTTKTTRSEVSGNDQIVPGEEGEGESKLAKQESAEEGENDRLVLTGTKLAPQVRGEPLDVKDYEWQNLTGDEVFSIEELLGELDKHPLDAGLLLGDNNNINTQAYLQCS

>chr1.CM0195.60.r2.d Lotus japonicus MEIEARKKLKKTAQASSRKGCMRGKGGPENASCTYKGVRQRTWGKWVAEIREPNRGARLWLGTFETSHDAALAYDAAARKLYGSDAKLNLPELRKSSSQHCPPFSSGSTQMENQPQILHHYHNMDMATCSGINANPTVSMVSSQQVGAGPIYTDSIVSLPSDTMPMPHEIETSYANVMEKMPHEMETSYANVMEKVPHEMEKSYANVMEKEEQSYPVFDNSMPMNEDLPVFDDSIWEEAAMSLDFPMINPEASTGNLADDSAWDPLQTPWYM

>chr1.CM0318.160.r2.d Lotus japonicus MDHTCKRSSALKPWKKGPTRGKGGPQNASCEYRGVRQRTWGKWVAEIREPKKRTRLWLGSFTTAEEAAMAYDEAAWRLYGPDAYLNLPHLQPSSNAAPIKSGKFKWLPSKNFMSMFPTCGALNVNAQPSVHFIHQRLQELKQNAVVTQPPSKEEIMTVGSKNNAEKPSAEKDAQTLPEAMLGDLQEKPQIDLHEFLQQLGILKEERHSERADSSGSSTVNEAVSRDDNDQLGVFSDNSVNWEALIAGIQESEDIIQLEEAYDMNDELNFSTSIWNF

>chr1.LjT01D01.90.r2.d Lotus japonicus LIFLDWNCCRDRRSRKRRVGGRVDSVEDTLEKWKKYNKQQELGVGEDGVEKIHKVPAKGSRKGCMRGKGGPQNSDCKFRGVRQRIWGKWVAEIREPINGKHAGEKANRLWLGTFSTAHEAALAYDEAAKAMYGPCARLNFPESRPNGSSSPSGSSDTLSGGGDLAKAVELEGNLHQSSGEKLRFSAVGVFDESAERMVPSEACVFDDSVEDSKEVTNDDFQCRTNENCKSITQQGSLKNAKYEISRDNDGKKKESEVNLNNYVWKNLQEGPVVVAMNSRAGYRSCDATEHEILVKSEGTKGESVEPLNSLELSCNYHHLHNMLPERDLRPNSEHFNSIQCEPIAKKHWKEVISEILELCCSSNNSKISHVQSQNEPCKNESFDEMKTGLKGLECKMRVHSIGFKNEAPIMEDSNHSIQRIPLFGGGGATVGLNERMSQAEDLSTKTHRISRLQEKGNYGNALPGFSSGQGRKLCDFSQHLQKLGEHWNNKQFADMEVSYDYSFLSPDYDFGLLEEKKLLDVCFSHLGS

>chr2.CM0002.480.r2.m Lotus japonicus MDDATTTPHHPPQEITKPTATAAITTTPSETSNSENSNSSHNNNITTPNTSTTINNRKCKGKGGPDNNKFRYRGVRQRSWGKWVAEIREPRKRTRKWLGTFSTAEDAARAYDRAAIILYGSRAQLNLQPSPTSSSSQSSSSSSRASSSSSSTQTLRPLLPRPSGYGFSFSGSHLPVVFSAAASGPFGFYNANNGYMQLQPQQHHFHHQEVVQSQLQPQLQQCRQPEPDVKGGGVVDHVRSTSYQNQHSHHHQDQVVMQNYPVLNHQQHNQNCMGEGVSDNTLVGPSLASQNFVHIDAAPMDPDPGSGIGSPSIWPLTSTELEDYNPVCLWDYNDPFFLDF

>LjSGA 063888.1 Lotus japonicus MMKSGMGIEERKQLKRPAQASSRKGCMRGKGGPENASCTYKGVRQRTWGKWVAEIREPNRGARLWLGTFETSHEAALAYDAAARKLYGSEAKLNLPELSVPAANSHHQSVPDTTITTAPANNQVVVPQMHQQQPQIHQNFDDAAVTSVLTSSDFSSNMMMNNPAISMPSHHHHQHVVGDGSMYSSDSVVPLLPLETNNNTNAAAMDTTDDMFRSFFGTVNNETMPPVFDDSIWAEAAMSLDFPVVADDGIYTASGNFGEVGVWDTLQTPWCM

>MDP0000139446 Malus domestica MSKWEMSDGSSSVGVPEKKPAKLPAQGSSRKGCMRGKGGPENAMCTYKGVRQRMWGKWVAEIREPNRGPXLWLGTYDTSYEAALAYDAAARKLYGSEAQLNLPDQQAPKYHPHQLNQHPQHQVFPSSANPRVPPQIPNQTLVQNNPSGTGTNPACYAPPVKDIAVPVIHNNFPNAKVDQPHGNYNSGGAETLPEQGGRKVEKNMSSGVNNEGMDGIFWGNMSDNFPVFDDWIWTEEAISLDLPVVAYQGIFDHGFVDGNCWETLHRPNGA

>MDP0000141335 Malus domestica MLKSEMNGGGSTSVGVEERKPSKWPAQASSRKGCMRGKGGPENAMCTYKGVRQRTWGKWVAEIREPNRGARLWLGTFDTSDEAALAYDVAARKLYGSVAKLNLPEQRVPHCHQHHLNQHLQNQVVPSPANPCVPPQIPNQTLVQNNPSGTGTNPAGYGAXVNDIAVPFIGDNFLXVKADQPHGNWAVALPEQGGTEVGENMSSGGNNEMEGILWGNTSGNFPVFDDSIWTEAAMSLDLPAVEDYGVFGGGFVDGTGWETMHSY

>MDP0000147009 Malus domestica MGAYDQGANMSSLPLDSSRKRKTRSRRDGNSVAETLEKWKEYNKKLESVNNEGKTRKVPAKGSKKGCMKGKGGPENARCNYRGVRQRTWGKWVAEIREPDRGSRLWLGTFPTALDAALAYDEAAKAMYGTGARLNLPHAANYHPSSSLETSSVATPSGSSAVATPGCSTSTSTSSRSEICGDEDSKLFLNVKKEDGEGESRMYPWSTAVPQASGMVKPEIIEDFTMDYLRNXQQQPVVKPEAGVEDHNWNGGEGFIGDYSENFTTDELFDMDEMFDVNELLMPSDDISLCNSGSEQVWRADVGQSGMETLSSERPSNLSYQLQFPDAKLLGSLQHMEHAPLNFEYGFDFKKQEKEGSNHTGQDDQGYFNLGLSDLDLEGFTGGITQSGDGGYNYSMEM

>MDP0000153866 Malus domestica MESEASDGERKLRKRRNGCESIEDTLTKWKNYNERLDSGKDGGKKTRAPAKGSRKGCMRGKGGPENSDCVFRGVRQRTWGKWVAEIREPIRARAGSVPTKKNRRLWLGTFPTAYEAALAYDKAARAIYGALARLNFPDNAVDSKDYYSNSVSSKTPSSHESSLTYNNADXAGGRSGFFEDCVAKEQKQETDCSVSEELHVLRATSREPKAVKCEYETERELVKNDDVFQTESYGSFDHRGDYLPNEPLVVNFDTVFDCKPCNDMDPLEXLLRSNYDYLTELVDGECNRRNSCKPSNDVKVETPAMREAAEKPFPVILESGSHNGXDEKYNNIHGEQINAAEILVTNFEPSEDVEMKLSMTNQELQGGFAETTRLDGHNCNGFIHSYACLDDLDVAYGPRYGINPWNDIGMQTELDDRLDYVHNWSAEETYGIDAAEDQQWGRTHNLPIQLQTQPHPDIPGSSNHTEYAHLGVDIDRQSYDSGAMEEQGLPK

>MDP0000165880 Malus domestica MGAYDQGVNMPSLPLDSSRKRKTRSRRDGNSVADTLEKWKEHNKQLESATEEGKKRKVPAKGSKKGCMKGKGGPENARCNYRGVRQRTWGKWVAEIREPDRGSRLWLGTFPTAVDAALAYDEAAKAMYGDGARLNLPHAVNNHPSSSQETSSVATPSGSSAAATPGCSTSTSTSSLSEVCADENSKLFLDVKKEDGEGESRMYPLSGAVPQASGMVKQEVNEDFDMDYLRNNQQQPVGMVKPEVNEVFDMDYLRNNQQQRGVEDHNPNGGDGFVGDYSENFTMGELFDMDEMFDVNELLMPPDDISLCNSGPEQVSRPDVGQPGMETLSSEGPSNLSYQLQYPDAKLLGSLPHMEQAPLDFEYSFDFMKQEEGSNLTSQNDQGYFNLGPSDLGLGGFTEVELVNATEGSFDIAFRNIRMFKDVGWIPPHQLGFHGGVSLLHSFGAFRFGGSAWFGSLAHSSSWGSTWLERLGATRLVAALLGSALAWTLKFHCLDFAGDLGLSASVPLLLSCSSDLSRCSLVPVALVHRGDAGVLANLVLAFVCCYCESRRLTMTLLWSRTFAMILCGLVIQ

>MDP0000223609 Malus domestica MSKWEMSDGSSSVGVPEKKPAKLPAQGSSRKGCMRGKGGPENAMCTYKGVRQRMWGKWVAEIREPNRGPRLWLGTYDTSYEAALAYDAAARKLYGSEAQLNLPDQQAPKYHPHQLNQHPQHQVFPSSANPRVPPQIPNQTLVQNNPSGTGTNPACYAPPVKDIAVPVIHNNFPNAKVDQPHGNYNSGGAETLPEQGGRKVEKNMSSGVNNEGMDGIFWGNMSDNFPVFDDWIWTEEAISLDLPVVAYQGIFDHGFVDGNCWETLHRPNGA

>MDP0000225376 Malus domestica MEEDQHRDSAAEAAPDHHLPQDNSNSNKTTEDNSTTASTATTEITNNNNRKCKGKGGPDNNKFRYRGVRQRSWGKWVAEIREPRKRTRKWLGTFATAEDAARAYDRAAIILYGSRAQLNLQPSGSSSQNSTRGSSSSASSSTSTSSTQTLRPLLPRPSGFGLTLPYPSSAHPVPLMASGFVPYGVDLGLGVYHNVAAAAAVAGCALTSSVRMNQHPHHMLDQDHEYQNNINPLHQQQQQHQQQQIVVQQFHHQYPIALSXGSGCDTSTSYLHPNPXHDQYQRQVPHQNNNNQECCSYXDVNSLVGSGLSTQPMEVAPGCSDIPVEAVGPMSPLMWPLTSEEECVPGLWDYGDPFFLDFKGLDS

>MDP0000242979 Malus domestica MCLLKVANQRSSGGGGQYDRFGSQNDSTDVTAVDIDNINYGGVGLESPPPYHQQHVSLSTPTMFLGYSQAATEMSAMVSALTHVVSGQRGSDSWGHVGSGGVTSSFGQLYSSSFPSASPLSAAFSSSASPGSHNWVGQKRGREEDLDSASASASAQTQFMESGNRAFRGGYSDYRGTQSESPSGGASATVTEESTNFSTATVSATTAVPTTPSSTESVSFEETGERKRRYRGVRQRPWGKWAAEIRDPHKAARVWLGTFDTAEAAARAYDEAALRFRGNRAKLNFPENVRLVQPPPPPPTLQTFNSNSRPTQYAQPLQPPPLPQPPQQQLYHSQPAFQPSSDLLRDYFDYSQLLQSSADFHPQQQQQQQPSSLLQKMYYNSQLASLQSSFLQPASASTPSSALPSSASSSASFPLFFSEQNQQMGFFRQPQNPNQGGPSDFQAPSWSHSGNNPSSSS

>MDP0000250924 Malus domestica MRKQNWNFAPLRLPHEQGSLVCLPPHISIGSVTGYGFSSSPPELNKKIAXKILNSSQKVLTMSTMEMSGGNSSAGVAEMSGGSSSVGVAEKKLAKRPAQGSSRKGCMRGKGGPENAMCTYKGVRQRTWGKWVAEIREPNRGARLWLGTFDTSYEAALAYDAAARKLYGSEAKLNLPDQQAPNYHHYQLNQHLQHQVFPSSANAGVPPQIPNQTLVQNNPSGTGTNPACYAPPVKDIAVPVVHNNFPNVKVDQPLGNYNSGGAEILPEQGGMKVENNMSSGVTNEGMDGIFWGNMSESFPVFDDSIWTEAAMSLDLPGVADQGIFGCGFMDGNFWEHRIRLNGA

>MDP0000250927 Malus domestica MSTSEMSVGVAEKKPAKRPAQGSSRKGCMKGKGGPENAMCTYKGVRQRTWGKWVAEIREPNRGARLWLGTFATSFEAALAYDAAARKLYGSKAKLNLPDQQAPNYHHHQLNQHLQHQVLGLILHVIYAAPVKDIAVPVIHNNFPNVKVDQPHGNYNSGGAETLPEQDGMKVEKNMSSGVNNEGMDGIFWGNMSESFPVFDDSMWTEAAMSLDLPVVADQGIFGGGSWMGIAGKHHIRLNGA

>MDP0000290585 Malus domestica MXSKMRGRDCYPQITVTRSEVTGNSLEAHGFAXKMGVLPPRRSRTXLIMGEARXRMRGIDMRVSTERDEEERGLGVGGGTEREKRVVSLNLKLSFHGALTIFIFQALLPPLQIPASILKGGPGTKTRSLIIIFPVQYNLNCMPKLVKTDXRRDGPNPVAETLAKWKEYNDHLDSCNDEGGPFRRVPAKGSKKGCMKGKGGPENSRCNYRGVRQRTWGKWVAEIRTPNRGSRLWLGTFPTAIEAALAYDEAARAMYXSAARLNFPNISISTLSKDSSSTKTLSXLPLLASSAGSESSARSDHSGDCAAQDEDQXNGLSSNVENDDTTNVDQTNEDRDDE

>MDP0000293996 Malus domestica MCTYKGVRQRTWGKWVVEIREPHRRARLWLGTFDTSYEAALAYDAAAAAAWKLYGTRTNPACYAAPVKYIAVPVICNNYANVKVDQPEXGGMKVEENMSSGVNNEGMDGIFWGKMXESFPVFDDSIWTXAAMSXDLPVVADQGIFGGGFTDGNCWETLPSSQWCMM

>MDP0000295902 Malus domestica MLKSEMNGGGSTSVXVEERKPSKWPAQASSRKGCMRGKGGPENAMCTYKGVRQRTWGKWVAEIREPNRGARLWLGTFDTSDEAALAYDVAARKLYGSVAKLNLPEQRVPHCHQHHLNQHLQNQVVPSPANPXVXPQIPNQTLVQNNPSGTGTNPAGYXAXVNDIAVPFIGDNFLXVKADQPHGNWAVALPEQGGTEVGENMSSGGNNEMEGILWGNTSGNFPVFDDSIWTEAXMSLDLPAVEDYGVFGGGFVDGTGWETMHSY

>MDP0000446783 Malus domestica MGAYDQGANMSSLPLDSSRKRKTRSRRDGNSVAETLEKWKEYNKKLESVNNEGKTRKVPAKGSKKGCMKGKGGPENARCNYRGVRQRTWGKWVAEIREPDRGSRLWLGTFPTALDAALAYDEAAKAMYGTGARLNLPHAANYHPSSSLETSSVATPSGSSAVATPGCSTSTSTSSRSEICGDEDSKLFLNVKKEDGEGESRMYPWSTAVPQASGMVKPEIIEDFTMDYLRNXQQQPVVKPEAGVEDHNWNGGEGFIGDYSENFTTDELFDMDEMFDVNELLMPSDDISLCNSGSEQVWRADVGQSGMETLSSERPSNLSYQLQFPDAKLLGSLQHMEHAPLNFEYGFDFKKQEKEGSNHTGQDDQGYFNLGLSDLDLEGFTGGITQSGDGGYNYSMEM

>MDP0000628484 Malus domestica MEEDQDRDSAAEAAPDHHLPQENSNKTTEDNSTTASTATTETTDNNNRKCKGKGGPENNKFRYRGVRQRSWGKWVAEIREPRKRTRKWLGTFSTAEDAARAYDRAAIILYGSRAQLNLQPSGSSSQNSTRGSSSSSSSSSSSTQTLRPLLPRPSGFGLTLPHLSSAHPVPLMASGFVPYGVEVGLGVYPNVATAAAVAGGGATSSVRLNQHPHHNNMLDQDHPQDNINPLHQQLQQQQQVVVQHFHHQYPISLSDRSSTSYQYPNSSHDQYQLQLHHQNNNNLECCSHDDVMNSLVGSGLSTEPMAVAPGCSDISMGGVGPISPSMWPLTSEEECVPSLWDYGDPFFLDLKGLDL

>MDP0000703626 Malus domestica MDNSRKSPLKPWKKGPTRGKGGPQNASCEYRGVRQRTWGKWVAEIREPKKRTRLWLGSFATAEEAAMAYDDAARRLYGPEAFLNLPHLQPSSNPSLKSQKFKWFPSHNFISMFPSCGLLNINAQPSVHVIHQRLQELKQNGVLGHTTPSSSSSSCDSKSEAHILSDKTEMRNVAEKEKDVEISSEKEAEDYQEKPQMDLNEFLQQLGVLNKETQSEATETTESFTAPEFSIGEVHDEFGPFADKNIXWDALIEMHGISNQGADAGTFQVYDMNEEPSFPTSIWNF

>MDP0000789227 Malus domestica MERGGKDKEEGQKDVKYRGVRTRPWGKFAAEIRDSTRQGARLWLGTFNTAEEAARAYDRAAFSMRGPLAILNFPIEYDLKDADQSAAAVSSSSSSLSSSSRPHNVTRTESGREIFEFECLDDSVLEDLLDFDNHTKTNEEKQIK

>MDP0000871361 Malus domestica MDNSRKSPLKPWKKGPTRGKGGPQNASCEYRGVRQRTWGKWVAEIREPKKRTRLWLGSFATAEEAAMAYDDAARRLYGPEAFLNLPHLQPSSNPSLKSQKFKWFPSHNFISMFPSCGLLNINAQPSVHVIHQRLQELKQNGVLGHTTPSSSSSSCDSKSEAHILSDKTEMRNVAEKEKDVEISSEKEAEDYQEKPQMDLNEFLQQLGVLNKETQSEATETTESFTAPEFSIGEVHDEFGPFADKNINWDALIEMHGISNQGADAGTFQVYDMNEEPSFPTSIWNF

>MDP0000880312 Malus domestica MASEASDGDRKLRKRRYGCDSIEDTLARWKNYNERLDFEKDGGKKTRRVPAKGSTKGCMRGKGGPENSACVYRGVRQRTWGKWVAEIREPIHATGGAVPKKKNNRLWLGTFPTAWEAALAYDKAAWAIYGASARLNFAKNTMDLKDSCSNFVSPTTTKPSSYESSSTYNSADQAKERSGFFEDCLPGEPKQEMVCAVGLVTEELELSNATLRDGCNPSNDVKFETPAMREAVVKEFPVILESGSQNGLDDMYNNMHNEQKNVQSDDAGMKGSITGQQLQGGLAETTKLNGHNHDGFIHTYAYLDDLDDKYNNSPGYGINASNDIGMQRELYDRLDYMHNCWSAEKTYGIDAAQEQQWERMMHCLPIQFQTQPQVDIPGSSNHTQDGYLDVDFDSDLVRQSYHSGVMEEQGLHYSWFPYS

>cassava4.1 008857m Manihot esculenta MGDAAKESSCSGSSSNSRKKLRRRRGGCESLEDTLDKWKKRNKLGEIRKPPSKGSRKGCMRGKGGPDNQSCRYRGVRQRVWGKWVAEIREPAGKFSLLNDARGHRRWLGTFATAIEAAQAYDSAAKAMYGSNAILNFPDYNSETESRTTSNSRDDNHIEKTKNHSCGEAESISRLKDEGSISRADYRSSTDLVKAEPMVKGTKEEFARVMESRGHYLQSEMKNVKAELSTDYECSNEIKEELGRSMESNQHDGLNNMHMPIYKDDDLRIDCKPFNDVEKLMMRKVMEGEGSSYCNPLEVRHDNMSIDFRDMNCYQELDLKCGINSALQAERTYDHDHFQLGSANYQQSNLAYQFQKQNPIADLLDCQNQTEEAKGSVDCSFNLWEPEFNWGSMEELGLMEPWDFGF

>cassava4.1 023912m Manihot esculenta MENCRKSPLKPWKKGPTRGKGGPQNAMCEYRGVRQRTWGKWVAEIREPKKRTRLWLGSFATAEEAAMAYDEAARRLYGPDAYLNLPHLHPSSIAPLINNKSHKFKWIPSKNFISMFPSRGLLNIHAQPSVHVIHQRLQELKKNGVFGQSSNVSSSSSSDSRNEVNIVNDKTHVENPPLMEKDMEITSEEMMGCHVHEEKPQIDLNEFLQQLGVLKVERQPENNDAPENFMENESPLKYYDEENNLAALEDKSFNWDSLIEMHGLADHHQAAETATLQVHDVQDDVSFPASIWNF

>cassava4.1 026885m Manihot esculenta MSKSITTPSLVEKKQLRKPAQASSRKGCMRGKGGPENALCTYKGVRQRTWGKWVAEIREPNRGARLWLGTFDTSHEAAMAYDAAARKLYGPDAKLNLPELHLNNNRFPAPSGHTQVSQVYMGNESQLLDDSDATCSSSNPIIRSSEMKSTYFRKSIMSFADENVEPDDKVAENEGNIDELWANLNVNLPLFDDSIWAEAAMSIDFPAIDNPGIIGGNLMEGTGWDALQTPWCM

>cassava4.1 028446m Manihot esculenta MENSRRSPLKPWKKGPARGKGGPQNAMCEYRGVRQRTWGKWVAEIREPKKRTRLWLGSFATAEEAAMAYDEAARRLYGPDAYLNLPHLQPSSINPLITSKSQKFKWIPSNNFIPMFPSCGLLNIHAQPSVHVIHQRLQELKKNGVLGQSSNVSNSSSSDSRNEANIVNDRTHVENHIVMEKDVEITSEKMVRYDEEKPQIDLNEFLQQLGILKVEGKPENNDATESCVEQVSLLNDDDKDNNLAAVADKSFNWDSLIEMHGIADHLTAESSSLQVHDVQEDPAFPIWNF

>cassava4.1 029110m Manihot esculenta MGTFDQASNATFRPLDSVRKRKRREGTNSVAETLQKWKEYNKYLDTCTPNGDGKPVRKAPAKGSRKGCMKGKGGPENSRCNYRGVRQRTWGKWVAEIREPNRGPRLWLGTFPTAYEAALAYDEAARAMHGALARVNFPESSNSTISSKDSHSAASYSSVATPAGSDSTTTSNHSEVCADEDSKEHIVKFGDDKGESKIKPVTEPASPCSTLKQEVKKELEDVSGSDCGGIPENEVPDKTVLQQYNTCVQKQKESCDLQHHKDEPLDVKDYGWDNGCEGQDYWKNFTIDEMFSVDELLGAIDSNPLGLDFDGGELFADDGHVQQEPPLDLSFQLQSPDDRFIGGRERVEQVPSVGDYNFDFLKPGRQEDENVPLDDQGCFSLELSNSGF

>cassava4.1 029523m Manihot esculenta MEQEAKDTNQSMRRSRKGCMKGKGGPENALCTYRGVRQRTWGKWVAEIREPNRGNRIWLGTFNTSHEAAKAYDQAALKLYGSSATLNLPQYCHTAAAAPANTLSTECQELGNGIETAASFSSQAVQELGNGAGTCCSSGEMGTAASFSSQPDSGSVEGGNIMHWPEFGIETEFLGSCDVGIATVGGEGLNWDGYPEQWSI

>cassava4.1 029526m Manihot esculenta MGPLGEKKQLRKPAQASSRKGCMRGKGGPENALCTYKGVRQRTWGKWVAEIREPNRGARLWLGTFDTSHEAAVAYDAAARKLYGPEAKLNLPELPLKNNQFSAPSGNTQVSQIQMGNESQVLHNSGATCSSSSSTLMKPSEMNHDSITPLSNENVASNSKVAENEGNLGQNQEGTDELWANLNVNLPLFDDSIWAEAAMSIDFPAMDDPGIFTGNLMDGTGWDAMQTPWCM

>cassava4.1 031773m Manihot esculenta MEEHPDPPFAPQETTTTTTTTPAAPSITTATTSDTNSDTNNNSSSDNSSRKCKGRGGPENGKFRYRGVRQRSWGKWVAEIREPRKRTRKWLGTFATAEDAARAYDRAAIILYGSRAQLNLQPSKSSSAQSSSSSSRGSSSSSAQTLRPLLPRPSGFGFTFSLSASMPSPAAAAAAAAAAATASASSGFGPYGVYHHHPNVVGSSVLCPSNMVQNPQEQIMSHHYQYHYQNPLIPDVSNLNAANSTIPTTSYQNLNYDYNDHNNHHHHHQQQQESGLFEDISSLMGSVGSNLSLSGNIQPVVAPAVQDPVMHVGPGSPSLWPLTSDDDYAPSTIWDYGDPSFFDL

>cassava4.1 033353m Manihot esculenta MDDSSLHHPPQTTTTTAPAAPSITIATTSDTNSDTSNNSSSDNSRKCKGRGGPDNSKFRYRGVRQRSWGKWVAEIREPRKRTRKWLGTFSTAEDAARAYDRAAIILYGSRAQLNLQPSNFSSAQSSSSSSRGSHSSSTQTLRPLLPRPSGFGFTFSLSASMPSPALTVAPAAASAASGLDPYGVFNHHHHPNVVGSGVLCPSNIVQNPQEQLMLHHQNPLPDVCNFGDSCSTLPITSYQNLNYDYDDHNRYHHQQQQQELGGGGLYEDISSLVGSVGSSLSLSSNTQPVIAPAGQDPVMHVGPGSPSVWPLAIDEYPPPSIWDYEDPSLFDL

>MEDTR1G028580.1 Medicago truncatula MMKSGMGIEERKQLKRPAQASSRKGCMRGKGGPENASCTYKGVRQRTWGKWVAEIREPNRGARLWLGTFETSHEAALAYDAAARKLYGSDAKLNLPELSTPPQNTTSSPSPTPPQMQQQQQHPHIQIQPNNNNNINNSFNICNNINMNNNNNNSPVFVSLSSQQVGGDITPIYSSDNSVMSFPLDSNSTITNTMESKGMEISSDSFFGTVNYETMPVIDDDSIWTEAAMSLDAAISMDFPMIVDDADGIYNSGANFAEVGAWDSLQTPWCM

>MEDTR2G103290.1 Medicago truncatula MLAKACEKGDGSKSLGKILARWREYNAQLDAGNDANKPVRKVAAKGSKKGCMKGKGGPENSRCNYRGVRQRTWGKWVSEIREPNRGSRLWLGTFTTAIGAALAYDEAARAMYGSRARLNFPNVSVTRFSDESPNDSPDANHLAVSTNTESMIIPDNSGIGVEDSNDMEPTSLCLSIKQENEEGESEIN

>MEDTR5G090830.1 Medicago truncatula MCGESLTKWKKYNAQLYAGKDDGIPKLKHLQKNKYRGVRQRTWGKWVAEIREPNSGSRLKLGTFPTAQEAALAYDSAARAMYGTSARLNLPDISDYSSVKEYLMDSSSAAASRSSSLATMPATSEKTTASSHSEVCVAEAVNEIPKLPVNMNNSVEIVEFGGGARCKFRRNNNTNTNT

>MEDTR5G090850.1 Medicago truncatula MGAPAYDQCFNVSLAPLELSKKRKTRSRGKGTKSVAETLAKWKEYNEKLYAGKDDGKPKRKAPAKGSKKGCMKGKGGPQNSENKYRGVRQRTWGKWVAEIREPNRGSRLWLGTFPTAQEAALAYDYAARAMYGPSARLNFPDISDYRSIQEYLKDSSAAAASCCSSVATTPATSETTTVSSHSEVCAIEDVKEIPRIPVHMNNTVDVCYKGYEATSPTSRMKQEPMDEPDDIIDLGGGEIQDANSEGTQTLTQPQTHDAVLVGEGVCNDQMDFSWMDNFDFDDDYLKSFSVDEFFHVDDFFEVDELLGQLDNNPIDDSGVMQSLDFGQVGLPEESNPQVGTTSSFFYELENPDAKLLGSLPHMERTASGVDYGLPLNFNGGGEDTPFLDLDYDLNHDSRGMQGRKND

>MEDTR7G076360.1 Medicago truncatula MKIDTVSGQRKSRKRRSGGRTDSVEDTLEKWKNYNRQQQQKLGCRGNGADKIHKVPAKGSRKGCMRGKGGPQNSDCNFRGVRQRIWGKWVAEIREPINGKHVGEKANRLWLGTFTTAHDAALAYDKAAKAMYGPSARLNFPDGSPSSSSGGSADSMNGEEDLGKAEELEGNLHQFEEENKILSKDFVSDDDSVEESKEVMIDGTVQCPTNKKCKKMVHQRSYKNVKSETHGENKRLERELGKVLENSSLDGEFNHVQKEPMDAGMNSGADRRSSDIADLVQSEETIRGSPEDLKSFELSCSNHFFGNQHNMLPDSNPRSSSEHCNIKTEASLAKKHKKEENGHFLSHARSQNEQNKNGYFDEMESELKGLEYKLGGQSIDCKNDEAQIVVPYMQGIHLFGGDSVGPIERMSQVEALNNNTNKNTKLKEKGSNGNAFHGLSSGQSRKLSDLSQQLQKLGGYLPENWNNMQFADLEVGYDYSFLKPDYDFGLLEEKKLLDICFSHIGS

>MEDTR7G110870.1 Medicago truncatula MDNSKKSPLKPWKKGPTRGKGGPQNASCEYRGVRQRTWGKWVAEIREPKKRTRLWLGSFATAEEAAMAYDEAARRLYGPDAYLNLPHMQTHSNSTMKTGKFKWLPSKNFISMFPSCGLLNVNAQPSVHLIHQRLQEFKQNAVVASQSSFSSSSNDPKAEEIQKVDSKKSHTEDPLPKETIVQTSANKMLGDLQEEKPQIDLNEFLQQMGILKEGSHSEQTESSGSSTVHEVLPRDDNDQLGIFSDMSVNWEALIEMHEFAGIEESEATHLEAYDPNDHLNFSTSIWDF

>mgv1a010327m Mimulus guttatus MTMLNQDSNTAYQPIDCTRKRKSRSRKEGATSVADILEKWKKYNTKIDSVDNRSKAIRKAPAKGSKKGCMKGKGGPENSCCNYRGVRQRTWGKWVSEIREPHRGGRLWLGTFGTGFEAALAYDEAARIMYGPCARLNFPGYSRDSSVLPAVSSDSSGVSEICCDDVGQRVDISVMKAEESNVGPATPMSEVKKEVVEELPREKVKEEVEQPTSSNGDGEISHVENCGQDQFEKFELDEMFDVEELLASLDSAPAPQVGAGNRAALNVPPSSQPQSGDAQGLSGHDNDFDFLMPGRQEDSNFLLSDLFMDLDSDLAI

>mgv1a011288m Mimulus guttatus MENCRKSPLKPWKKGPARGKGGPQNATCEYRGVRQRTWGKWVAEIREPKKRARLWLGSFATAEEAAMAYDEAARRLYGPEAYLNLPHLRSNFNPLNKSHKFKWFPSNSNSNSNNLVSMFPTTGLLNLNAQPSVHVIHQRLQELKKTGVFGLDSSSSSSSAPKKDVQSVEILEKEKKDNEITSGVKAVTSEEKPQIDLNEFLQQLGVLKRDEHQTCVSDVSKSFTEIESLSSFRDDEDGFCNYAEKNFNWDTLSEIGGADIHVGAESSTYQVHDSNEELVFSPSIWNF

>mgv1a018624m Mimulus guttatus MAAAAAAPAEEKKVRKLAQASSRKGCMRGKGGPENAACTYKGVRQRTWGKWVAEIREPNRGSRVWLGTFDTSHEAAVAYDAAARKLYGPDAKVNLPHLCDTAAATAAANNQTPALAQAAAPAKVENQECYNYNYNYNYNPNTNLTSGTANFNNGGGGGGEYVSASGILKKLNVSLPDVDDSHLWAEAAKDTSFQMVHEQEHCGKDGNGLPFTWLY

>mgv1a018752m Mimulus guttatus MATHYHQTTVLIPYDDGGGGSSSGNPPPPPKTKPPSVRRSRKGCMRGKGGPENALCTYRGVRQRTWGRWVAEIREPNRGARVWLGTFSTSLEAAQAYDDAARRLYGPFAKLNL

>mgv1a024901m Mimulus guttatus MSGAAISDPNIHLPRDHDEQNNDYNPIIPSLSTTTTSSDTASGGNNTTTPTTNGAKKGKGGAAGGKGGPDNGKFRYRGVRQRSWGKWVAEIREPRKRTRRWLGTFATAEDAARAYDRAAIILYGNKAQLNLQPSGSGGASNGGGVSGGSSSHSSSRTTTSSSSSSTQTLRPLLPRPAGFGLTFSAPPPQIPPPALVGMAANYPQYGLYPSTVQYPNNNIVPQQQMCLVQQQQQQYGSNNDFKIIEADPTIVRDQSSKYSSYPNPNPNPNPNPNNNNTDYYPQQIPDFNHLQSNVGYDEMSSYVGSVGSSLSNNAKTDHLPAAPAITDPAEVLNGYVTSPTWPLASEDDYPSTSLWDYGDPFLFDNI

>GSMUA Achr11P24820 001 Musa acuminata MDGEKPIRKPPARGSKKGCMRGKGGPENPSCRYRGVRQRTWGKWVAEIREPNRGGRLWLGTFPTATEAALAYDDAARAMYGSLARVNLPGAVMSKSCESTTTSHHSDAINASVSSTGHIKVPSIEPKDKVHFPKAELDDEELKGEAGCDKEPSSTADASNMGVCQYGDQTDAPEDEFSVEEMLRLMGDDTEVNVHDQFGTVHADTNWQCFSPSGMSSGFQNPDATTLGSLWRTEENPFDCKDSLLWPWVDDQDKGPEEAIETSEFGVSYGDFLSSPNIW

>GSMUA Achr3P00320 001 Musa acuminata MGPEEKKQKKCCPLRRSRKGCMKGKGGPENQACTYRGVRQRTWGKWVAEIREPNRGARLWLGTFSSSLEAAQAYDNAARSLYGDCARLNLSDDSGPRRPSVKSEASCSPTCSTETSTSESRATFPGSVQSPAGYFCGGGGGLDDFDDYVTGLPKAEDFGLEAFSDIPLFDDVGFAETMFDKELLSFDAMQLSWCS

>GSMUA Achr5P28050 001 Musa acuminata MRGKGGPENRSCRYRGVRQRTWGKWVAEIREPNRGNRLWLGTFPTAVQAALAYDDAARAMYGTSARINLPGVVRRDSSESTTTLHHSDAIGASVSSADQIKFLITKLKDEAHSDRMNVSGSRESIIEFPTVELEDEGGRNEEPSFVADACNVELYQSDAPEGEFSIEDMLRIMGADTDNSDADHFGAVGRETNWQCTDPVDMSLDVRHTDAPTVWDIEQNPFDYSDTLLRSLGEDWEYGPGETPNTVDFGISCADLLSSPERWYKS

>GSMUA Achr6P32780 001 Musa acuminata MKVYVYGQIHNPSLRDQAIGHAIAERSSDTCQTRRLSFRHVLSHVTRLLLYKDLGTAFGISWRPRNIWKPRRKRSRALNQEEARQMGVMTMVESDRRSRVRRGRNGSSSVAETIAWWRERNRQLKCSVDTEKRVRRPPAKGSKKGCMRGKGGPENPNCRYRGVRQRTWGKWVAEIREPNRGSRLWLGTFPTAFQAALAYDDAARAMYGTMARLNLPAMTRGLCGSTSTSYNSEDVDASVSCDFGIKTPRVDAGSETVDAPRTWADNIKHPKIELKEDVASDMWPSSAADAPETQSTEASVDLLLCQSPREDEFSVEEMLRMMGTDTDNGAPDQFGAVAADTGWQCMIPADVGLDLQSPDGTVLGPLWGTEQNPPLEFDCCDSLQRPLGDDWEYGPGEFGMLDADILSSPIN

>GSMUA Achr9P04630 001 Musa acuminata MDGEERVRRAPAKGSRKGCMRGKGGPENPKCRYRGVRQRTWGKWVAEIREPNRGSRLWLGTFPTAVEAALAYDDAARAMYGTMARVNLPGAADVPAMRRESCETTTTSRKSSAVDGSISREIDRKVPRFDPRDEVAYSDATDVSDDWDKDVKLLPKVQQKDDAEKESTTTEACVELRQTGDRDPPTTDGREEEFSVEEMLRMMGADAEASMPCHLGPVGGDVNWQGISAADDLALLDLQQNPDATLWGMAQNPLGFDYGDDSLWRPLGDDWEYGPVEVPKRAELGTFDADFSSGPRR

>GSMUA AchrUn randomP08690 001 Musa acuminata MGPQEKQPRKCCPLRRSRKGCMKGKGGPENQACTYRGVRQRTWGKWVAEIREPNRGARLWLGTFSTALEAAQAYDNAARSLYGDCARLNLSDSRPSSVGDNSTGSTPPPPAAALVKPDFRCSSSWSNETSTGDSQASFPDSIGLPAAHFYAGGLYSFDDYVSGLPKPEDFGLEAFHSVPFLDEQELDTQFMNFEPLQVSSYS

>GSMUA AchrUn randomP14580 001 Musa acuminata MAMEQERKKRQPRRGHDGPNSVAETIARWREHNGQLDADNCVRRAPAKGSKKGCMKGKGGPDNPNCQYRGVRQRTWGKWVAEIREPNRGNRLWLGTFPTALEAAVAYDEAARAMYGPYARLNLPERYGDSQLGATNGSCESCMTSHQSHSDVGISSSGETEVIPKIEKTDERDNGLSSSGNHKIEHSQPGYWNEDFPIDEIFLEALRLPAARVGITCKDHRGRVVLYRAATADDNGYVFAELYTTTMRGGYFDPAESCAVRLLVSPDDRCSSVTDVNGGDRGTPLRYQNTTIPGQYADLDVYVAGPLAFKPAYCPPKTT

>NNU 005476-RA Nelumbo nucifera MEPTLPTYRGVRKRAWGKWVAEIREPNRGSRLWLGTFSNALEASLAYNEATKAMYGRCVRLNFPESSASMESSYLATTSSSYSWRTELILRE

>NNU 005977-RA Nelumbo nucifera MVEINICQDNSITVQEQEPEKKRKASLYCMSSLITEKKRKSRRRRKGCDSIAERLAKWKEFNEKHDTAGDGAKRNRKAPAKGSRKGCMRGKGGPENLRCNYRGVRQRTWGKWVAEIREPNRGSRLWLGTFSTALEAALAYDKAAKAMYGRYARLNFPESSASMESSYSATTSSSYSEVCLAEELKVNLPKVEPRPEQCESEIYMEAVAPAPMNIVKQEPKEESVEPMNPDQFRGYGITQGPIDSGHLTQFSESGCNRFDDVQSFSLEELFDMEEPRMKNEYDQLGGSDQLLCGSPLDLSYQLLNPIELEGDDGYGLYEYDEQRLLHLGFPDFLWF

>NNU 007747-RA Nelumbo nucifera MGACELVFNSTSLPLNHGRKRKSRSRRNGCESIAERLSKWKEFNDQLDVAGDEGKLIRKVPAKGSKKGCMRGKGGPENSRCNYRGVRQRTWGKWVAEIREPNRGSRLWLGTFATALEAARAYDEAARAMYGPCARLNLPECSTSKDSSYSATTPSTSGSTTTPSTSDSTTTSNHSEVCLSEESKANVPKVEPKRELCEFGINSRPHIAMEADAPMSIVKKESKEEESVQPMNPDLLRGYETSQDPVRSGQFNGNGCGQEDMQNFPTEELFDMEELLGMLDNDTPGPELQQELGVVSDQLQCGSPSDLSYQLQNPDAKLLGSLYHMEQSPASMDYSYDFLKPAEQDPMKQEGDYSYGLYGEQGSLELGFPDLGF

>NNU 007903-RA Nelumbo nucifera MESCRKGPLKPWKKGPTRGKGGPQNASCEYRGVRQRTWGKWVAEIREPKKRTRLWLGSFSTAEEAAMAYDEAARRLYGPEAYLNLPHLQPNSSPNKPNQFKWFPSKNFISMFPSCDLLNINAQHNVHAIHQRLQELKRNSSSLNQLPPSSSSSSSFDSNSEFFITEDKCYIENSLAKQEEAMLSSEQLLGGREEKPQIDLHEFLQQLGILKEGSGSEGGDTLGSFPLPESLLEDDGLTAFGEQTFNWGSLVDIHGLEGNQGAVNSGIQVDIHEELTFPSSIWNF

>NNU 012883-RA Nelumbo nucifera MESYRKGPLKPWKKGPARGKGGPQNASCEYRGVRQRTWGKWVAEIREPKKRTRLWLGSFATAEEAAMAYDEAARRLYGPEAYLNLPHLQPSNCFNPPDKSQKFKWFPSKNFLSMFPSCGLLNVNAQHTVHVIHQRLQELKWNSSTLNQLPSSSSSTFDMKSELWITDDKSYVENSVVNQEEVVLSSENPVLVSHEEKPQLDLNEFLQKLGILKEERRLDSSDTPESFPLPEYLPEDDEQAAFGEQTFNWDSLVEIHGLGGHQGAVDNGLQVDIHEELTFPSSIWNF

>NNU 016649-RA Nelumbo nucifera MRGKGGPENALCTYRGVRQRTWGKWVAEIREPNRGARLWLGTFDTSRDAAIAYDSAARKLYGKFAKLNLPELWTEAESQAGSISESVPVPVPSSTTTTQQAAESLLKVEPEMGSSSDGNNLVEDGMEGIWRDMNLSLLPEIQEFPWMELPTTTMEFPVVMKDPGIWSQELLDDTTGWNALELPW

>NNU 023626-RA Nelumbo nucifera MGKGEKRSPKRNSRKGCMRGKGGPENALCPYRGVRQRTWGKWVAEIREPNRGARIWLGTFNTSLEAAVAYDDAARKLYGSSARLNLPELATSPASCSPAISLASGSTTTSSSSSSFPGLDTSTSDSSKSFPASTDTEFTINDLESQFTVEDLMKDNDERLMPNSNSDMEGGFWGNMALELPEWLEAPPVDSDFLTIGDILGWDS

>ObartAA03S FGP0424 Oryza barthii MERGEGRRGDCSVQLCGIIELPHWVRKKRTRRKSDGPDSIAETIKWWKEQNQKLQEENSSRKAPAKGSKKGCMAGKGGPENSNCAYRGVRQRTWGKWVAEIREPNRGRRLWLGSFPTALEAAHAYDEAARAMYGPTARVNFADNSTDANSGCTSAPSLMMSNGPATIPSDEKDELESPPFIMANGPAVLYRPDKKDVLERVVPEVQDVKTEGSNGLKRVCQEQKTMEVCESEGIVLHKEVNISYDYFNVHEVVEMIIVELSADQKTEVHEEYQEGDDGFSLFSY

>ObartAA03S FGP21558 Oryza barthii MTVDQRTTAKAIMPPVEMPPVQPGRKKRPRRSRDGPTSVAETIKRWAELNNQQELDPQGPKKARKAPAKGSKKGCMKGKGGPENTRCDFRGVRQRTWGKWVAEIREPNRQSRLWLGTFPTAEAAACAYDEAARAMYGAMARTNFGQHHAPAASVQVAQAAVKCALPGGGLTASKSRTSTQGASADVQDVLTGCLSACESTTTTINNQSDVVSTLHKPEEVSEISSPLRAPPAVLEDGSNEDKAESVTYDENIVSQQRAPPEAEASNGRGEEVFEPLEPIASLPEDQGDYCFDIDEMLRMMEADPTNEGLWKGDKDGSDAILELGQDEPFYYEGVDPGMLDNLLRSDEPAWLSADPAMFISGGFEDDSQFFEGL

>ObartAA03S FGP22193 Oryza barthii MAAGEGDVGMEVETKAPAMPPPPPASSSAARKKKQARAKNGDTPEPDAAGGARARASRRAKRGPGSYRGVRQRRWGKWVSEIREPNRGKRHWLGTFGSAVDAALAYDKAAASILGPRAVLNFPAFSPPAAAIAAPEQREPPFCSPATTAAATAPEQRQTPGCSPAAVAGSGGGAVFEESDVKPVVLPLPLPAILQGGGGTEAMAQHWDWEWDASWPELEMFECLDDIAMYLDVDAVMTTRDCKVEELDADIVDSLLWTLSD

>OB01G13930.1 Oryza brachyantha MERGEGRKGDCSAQVRKKRTRRKSDGPDSIAETIKWWKEQNEKLQEESSSRKAPAKGSKKGCMAGKGGPENSNCAYRGVRQRTWGKWVAEIREPNRGRRLWLGSFPTALEAAHAYDEAARAMYGPTARVNFADNCADTNSGCTSAPSLMMSNGPTTVRSDEKDELESPPFVVVNGPTVVLHRSGKKDALECVIPDQQLKKEVSNDLRSTCEEQETMDACQSEGSVLHKEVNVSYDYFNVHEVVEMIIVELSADQKMEVHEEYQEGDDGFSLFSY

>OB05G20700.1 Oryza brachyantha MTVDRKTAIMPPPVEMPPVQPGRKKRPRRSRDGPTSVAETIKRWAELNKQLEHGSQGPKQARKAPAKGSKKGCMKGKGGPENTHCDFRGVRQRTWGKWVAEIREPNRLSRLWLGTFPTAEAAARAYDDAARAMYGPMARTNFPRQHVPAASAQVALATVKGVLPSGLSACESGTSTHVAPAAVQDVLPSGLSACESTTTSNNHSDVASTLHKPEFSEISSPLRAAPAVLVDGSLYEDKVGSITYDENIVSQQCAPPEAETSSGRGEEVFEPLEPIASLPEDQEDCFDIDEMLKMMEDDPMNKGSWQPNGEGLTNGGGVDPMGMGEIGQDEPLYLEGVDPAMLENMLKSFDSGPAWLSEDPAMFISGGFEDAEFFQGF

>OB05G27530.1 Oryza brachyantha MAGGEREAAAVGMTSEGVEVEKEPATLLPSPSVPRKKQTHAKNSDTPDAGGARRAPRAKRCPNSYRGVRQRRWGKWVSEIREPNRGKRHWLGTFDSAADAAPAHDKAAAAILGNRAVLNFPASSPLAAAVAPEQREAPCCSSAAAVPAAVFEEEHAVKPAVLPLMQGGAGGTETKARHWEWDAASWPAQGMFQCLDDIAMYLELDAVKTEDCQVEQLDDDVFDSPLWSLL

>OB08G30790.1 Oryza brachyantha MENGESCCGRRKQQEAKTRKCCPLRRSRKGCMKGKGGPENQRCPFRGVRQRTWGKWVAEIREPNRGARLWLGTFNTALDAARAYDSAARALYGDCARLNLLVAAAGHTSTQQQQHMISSSAATTTTTIIGSKHESCSSNDSNSPSPMPMLLADYGGVMMHPAEEEEDFETYVTRLPKAEDFGLEGFQEVPLDVLDEAGGGISIWDLSICPADIMAAAATAK

>ORGLA01G0037700.1 Oryza glaberrima MLFRFVSCNVQLCGIIELPHWVRKKRTRRKSDGPDSIAETIKWWKEQNQKLQEENSSRKAPAKGSKKGCMAGKGGPENSNCAYRGVRQRTWGKWVAEIREPNRGRRLWLGSFPTALEAAHAYDEAARAMYGPTARVNFADNSTDANSGCTSAPSLMMSNGPATIPSDEKDELESPPFIMANGPAVLYRPDKKDVLERVVPEVQDVKTEGSNGLKRVCQEQKTMEVCESEGIVLHKEVNISYDYFNVHEVVEMIIVELSADQKTEVHEEYQEGDDGFSLFSY

>ORGLA05G0107600.1 Oryza glaberrima MTVDQRTTAKAIMPPVEMPPVQPGRKKRPRRSRDGPTSVAETIKRWAELNNQQELDPQGPKKARKAPAKGSKKGCMKGKGGPENTRCDFRGVRQRTWGKWVAEIREPNRQSRLWLGTFPTAEAAACAYDEAARAMYGAMARTNFGQHHAPAASVQVAQAAVKCALPGGGLTASKSRTSTQGASADVQDVLTGCLSACESTTTTINNQSDVVSTLHKPEEVSEISSPLRAPPAVLEDGSNEDKAESVTYDENIVSQQRAPPEAEASNGRGEEVFEPLEPIASLPEDQGDYCFDIDEMLRMMEADPTNEGLWKGDKDGSDAILELGQDEPFYYEGVDPGMLDNLLRSDEPAWLSADPAMFISGGFEDDSQFFEGL

>ORGLA05G0109500.1 Oryza glaberrima MEPSDDAFTVAAPAAETAASSSGAGGGGGGGRTKKKAAGKGGPENGKFRYRGVRQRSWGKWVAEIREPRKRSRKWLGTFATAEDAARAYDRAALLLYGPRAHLNLTAPPPLPPPPPSSAAAAAASSSSAASSTSAPPPPPLRPLLPRPPHLHPAFHHQPFHHHLLQPQPPPPPPPPPLYYAATASTSTVTTTTTAPPPQLAAAAPAAVLVAAAVSSTAETQAVVATAPEDAASAAAAAAAEEEAAWGFHGGDEEDYAAALLWSEPDPWFDLFLK

>ORGLA08G0193700.1 Oryza glaberrima MEMDIGEGESCCGRRKQQQQQNISSSKSRKCCPLRRSRKGCMKGKGGPENQRCPFRGVRQRTWGKWVAEIREPNRGARLWLGTFNTALDAARAYDSAARALYGDCARLNLLLAAATAGAPPAAAATPSVATPCSTNDDSNNSSSTTHQQQLTTMLQLDDDNYTLQPSSSDQEDFETYVTRLPKAEDFGLEGFQEVPLDVLDEAGGGISIWDLSICPADFMAAAAATTAKSS

>OpuncBB FGP0408 Oryza punctata MERGEGRKGDCSAQFCEIIELPYWARKKRTRRKSDGPDSIAETIKWWKEQNQKLQEENSSRKAPAKGSKKGCMAGKGGPENSNCAYRGVRQRTWGKWVAEIREPNRGRRLWLGSFPTALEAAHAYDEAARAMYGPTARVNFADNSTDANSGCTSAPSLMMSNGPTTIHSDEKDELESPPFMVTNGPAVLYRSDKRDVLERVVPEVQDVKTEGSDDLKSVCQERKAMEVCESEGIVLHKEVNVSYDYFNVHEVVEMIIVELSADQKTEVHEEYQEGDDGFSLFSY

>OpuncBB FGP14026 Oryza punctata MESYGRKRAWKKGPTRGKGGPQNAACEYRGVRQRTWGKWVAEIREPNKRTRLWLGSFATAEEAALAYDEAARRLYGPDAFLNLPHLRAASAAAAHQRLRWLPASAAAGRGGAAAVPAYGLLNLNAQHNVHVIHQRLQELKNSSSPTKSPRTPARADLPPPLPTSSPCSTVTNSVGSAALPPPMSCFQALEQAMAMESAPCDDAAVVGFGADKPQLDLKEFLQQIGVLKADDDGATGKNGAVHGDDGELADAFGFGGNGEFDWDALTADMSDIAGGHGGALGANGGFQMDDLHEVEQFGGCMPIPIWDI

>OpuncBB FGP21124 Oryza punctata MTVNQTATAIMPPVEMPPVQPGRKKRPRRSRDGPTSVAETIKRWAELNNQQEYDPQGPKKARKAPAKGSKKGCMKGKGGPENTHCDFRGVRQRTWGKWVAEIREPNRQSRLWLGTFPTAEAAACAYDEAARAMYGPMARTNFSQQHAPAASVQVAQAAVKGALPGGGLSACESRTSIQVAPAAVQDVLTGGVSACESTTTTINNQSDVVSTLHKPEEVTEISSPLRAPPAILEDDGNYEDKAESVTYDENIVSQQHAPPEAETSNGRSEEVFEPLEPIASLPEDQGDYCFDIDEMLRMMEADPTNEGSWKGSRDGSDAMLELGEDEPFYFEGVDPGMLDNLLRSDEPAWLSADPAMFISGGFEDDSQFFEGL

>OpuncBB FGP21149 Oryza punctata MQPSVDAATAAVAAPAETASSSGGGGRSTAKKAAGKGGPENGKFRYRGVRQRSWGKWVAEIREPRKRSRKWLGTFATAEDAARAYDRAALLLYGPRAHLNLTAPPPLPPAPSSDSAAAAASSSSAASSTSAAPPLRPLLPRPPHLHPAFHHQQFHHHLLQQQPPPPPPLYYATTASTSTVTTTTTTTTAPPPQLTAAAPAVLVAAAVSSTAETQAVAAPEDAAAAAAAEVAWGYHGGDEEDYAAALLWSEPDPWFDLFLK

>OpuncBB FGP21809 Oryza punctata MTAGEGDVGMKREGMEVEKAPVAAASSAGRKKKQARAKNGATPDAAGGARGRAARRAKRGPGSYRGVRQRPWGKWVSEFREPNYGKRHWLGTFGSAVDAALAYDKAAVAILGPRAVLNFPAAITPSAAIAAPEQRETPCCSPAAAAAVFEERDVKPVVLPMPAILQVGGVEAMARPWDWEWGASWPEQEMFECLDDIAVYLDVDAVMKTRDCKVEELNADIVDSPLWTLSD

>OpuncBB FGP29907 Oryza punctata MERSMEASLNNSGESCCGRRKQQQTNNISNKGSSSRKCCPLRRSRKGCMKGKGGPENQRCPFRGVRQRTWGKWVAEIREPNRGARLWLGTFNTALDAARAYDSAARALYGDCARLNLLLAAAGAGTSATPPAAVPTPSTPCSGNDSNSSSTQQQQHPLTMLLDDDNYTLQQMPSSEEDFETYVTRLPKAEDFGLEGFQEVPLDVLDETGGGISIWDLSICPSDVMAAAATATAK

>BGIOSGA002846-PA Oryza sativa subsp. indica MERGEGRRGDCSVQLCGIIELPHWVRKKRTRRKSDGPDSIAETIKWWKEQNQKLQEENSSRKAPAKGSKKGCMAGKGGPENSNCAYRGVRQRTWGKWVAEIREPNRGRRLWLGSFPTALEAAHAYDEAARAMYGPTARVNFADNSTDANSGCTSAPSLMMSNGPATIPSDEKDELESPPFIVANGPAVLYQPDKKDVLERVVPEVQDVKTEGSNGLKRVCQERKTMEVCESEGIVLHKEVNISYDYFNVHEVVEMIIVELSADQKTEVHEEYQEGDDGFSLFSY

>BGIOSGA011963-PA Oryza sativa subsp. indica MESYGRKRAWKKGPTRGKGGPQNAACEYRGVRQRTWGKWVAEIREPNKRTRLWLGSFATAEEAALAYDEAARRLYGPDAFLNLPHLRAASAAAAHQRLRWLPASAAARGGAAAVPAYGLLNLNAQHNVHVIHQRLQELKNSSSSPTKPPPRTPTRANPPPPPLPTSSPCSTVTNSVGSAALPPPMSCFQALEQAMAATAAMESAPCDDDAAVVGFGADKPQLDLKEFLQQIGVLKADDDGATGKNGAVHGDDGELADAFGFGGSGEFDWDALAADMSDIAGGHGGALGANGGFQMDDLHEVEQFGGCMPIPIWDI

>BGIOSGA018253-PA Oryza sativa subsp. indica MTVDQRTTAKAIMPPVEMPPVQPGRKCGGEESTGNLDSVQPIAGALPCNEHALLAQQTPKGDAPSVGSKIWKKRPRRSRDGPTSVAETIKRWAELNNQQELDPQGPKKARKAPAKGSKKGCMKGKGGPENTRCDFRGVRQRTWGKWVAEIREPNRQSRLWLGTFPTAEAAACAYDEAARAMYGPMARTNFGQHHAPAASVQVVAQAAVKCALPGGGLTASKSRTSTQGASADVQDVLTGGLSACESTTTTINNQSDVVSTLHKPEEVSEISSPLRAPPAVLEDGSNEDKAESVTYDENIVSQQRAPPEAEASNGRGEEVFEPLEPIASLPEDQGDYCFDIDEMLRMMEADPTNEGLWKGDKDGSDAILELGQDEPFYYEGVDPGMLDNLLRSDEPAWLSADPAMFISGGFEDDSQFFEGL

>BGIOSGA020033-PA Oryza sativa subsp. indica MAAGEGDVGMEVETKAPAMPPPPPASSSAARKKKQARAKNGDTPEPDAAGGARARASRRAKRGPGSYRGVRQRRWGKWVSEIREPNRGKRHWLGTFGSAVDAALAYDKAAASILGPRAVLNFPAFSPPAAAIAAPEQCEPPFCSPATTAAATAPEQRQTPGCSPAAVAGSGGGAVFEERDVKPVVLPLPLPAILQDGGGTEAMAQHWDWEWDASWPELEMFECLDDIAMYLDVDAVMTTRDCKVEELDADIVDSPLWTLSD

>BGIOSGA026461-PA Oryza sativa subsp. indica MEMDIGEGESCCGRRKQQQQQNISSSKSRKCCPLRRSRKGCMKGKGGPENQRCPFRGVRQRTWGKWVAEIREPNRGARLWLGTFNTALDAARAYDSAARALYGDCARLNLLLAAATAGAPPAAATPSVATPCSTNDDSNNSSSTTHQQQLTTMLQLDDDNYTLQPSSSDQEDFETYVTRLPKAEDFGLEGFQEVPLDVLDEAGGGISIWDLSICPADFMATAATTTAKSS

>LOC Os01g07120.1 Oryza sativa subsp. japonica MLFRFVSCNVQLCGIIELPHWVRKKRTRRKSDGPDSIAETIKWWKEQNQKLQEENSSRKAPAKGSKKGCMAGKGGPENSNCAYRGVRQRTWGKWVAEIREPNRGRRLWLGSFPTALEAAHAYDEAARAMYGPTARVNFADNSTDANSGCTSAPSLMMSNGPATIPSDEKDELESPPFIVANGPAVLYQPDKKDVLERVVPEVQDVKTEGSNGLKRVCQERKNMEVCESEGIVLHKEVNISYDYFNVHEVVEMIIVELSADQKTEVHEEYQEGDDGFSLFSY

>LOC Os03g07830.1 Oryza sativa subsp. japonica MESYGRKRAWKKGPTRGKGGPQNAACEYRGVRQRTWGKWVAEIREPNKRTRLWLGSFATAEEAALAYDEAARRLYGPDAFLNLPHLRAASAAAAHQRLRWLPASAAAAAARGGAAAVPAYGLLNLNAQHNVHVIHQRLQELKNSSSSPTKPPPRTPTRANPPPPPLPTSSPCSTVTNSVGSAALPPPMSCFQALEQAMAATAAMESAPCDDDAAVVGFGADKPQLDLKEFLQQIGVLKADDDGATGKNGAVHGDDGELADAFGFGGSGEFDWDALAADMSDIAGGHGGALGANGGFQMDDLHEVEQFGGCMPIPIWDI

>LOC Os05g27930.1 Oryza sativa subsp. japonica MTVDQRTTAKAIMPPVEMPPVQPGRKKRPRRSRDGPTSVAETIKRWAELNNQQELDPQGPKKARKAPAKGSKKGCMKGKGGPENTRCDFRGVRQRTWGKWVAEIREPNQQSRLWLGTFPTAEAAACAYDEAARAMYGPMARTNFGQHHAPAASVQVALAAVKCALPGGGLTASKSRTSTQGASADVQDVLTGGLSACESTTTTINNQSDVVSTLHKPEEVSEISSPLRAPPAVLEDGSNEDKAESVTYDENIVSQQRAPPEAEASNGRGEEVFEPLEPIASLPEDQGDYCFDIDEMLRMMEADPTNEGLWKGDKDGSDAILELGQDEPFYYEGVDPGMLDNLLRSDEPAWLLADPAMFISGGFEDDSQFFEGL

>LOC Os05g39590.1 Oryza sativa subsp. japonica MAAGEGDVGMEVETKAPAMPPPPPASSSAARKKKQARAKNGDTPEPDAAGGARARASRRAKRGPGSYRGVRQRRWGKWVSEIREPNRGKRHWLGTFGSAVDAALAYDKAAASILGPRAVLNFPAFSPPAAAIAAPEQCEPPFCSPATTAAATAPEQRQTPGCSPAAVAGSGGGAVFEERDVKPVVLPLPLPAILQDGGGTEAMAQHWDWEWDASWPELEMFECLDDIAMYLDVDAVMTTRDCKVEELDADIVDSPLWTLSD

>LOC Os08g45110.1 Oryza sativa subsp. japonica MILIHRYNQASMEMDIGEGESCCGRRKQQQQQNISSSKSRKCCPLRRSRKGCMKGKGGPENQRCPFRGVRQRTWGKWVAEIREPNRGARLWLGTFNTALDAARAYDSAARALYGDCARLNLLLAAATAGAPPAAATPSVATPCSTNDDSNNSSSTTHQQQLTTMLQLDDDNYTLQPSSSDQEDFETYVTRLPKAEDFGLEGFQEVPLDVLDEAGGGISIWDLSICPADFMATAATTTAKSS

>PDK 30s1022981g004 Phoenix dactylifera MAEPERTFVMRFDMGTCLYFDSKIDFSFPKHSKSCAIEVVINEIILLHMILGMSADLERLIVQRHADEKSRTNFKNKVVCVKVKESFQDRRRSWNRCAQTIKKNLAFCNQNQVVVIVSLNQGTSFPTNAEVLVLYARKKRVRRSHNGPNSVAETIARWREQNSQLECSFNGEKRARRAPAKGSKKGCMKGKGGPDNAHCKYRGVRQRTWGKWVAEIREPNRGSRLWLGTFPTALEAALAYDEAAKAMYGPYARLNLPQFNGAANAQATTSGSCESTTTSHHSDVSHDWSPRSPEVKVPKKELEEEIRNDSLPPAPVGAAAKVEQPGEELFGPRDQLEDLPEDMFDIGDMLRMMDADPTNRGVGGEHTDGRTGHSGDVDADINWQLSSPSALSFQLQNPDAKLLGSLHHMEQNPIAMDYGYDDFVSQLEQDWKYGLPDDDPGMWEMGYPESDLF

>PDK 30s667421g004 Phoenix dactylifera METITNLLGSRKGYIRGLKFEAVRREAREGGKQSSDVDKYARRVPAKGSRKGCMQGKGGPENSHCNYRGVRQRTWGKWVAEIREPNRGSRLWLGTFNTALDAAMAYDEAARAMYGPCARLNLPECGIAAKDTTLANAASYESTTTMSQHSNVSGVEESGVEAPKLEAGDETRSVNPPQSTAEQPVTSMAKTEADEELFKHFDSLQDLPQDMFGIEDILGNMDSWEEPGNLVSNGANTG

>PDK 30s699491g008 Phoenix dactylifera TKRVRNLHGGTHPVAEAVAKWRKLKWQPDCSNDGEKYIQRVPAKGSRKGCMRGKGGPENSHCNYRGVRQRTWGKWVAEIRKPNGGSRLWLGTFNTALEAALAYDEAARAMYGPRARLNLPECGTAAKDTTLAASECYESAPTTSQHSGVSGVEESEVKAPKLEAGAETRSANPPESAVEAVACMVKAEADEELFNHLDRLQDLHQDMFGIGEMMGNADADPSNSTSTPQSRKSQKDQLGGVDVITDWHFGETPSALSFQIQSPDVKRLGTWCHVEQCPADMDYGYGFAGPRRQDLEIGVAGDQGMPELEFPDLSLLPESPRL

>PDK 30s729341g002 Phoenix dactylifera MEMRGRKTRKGGGGWRKRRARRCRNGPNSVSETIAWWKEHNHKLECASDGHKHIQRAPGKGSRKGCMEGKGGPDNPHCKYRGVRQRTWGKWVAEIREPSRGSRLWLGTFPTAVEAALAYDEAARAMYGSYARLNLPEFNSCANTPITTSESCESTTTSHHSNVSDVSNPRQPIVIMPRQEPEDEIRNNGLPPAAAAAVAKDEPGEELFGPLDQIEDLPEDRFDIGDMLRAMDADLTNRGAGGEGINGIACQSGPVVGDINWQCSSPSAFSFQLQNPDAKMLGSLHHMEQNPAGMDCGYDDFVRQMEHGLPDDSAMPELGFSDSSFFYNGHEEGGGE

>PDK 30s765571g001 Phoenix dactylifera MDNLRKSPLKPWKKGPTRGNGGPQNATCEYRGVRQRTWGKWVAEIREPKKRTRLWLGSFATAEEAAMAYDEAARRLYGPDAYLNLPHLCSNINSTITGKPPHRFKWFPSKNFTSMIPSYGLLNLSAQHNVHVIHQKLQEFKNSRSSSSSSSCRSLFQSLEQPPPAAPVEETLLENSNMRVEEASVMPEKPQIDLKEFLQQLGVLKEEESKPEADEGAESSVMAVPTQESSGVASEMVGFDEADFNWDTLVEMQAWADHSVIEDGGLSETSMILDATRAVSEPQIFLANPRDDNAEISRTFVACRRALSTTALGCAPLSSKIQALLSSQRR

>PH01000022G0050 Phyllostachys heterocycla MGGPENMKHNYRGVRQRLWGKWVAEIREPNHGRRHWLGTFDTAVDAALAYDRAARAIHSPLAPVNFPAVSTAAAPGQCEPASCSLSAAAVSVFEEHEVEPVPAQGGCTGNAQHRDASSAAPEEMFADYLDDIAMYIDVDAVTEMVASYPDIKSEDWQVDELDTDVVDSPLWGAG

>PH01000098G1180 Phyllostachys heterocycla MRRKSTGPDSIAETIKRWKEQNQKLKEENASRKAPAKGSKKGCMAGKGGPENSNCDYRGVRQRTWGKWVAEIREPNRGKRLWLGSFPTALEAAHAYDEAARAMYGSAARVNFSEHSVDANSGCTSAPSLLMSNGPTAALNQSDAKDELESPPFLVSNGPTAVLHQSDEKDELESVEAELHEVKTEVSDDLGNIHEEQKTLEVFQPEGSVLHKEVNVSNDYFNVEELLKMIIVELNADQKMEVHEEYQDGDDGFSLFSY

>PH01000188G0980 Phyllostachys heterocycla MESYGRKRAWKKGPTRGKGGPQNAACEYRGVRQRTWGKWVAEIREPNKRTRLWLGSFATAEEAALAYDEAARRLYGPSAFLNLPHLRAASAATAHQRLRWLPASTRGAVAAVPAYGLLNLNAQHNVHVIHQRLQELKNSSSPTKPPRTPPPPPARAHLPLLPSTSPCSTVTTNAPASAALPPPMSCFHALEQAMSAVDSAPCDAAAGVGVDKPQLDLKEFLQQIGVLKADEDGAAGKEDIHGDGEVADAFGFCGNGEFDWDALAADMSGIAGGHGGAVGINGGFQMDELHEVDQFGYMPIPIWDI

>PH01001487G0410 Phyllostachys heterocycla MKGKGGPENQRCPFRGVRQRTWGKWVAEIREPNRGARLWLGTFNSALDAARAYDAAARALYGDCARLNLSAASSPSRPGLQQLRPPHISTAAAAGAAAHDLHLHLQDKPSPPCCSADGANSNSSVTTGSPTEDNSVHTETVPMNAMQMAMDQEAEEDFEAYVTRLPRAEDFGLEGFQEVPLEVLDEAGGGISIWDLTICPDMMPASTTPPPKHQPDVEVVDNAGLQRLAW

>PH01002279G0250 Phyllostachys heterocycla MASSSNGKGKQQSSKKCCPLRRSRKGCMKGKGGPENQRCPFRGVRQRTWGKWVAEIREPNRGARLWLGTFSTALDAARAYDAAARALYGDCARLNVSASGSPSPDTTAGLQPRPPHINISTAAGGAGGGHHQYKPSAPGTPCCSADASSNSNSNSNSNSSVTMGGSPTDNNSVSMAMRTETTQYGMNTMQMTREEAAEEFEAYVTRLPKAEDFGLEGFQEVLDEAGGGIGIWDLTICPDMMPAASTPPPKQQPDMVVDDAGLQPQSQPLAW

>PH01003928G0080 Phyllostachys heterocycla MRRKSTGPGSIAETIKRWREQSQKLELEEENASRKAPAKGSKRGCMAGKGGPENSICDYRGVRQRTWGKWVAEIREPNRGRRLWLGSFPTALEAAHAYDEAARAVYGSAARVNFSENSTDASSGCTPAPSLPKSKGELESVEAKLHEVKTEVSDELGSIHDERKTLEVFLFRPEGRVLHEEVDVGYEYFNVEELLEMIIVELNADRKMEVHEEYQDGDDGFSLFSY

>Pp1s120 13V6.1 Physcomitrella patens subsp. patens MKGKGGPENAQCSYRGVRQRTWGKWVAEIREPNRGSRLWLGTYGTAEEAALAYDEAARVLYGLNALLNLPDRGPTAPASNFGVSEGSRNNQENSGLGYRKSNLGGGPTESAASGTVPDGQDEGLDTADSGSPRKLLEASIAEMSRTSPRRLAAEAILPESSSSWLEDNDAHEPNEALDLEKVRNEQIVFNDLLDVEDLDILDPKLAELPRLLRSNSDSSTMTDSSVFSKDLWEELACHMTISDGTLESGKSNDSSSLTANNMEKHDVRCDDVEGICISDGQLVSPYSPEVEMLDNSPLMQKKQAWTTLLSDQASPS

>Pp1s162 92V6.1 Physcomitrella patens subsp. patens MTVSGRTLQAERGRGEVVITVAKSKVSGVVNSANIGISSNFPSAHWEAIATKTIQRIGMQLGSGGDDKLSPTSGHIEKPARKPGKRWKKGAMKGKGGPENAACEFRGVRQRTWGKWVAEIREPKKRARLWLGSFSTAREAALAYDIAARKLYGSMAELNLPPSESAGIDSVPSSTPTKESSSPPRVMHDVEITEHLELPRQHSLGTSASAGSSSRTSMHIGIREDHFDVELVMADSGRAFGASDARQVFPHEMVRDSRGMGEAGPSLEMGTDSSSSSQVNPPHELFSITPEFQQHRYYNLQGSRLGTSLSGEYLKDIESFLNRMDDDDDYALGSNSSLSQSTESAGGSYPPDPHWDDENLGTQFLNSELESLDSVLNEPLVPENVELWDTRDTLPPSDPSTWQ

>Pp1s23 27V6.1 Physcomitrella patens subsp. patens MGKTDAGSGSSRSCTKKNMRQAPAKGSKRGCMKGKGGPENALCTYRGVRQQTWGMWVAEIRKPNRGLRLWLGTYSTAEIAALAYNSAARILYGPNALLNQPNRTPNGSNPDEHMDRTTATYSSASSAEIGNLGGKSFQKIERVPCKLRSGGAEPHSQAGKEGRSAASFTTPESLADHDIDTATSLQALQMLAADVVLPDSEPPKPWPCDPKPEFIDKAWLQESNQPSFLPPKQLIKDVLENDHDIFNFKLDNGSDLAPPLQLETTSGLNEITTTIGAFSEARWTKNECGSDMPEAMPESDQSNGSTFTSTTHTMDTTVDVASLGDLNDEDLQILMQLWSPELASYAWNAASVELSVSR

>Pp1s292 5V6.1 Physcomitrella patens subsp. patens MHLGNQGNARLSPTSGHIEKPPRKPGKRWKKGAMKGKGGPENSACEFRGVRQRTWGKWVAEIREPKKRVRLWLGSFPTAKEAALAYDIAARKFYGPLAELNLPAHESAGLDSVPSSKRTEQSSSPPRKMHDVAIPELLEFPHHRPLETAVNVGSSGRTSLHIGIMEDFNDDEIALANFDSSIHDLDIRDILLRETVGDSRGKVDPTPSPDVSTTSSSSLRVYPPQQRFPSAPEFEQQQYYSSQAERAGTFSSCEQPQDIDRFLHQRDTYSIPSFRPDDYYDGASGSNSSSSPSIDSAGGSYPAGVYWEDEDLGSQFLNLELQSLDSALREPLFSDC

>Pp1s81 44V6.1 Physcomitrella patens subsp. patens MQHGSSVAETLAWWASRNNGIGGKAGANQADDSNGPRKIIRKAPARGSKKGCMKGKGGPENAMCNYRGVRQRTWGKWVAEIREPNRGSRLWLGTYPTAEIAALAYDSAARVLYGSNALLNLPGETASPVAGTASTSATSASSAEIAISDDRSSAKSERVTAKRTPAKHTGKPNNSAASFTDPDPSATLDTATSLSQHQAAEVALSYDLLEPLPRHPKPEPVQELQLSAEPSFPPLLQEPQEPMDCNDLLGKEFDLFDFKMDEGDMQLPPSLKTNSNSSTMTDSSAFSRDLWTELACHGDISETVLDSDHSIASSTTLGEDKPEDVHVLRSLTEDDMQRLTMPESPEVTLFEISPSLNRKEAWTSLLDVGAHRIPVYIQVLCQDWGCGGL

>MA 16778g0010 Picea abies MSGQVRATSPSFPATCAMLVPRHDEHWKSPITRGNDNGGHSGSFVRLLNLRRAFYIDIHCVYRFSNLRGPGVSNPNVQINPRNGRTSRCMSSSSLTINTVKDKHVATEPQRRRKVRSRNHGCTTVVETLAKWQELNSQVESSKDGAKRLRKAPAKGSKKGCMKGKGGPDNGRCNYRGVRQRTWGKWVAEIREPNRGSRLWLGTFSSAEEAALAYDQAARVMYGSCARLNLPDISSKESSVTSTSTTHVTCQSQECSTSQQYEVSSEWKDSKSSPLDKYGLSPVPSSQSPGIDAVSEAGVEVRNGGRIADTDQRYGSEICSGVEAAKSDFPSKVEISAALPDSAVDLPVKSDTKECTLIPRLVPKDEPQDADGRNTFDLSQTTLLRFRDEPQSELPSAAVSIALSNPEVQPISIKAEQNESAEPLDSFQDIQLQDLDEMFDPDELLKMMNGNENKGELSEADTQMLSSLYYSSWPQEELTDGDVLPSCDPSSSPTLQYLQFQSPDYRMLQNPVVEDEGEQLHLDDRNFKGLGQLQQFNLDSFQQSEYQAVKQHVRFDDRQPLHPKVLDDGHFASFPYPYNNQGYQTEDTRTRQVVSPESMLTDDLQGMQDSRIFEDIFSSYN

>MA 19420g0010 Picea abies MRGGSRRINTATASSSTTSDNNNNIDDNGSLSSMLCSSIHNKEFMGFSAPKKQIRKIPAKGSKKGCMKGKGGPENGLCNYRGVRQRTWGKWVAEIREPNRGARLWLGTFATAEEAALAYDKAARALYGSCARLNLPEPRLNSENGPNSDTNLSSFNASAASTNCSTDAPERTFQLSSSATKRRSSHHHHPSKVCFDKNVVRQSFSDQDHNINNNNGQLQHFSDSVNSVSLDSVSASNYDGEVLGNDHHSAAIVSATTNSNSSSYDNTNYNDHNNIIKLAGFDDLSVEKGTPQFSRIDAMEQHGGDNLINNEWSNLAANLDCSHASNTESEISRNERKYNLSLDEKHMMDNHDLELMDSAWPPLQALPLSLELPPLEDFATYKVQVGEDDLKMEGFLPSMESADIMSLRNWQTEGYDPFHFSCCFYD

>MA 19683g0010 Picea abies MKGGNREARCSAVGQENGGLRSTPSAPPSRKRICKGKGGPDNIKFQYRGVRQRSWGKWVAEIRQPGKQTRRWLGTFATAEQAAQAYDNAAILLYGSRAHLNLQPSGWDHSKSSSHTSKLRPLLPRFTFRLPPAIHGTNPNPNPNPNCFGAIPSGYFQTPTNPDFWPAAMRAAQTDATYNLPVIDPKRETTLEQSSVLHLEEVRVPTQQCNAEVHYESSSLKIDNLQENYGAEITDLQSHKLGGPDQLSGIHGGVVGDELHQGFYSDTTGNIIEPRNHIDGESRDDNLAASLQELQYSAAPPSPGFMWHYNIKHDYYYEETNSSTLPETNNQLWDYSDESSI

>MA 214063g0010 Picea abies MEKRSSPERRFDMRRATKVGTKAWKKGPTRGKGGPENATCEYRGVRQRTWGKWVAEIREPKKRTRLWLGSFATAQEAAMAYDAAARRLYGPDAHLNLPEKYERSLYSPSSQGSHLLAHHASWYFPRGLLDHSRSRIPVIHPTFSSFSPACPSAVTQRASCINLNVQPNVHHIHQKLQEMKSRNEVRFPRLSMNDRFFMNDQINASSTLLPQDIGQISTDLAPRHLSSQEVAAAPQPGINTDQENNSKGSIGSQQVDLREFLEQLGVIQAASPPPPPPPPPPPAWDDADSECTSIPISNTFSPLPASSQDMVSFEEPLWDSIEIPLDFMETAFLLQNQYSQVQVVEDQSFPVSIWDFQDPHQQQQNCTEDHNSPPTNQLVPHS

>MA 904750g0010 Picea abies MALIGGATKQIRKTRGSKKGCMKGKGGPENALCNYRGVRQRTWGKWVSEIREPNRGARLWLGTFDTAQEAALAYDDAARALYGSEANLNLPDSIKKPTGFQIQPACNNNMQSDVFTDHVDGSVDTIPDSKDQQVSLSTANEIRVEDIPAMINGSTSINQPLDELLSVDSHESNFMEGLELESISSISNSCIIHDQISSLDFIDENDSSQIFNGDVAWLPLDLPPLNDDATTSFELPEQESDTVKATLDDHILNIQNNWLPL

>Potri.002G029400.1 Populus trichocarpa MSKSIMSGFGEKKQFKKPEQASSRKGCMRGKGGPENALCTYKGVRQRTWGKWVAEIREPNRGARLWLGTYDTSHEAAMAYDAAARKLYGPEAKLNLPELQVNSSQFPASPANSQVIQMTNQPSHLIQNSSPTSTYSSNIPNMESNEAKPILYNHNPIMSFSNESVDSNGMEVENDANFGRSGDVIKEFSANLNVNMTFNDSIWAEAALSINFPVMDDPGIFASNLMEESGGDTMQTPWCM

>Potri.005G233300.1 Populus trichocarpa MGGMSKSTTSGFGEKKQFKKPAQASSRKGCMRGKGGPENALCTYKGVRQRTWGKWVAEIREPNRGARLWLGTFDTSHEAATAYDAAARKLYGPEAKLNLPELQVNNCQFPASPANSQVTQMTYQPCQIIHNCSSTTSTCSSNTPSIESIEMTPMLYNHDPIMYFSNESVDSDGKGAVNEVNFGRSEVEIKEFWSNFNVNIPFDDSIWVEAAMSINFPVVEDPGIFASNLMEGTGRDTLQTPWCM

>Potri.006G054500.1 Populus trichocarpa MENCGKSPLKPWKKGPTRGKGGPQNAMCDYRGVRQRTWGKWVAEIREPKKRARLWLGSFATAEEAAMAYDEAARRLYGPNAYLNLPHLQSNSSPPNKSHKFKWIPSNNFISMFPSCGLLNIHAQPSVHVIHQRLQELKNNRPLNQSSVASSSSSSESRTEVMIVSDENYVANVSAAEKDEEISSEKMLLTNHDEKPQIDLNEFLQQLGILKEENQPDNNDVDEHFTEPESSHKDQNELTALADKSFDWDSLIEMHGIADHQGEEFNSFPVYDIQKELAFPTSIWNF

>Potri.006G104200.1 Populus trichocarpa MEDSLRQSSLLGFSSKDRKRRRNGCESIEDTLARWKKHNKLQVSKVPGKGSKKGCMKGKGGPENMNCRYRGVRQRTWGKWVAEIREPVKKCSLMNKQGSRLWLGTFSTAIEAACAYDYAAKLMYGPNAILNFPDYPVQSGNHLDNMSSSITATETSSTESRTALDSYEDNKVDKLKINHCGSREGNNQSGFSRICAVDESEEEVEKIRVAESSAMELKAVEWNLTDDWKSSHHIEAEAPVLREEIDGECAGILRSWGCYEHKKLKNEVVESSMSTRLNECVDSDYDMRTDHKPIYDVEKPLMREAAAGEEFSGLKFSNYNPFETTHDHMNPGLCNQEIDIKPFIQDISDNSVLKGGGNYGYDPAKVGSASHLQSRRPSGLSCQLQTPSTNLPGSLSYFQEADIGLGWNFDLSQQDFNGGLVGEPGLLDQWYPELQF

>Potri.008G073600.1 Populus trichocarpa MGTLIQGSNATSMSMDSTKKRKRASDKSVAETLQKWKEYNEHLDAQGDGGNKPVRKVPAKGSKKGCMKGKGGPENSVCNYRGVRQRTWGKWVAEIREPNRGPRLWLGTFPTAYEAALAYDNAARAMYGSCARLNIPEVVNSTSSSKDNFSAVTPSYYSSAASPADSVTTSTHSEVCAYEDPNQNVLSQAEDWMTNISSQAEVCEQNVSSQAEVYEQNVSSQHIEDCSRGVEKNSKLSQDELKIQSENPSWTNDWHSYGWDEIFSVEELLGDIDSGMTGAEGYFSLGF

>Potri.010G183700.1 Populus trichocarpa MGTLDQYSKATSMPTDPTKKRKRVINKSVAETLKKWKEYNEYLDSQGDGGNKPVRKVPAKGSKKGCMKGKGGPENSVCNYRGVRQRTWGKWVAEIREPNRGPRLWLGTFPTAYEAALAYDEAARAMYGPYARLNVPDVLNSTSSSKDNFSSATPSCYSPAASSADSATTSTHSEVCVYEDPKQNVYSQAEVCGKDVSSQAEVLAHHISSQQHIEDGSQGVENSTLRDEQKSQSENPLWTPSENSLWTNDWHNYSMDEIFSFDELLGGIDAGMMGAEGYFNLGF

>Potri.016G053200.1 Populus trichocarpa MENCRRSPLKPWKKGPTRGKGGPQNAMCEYRGVRQRTWGKWVAEIREPKKRTRLWLGSFATAEEAAMAYDEAARRLYGPDAYLNLPHLQSNFNPLNKSQKLKWIPSKNFISMFPSCGLLNIHAQPSVHVIHQRLEELKNNRPLHQSSVASSSSSSESRNEVMIVSDENHVANLAVAEKDVEISSEKMLLRNHDEKPQIDLNEFLQQLGILKEEKQPDSNDVEECLTVPESSQKYENELAALADKSFNWDSLIEMHGITDHQAAELNSFPVYDVQDEPAFPTSIWNF

>ppa007606m Prunus persica MGAYDQGSNEVALTLDSTRKRKTRSRRDGTSVAETLEKWKEYNKQLESDNNEGKTRKVPAKGSKKGCMKGKGGPDNSRCNYRGVRQRTWGKWVAEIREPNRGSRLWLGTFPTALDAALAYDEAAKAMYGPAARLNLPNVANYSSWKESSQETSSATTPSGSSAVATPGCSGSTSSSNHSEVCADEDSKGFLDVKTENGEGESAIHAWSNAVNQALVKEETKDEFTEGYFHNDQPAVKPEAYVGDFNWAGGQYTGDYLENFTEEEVFDVDELLSPLDDTPVRNPEPEQSLGSDVDAKLLGSLNHMEQGPSGGEYNFDFLKRDDVTGEDDQGYYNLGLSDLGLGDFTGGTMQPEDGSYNFSMDM

>ppa009616m Prunus persica MENCRKSPLKPWKKGPTRGKGGPQNASCEYRGVRQRTWGKWVAEIREPKKRTRLWLGSFATAEEAAMAYDDAARRLYGPDAFLNLPHLQANSNPALKSQKFKWFPSQNFISMFPSCGLLNINAQPSVHVIHQRLQELKQNGVLGQTTPSSSSSSCDSKPEVRTISDKTQMGNVAEKEKDVEISSEKIIEDNREKPQIDLNEFLQQLGVLKKERQSEAIDTTGNFGVPESSVTELNDEFGPFADKNFNWDALIEMHGISDQGADAGSFQVYDVNEELPFPTSIWDF

>ppa022733m Prunus persica MTKLVKTDPRQDGSNPVAETLAKWKAYNDHLDSCNDESKPIRRVPAKGSKKGCMKGKGGPENSRCNYRGVRQRTWGKWVAEIRTPNRGSRLWLGTFPTAIEAALAYDEAARAMYGSAARLNFPNISISSLSKDPSSRATLSAVSSLATSA

>ppa022996m Prunus persica DKKLPKRRNGCDSIEDTLAKWKNYNDRFDFAKDGVKKKRKAPSKGSKKGCMKGKGGPENSDCVYRGVRQRTWGKWVAEIREPNNFSGVSKKNSRLWLGTFPTAYDAAFAYDEAARAMYGGLARLNFPQNTMKLKEYSNSVYSGTTKTTTSSSYESSTTYNNGDEAEGLDMSSKCAMESSRFVVGKSKESYDFFEDCVHKEPKQETDCSVGFASQDLEVLDATLREAKDVKCELETEYELARNDAEETSVFQTGSYESFNHRPDYLHNELQVVNLDSVPDGKPYYNDWESLETFLRSNNDYLNNELVDAECNGRNDCNPSEDVNVEKPVTSEAMEKEFPMILESGSHNGLDDSCHYMHNEQTNVASNLVTADFEPSNDEIKRSMTDQELRGGLVETTNLNGHNGFIDSYACLDDIRPSNDIKMQENRLANLHNWSVEESYGIDAQNQQGERLPNLPNQSQTQPQGNIPGRLAHTEEASLDIDFDVDLFRQNYDSGVLEEQVFHGSWFPYS

>ppa023248m Prunus persica MLKSEMSSVKVGERKQVKRPAQASSRKGCMRGKGGPENAMCTYKGVRQRTWGKWVAEIREPNRGARLWLGTFDTSHEAASAYDAAARKLYGSQAKLNLPDQYHHQVPSSANTHMLSQTPNPSAQLTLLHQNPSGTSSACLSNDYAAPLNDVAVPVLRDHFPNVKAHQPHGTYPEPKPEAEHSKMKVEENMSGNEGMESGIFWGNVSGNFPMFDDSIWVEAAMSLDFPVIEDHGIFSSNFVDGSVWEPLQPSPWCV

>ppa026176m Prunus persica MEKEGRKVGKKATASMGRSRKGCMKGKGGPENALCSFRGVRQRTWGKWVAEIREPNRGARLWLGTFNTSTEAALAYDEAARKLYGSSAKLNLPDHKPSSSSTANSSCSRT

>Pbr005893.1 Pyrus bretschneideri MSTSEMSGGSSSVGVAEKPAKRPVQGSSRKGCMKGKGGPENAMCTYKGVRQRTWGKWVAEIREPNRGARLWLGTFATSYEAALAYDAAARKLYGSKAKLNLPDQQAPNYRHRQLNQHLQHQVFPSSANPGVPPQIPNQTLVQNNPSGTGTNPACYIRCCCERYCSTCYSQ

>Pbr005895.1 Pyrus bretschneideri MSKWEMGGGSSSVGVPEKKLAKRPVQGSSRKGCMRGKGGPENAMCTYKGVRQRTWGKWVAEIREPNRGPRLWLGTYDTSYEAALAYDAAARKLYGSKAQLNLPDQQAPKYHHHQLNQHLQHQVFPSSANPRVPPQIPNQTLVQNNPSGTGTNPACYAASVKDIAVPVIHNNFPNVKVDRPHGNYNSGEAETLEQGGMKVEKNMSSGVNNEGINGIFWGNMSDNFPVFDDWIWTEEAMSFDLPVVADQGIFSRGFVDGNCWETSHRPNGA

>Pbr005897.1 Pyrus bretschneideri MSTMEMSGGNSSAGVAEMSGGSSSVGVAEKKLAKRPAQGSSRKGCMRGKGGPENAMCTYKGVRQRTWGKWVAEIREPNRGARLWLGTFDTSYEAALAYDAAARKLYGSEAKLNLPDQQAPNYHHYQLNQHLQHQVFPSPANPGVPPQIPNQTLVQNNPSGTGTNPACYAAPVKDIAVPVIHNNFPNVKVDQPLGNYNSGGAETLPEQGGMKVENNMSSGVTNEGIDRIFWGNISESFPVFDDSIWTEAAMSLDLPGVADQGIFGCGFVDGNFWEHRTRLNGA

>Pbr009571.1 Pyrus bretschneideri MGAYDQGANMSSLPLDSSRKRKTRSRRDGNSVAETLEKWKEYNKKLESGNNEGKTRKVPAKGSKKGCMKGKGGPENARCNYRGVRQRTWGKWVAEIREPDRGSRLWLGTFPTAIDAALAYDEAAKAMYGAGARLNLPHAANYHPSSSLETSSVATPSGSSAVATPGCSTPTSTSSRSEVCGDEDSKLFLNVKNEDGEGESRMYPWSTAVPQASGMVKPEIIEDFTMDYLRNNQQQPVVKPEAGVEDHNWNGGDGFIGDYSENFTTDELFDMDEMFDVNELLMPSDDISLCNSGSEQVWRADVGQSGMETLSSERPSNLLYQLQFPDAKLLGSLQHMEHAPLNFEYGFDFKKQEKEGSNHTGQDDQGCFNLGLSDLDWGGFTGGITQSGDGGYNYSMEM

>Pbr010949.1 Pyrus bretschneideri MGAYDQGVNMPYLPLDSSRKRKTRSRRDGNSVADTLEKWKEYNKQLESAKEEGKKRKVPAKGSKKGCMKGKGGPENARCNYRGVRQRTWGKWVAEIREPDRGSRLWLGTFPTAVEAALAYDEAARAMYGDGARLNLPHGVNNHPSSSQETSSVATPSGSSAAATPGCSTSTSTSSHSEVCGDENSKLFLNVKKEDGEGESRMYPWSGAVPQASGMVKSEVSEDLDVDYLRHNQQQPVVVKPERGVEDHNPNGGDGFVGDCSENFTMCELFDMDEMFDANELLMPSDDTSFCNSGPEQVSRPDVGQPGMEILSSEGPSNLSYQLQFPDAKLLGSLPHMEQAPLDYEYDFDFMKQEEGSNHTSQNDQGYFNLGPSDLGLGGLTEVELVNAMKGSFDIAFRNIRMFNDVVNANAFMKN

>Pbr015926.1 Pyrus bretschneideri MESEASGGERKLRKRRNGCDSIEDTLAKWKNYNERLDFGKDVGKKTRRTPAKGSRKGCMRGKGGPENSDCVFRGVRQRTWGKWVAEIREPIRARAGSVPQKKNMRLWLGTLPTAYEAALAYDKAARAMYGALARLNFPDNAVDSKDYYSNSVSSKTPSSHESSLTYNNADEAEGRPAFFEDCVAKEQKQETDCSVSEELLVLRATSREPKVVKCEYETERELVKNDDVFQTGSYGSFDHWGDYLPNEPPVVNFDPVFDRKPCNDLDALEILLRSNYDYLTEVVDGECKRRNSCKPSNDVKVETPAMREAAEKPFPVILESGSHNGLDEKYNYMHGEQINAAENHVTNFEPSEDVEMKLSMTDQELQGGFAETTRLNGHNHDDFIHSYACLDDLDVAYSPIYGINPWNDIGMQKELDDRLDYVHNWPAEETYDIDAAEDQQWGRTHNLPTQLQTQPHLDIPGSSNHAEYARLGVDFDSVLFRQSYDSGEMEDQGPPK

>Pbr028399.1 Pyrus bretschneideri MPKLVKIDTRRDGPNPVAKTLAKWKEYNDHLDSCNDEVGPFRRVPAKGSKKGCMKGKGGPENSRCNYRGVRQRTWGKWVAEIRTPNRGSRLWLGTFPTAIEAALAYDEAARAVYCSAARLNFPNISISTLSKDSSSTKTLSSLPLLASSAGSESSARSDHSGDCADQDEDQVNGLSSNVENDDTTNVDRTNEDRDDE

>Pbr038530.1 Pyrus bretschneideri MDNSRKSPLKPWKKGPTRGKGGPQNASCEYRGVRQRTWGKWVAEIREPKKRTRLWLGSFATAEEAAMAYDDAARRLYGPEAFLNLPHLQPSSNRLFCYG

>27810.m000639 Ricinus communis MENCRRSPLKPWKKGPTRGKGGPQNAMCEYRGVRQRTWGKWVAEIREPKKRTRLWLGSFATAEEAAMAYDEAARRLYGPDAYLNLPHLQPACNSINNIPLNNKPHNKFKWIPSKNFISMFPSCGLLNIHAQPSIHVIHQRLQELKKDGVVGQSSNHASSSSSCDSRNEVMVENDKTQVENLAEVEKEVEITSEKIVRHEEEKPQIDLNEFLQQLGILKVERVQQPESDDATEMSSVVQESSLNDDHQENVAAALADKSFNWDSLIEMHGIGDHQGAESLSTFQVYDVQEELAYPASIWNF

>29680.m001737 Ricinus communis MEKEATDKKVTMRRSRKGCMKGKGGPENALCTYRGVRQRTWGKWVAEIREPNRGNRIWLGTFNTSHEAAKAYDQAAIKLYGSSATLNLPHSYDQQRQRQEEEEEEEVPFSNRSTTPLSSSFASACPDYVGTCGGSTNSSCRDQPSATNLFLSSIGADASGCGISGGEGSSGDINAYWPDFGFGENYHFLETNDFGVAAMGGEGFNWDGSRDLWSF

>29729.m002292 Ricinus communis MGTFGHNSNASSMQLDSTRKRKRREGTSSVAETLKKWKEYNEYLDSCAKGEDNKPVRKTPAKGSRKGCMKGKGGPENSECNYRGVRQRTWGKWVAEIREPNRGPRLWLGTFPTAYEAAVAYDEAAKAMYGSSARLNFPELSSSSKDDTHSVATTSGGYPSVAAPAGSDSTTTSNHSEVCVLDDTKEHVVKLGDGEGESKITPHSDPLTQTASATTTYKQELKSELENVKESDRGEVPVKEVPIQNNACDLKQKGADDLQPVPKDFSLNVEEWNLLNENEGQGQDIWQNFTMDELFNVDELLGVIDNYPLDMQFDGGQLLCTDNNQLQHDQPLDLSFQLESPDTRFIGGYQPSEQVPSGGDYSFDFVKPGRQEDNNNVPLNDQGFFM

>29739.m003716 Ricinus communis MVKHTSAVEKPVDSSYFKFKGVRKRKWGKWVSEIRLPNSRERIWLGSYDSAEKAARAFDAALFCLRGRSAKFNFPDDPPNIAGGRSLSPSEIQAAAARFAKSEHPKSVQSDHSESELQVXWIFPGFDDLFAPLSEPSLDFGEENFDGVIDQDSFLWSF

>29814.m000739 Ricinus communis MDDNSISLTQYPLQENQETTTTAATTTTTTTTTTPAAVTSDNNSDSNNNNNSSSGDNNGNSRKCKGRGGPDNNKFRYRGVRQRSWGKWVAEIREPRKRTRKWLGTFATAEDAARAYDRAAIILYGSRAQLNLQPSNSSSTQSSSSSSSSSSSSTRASSSSSSQTLRPLLPRPSAFGFTFSLSSTPPAVSSEFGTYGVFQHQNQNVNVGSSPVLCPANIVQNQQEQLLQSHHYQYHHQYQNPFLAGSSNFITCDPIIIPATTTTAAEATATSYHQNLNYVYNDHIPHQPQHHQEQQHGMYQDIASLVGSVGSSLSLSSSTQPVIAPANQDPVMHVGPGSPSVWPLTSDDEYPPPSIWDYGDPSIFDL

>29820.m001008 Ricinus communis MSGMGDTKKESSSSGISRKSKKNSRKSGSESLEDTVTKWKTQNQLDEFRKPPSKGSRKGCMPGKGGPENQSYRYRGVRQRIWGKWVAEIREPAGKKSVLMNNKTGNRNRHWLGTFSTAIEAAVAYDNAARAIYGPNAILNFPDYSSETSSTQFKTTLGSSEDDHTEKSRLNLLSTLQKTQCSVVREFKEESEKLGSSGISVIEESNEKPENSQVAESILEEFKDKESILKPLYRYSNDAKVEETLLVKKINPEFADNMKYPENYGINGRHDCLQGKTKNMKAELSTDYEHCNEDEVMAPIIKHVEEEEAGRRKESRQLGLSNTNMHESFNDSSGRDCELRTDYKSFNDSQIPMKRKVIEGEFPGMGSQFEARPDDLSNNSMDTECYREIDFKPSIQIGETDHSAREVKMNYGHYHLASTNYQESNRSAHLTFQWQNPRDDQSGSSNHTVETIRTEDIDFNLLGPDFNWGSVEELGGIEQWFPEFGF

>30170.m014115 Ricinus communis MMSKSTVSTVLGEKKQLKKPAQASSRKGCMRGKGGPENALCTYKGVRQRTWGKWVAEIREPNRGARLWLGTFDTSHEAAMAYDAAARKLYGPEAKLNLPELHSSKNKVPASSANSQASQLGNRSQSQILHNSGATCPSSGPIIRANNLVTPAYSYDSIVSFPNANVDCCNANVAESNEASIYGQNQEGAGINELWENLNVNLPLFDDSIWAEAAMSIDFPAMEDPGIFASNLMDGTNWDALQTPWCK

>27203 Selaginella moellendorffii SPAGSSIKKAPAKGSKKGCMKGKGGPENPLCHYRGVRQRTWGKWVAEIREPNRGSRLWLGTYATAEEAAMAYDDAARVLYGSCARLNLPVSGSAGGQRSQAINLDSSRQTSSRKKMAIKEEEEEDCDSGVKMEEDDGESSISTS

>38494 Selaginella moellendorffii RPQLKRSRKGCMKGKGGPENAACQYRGVRQRVWGKWVAEIREPNCGARIWLGTFDTAVEAARAYDQAALKYFGENARLNLP

>38500 Selaginella moellendorffii SKKGCMKGKGGPQNSQCQYRGVRQRTWGKWVAEIREPNRGARIWLGTFTTAEDAAAAYDRAAKIHYGPSAQLNFP

>38502 Selaginella moellendorffii SKKGSMRGKGGPENASCTYRGVRQRTWGKWVAEIREPKKRSRLWLGSFATADEAARAYDEAAKRLYGPEAHLNLP

>Si002067m Setaria italica MRRLAGRRAGGCVTLVGGIGNACVDHAPAPPPTASRQVSRTASWRPRQNRSGGSVRGRPAAALAAGGNLGRRRRRRVGGGDLPSLSSMDLGHGAQGGEGDSSGSGGQLRKKRMRRKSTGPDSIAETIKWWKEQNQKLQDESGSRKAPAKGSKKGCMAGKGGPENGNCPYRGVRQRTWGKWVAEIREPNRGKRLWLGSFPTAVEAAHAYDEAAKAMYGPKARVNFPENSADANSGCTSALSLLASSVPAAALHGFNEKDEVESVETEVHEVKAEANDDLGSIHVECKSVEVLQSEEIVLQKEGNVSYDYFNVEEVVEMIIIELNADKKIEVHEECLGGDDGFSLFAY

>Si022619m Setaria italica MTVDQKQAMPMQAQAMQPGSRKKRPRRLRDGPTSVAAVIQRWAEHNKQLEHDSEGAKRPRKAPAKGSKKGCMKGKGGPENTHCGYRGVRQRTWGKWVAEIREPNRANRLWLGTFPTAEDAARAYDQAARAMYGEVARTNFPRQNAVASSQVAWAATPAQVAPSVVEGVVHSTSCESTTTSNHSDIASTLHKPEVSDLSSSVKVECPEVVEAGSRRSEMVSGTSHQHEDSHPSTQASTPNVGDKEVFEPLEPIANLPEGDFDGFDIDEMLRMMEADPQNEGGAGAGMEQPFYFDGLDSSLLESMLQSEPEPYSLSEEQDMFLAGFESPGFFEGL

>Si022621m Setaria italica MTVDQKQAMPMQAQAMQPGRKKRPRRLRDGPTSVAAVIQRWAEHNKQLEHDSEGAKRPRKAPAKGSKKGCMKGKGGPENTHCGYRGVRQRTWGKWVAEIREPNRANRLWLGTFPTAEDAARAYDQAARAMYGEVARTNFPRQNAVASSQVAWAATPAQVAPSVVEGVVHSTSCESTTTSNHSDIASTLHKPEVSDLSSSVKVECPEVVEAGSRRSEMVSGTSHQHEDSHPSTQASTPNVGDKEVFEPLEPIANLPEGDFDGFDIDEMLRMMEADPQNEGGAGAGMEQPFYFDGLDSSLLESMLQSEPEPYSLSEEQDMFLAGFESPGFFEGL

>Si022989m Setaria italica MKGKGGPENTHCGYRGVRQRTWGKWVAEIREPNRANRLWLGTFPTAEDAARAYDQAARAMYGEVARTNFPRQNAVASSQVAWAATPAQVAPSVVEGVVHSTSCESTTTSNHSDIASTLHKPEVSDLSSSVKVECPEVVEAGSRRSEMVSGTSHQHEDSHPSTQASTPNVGDKEVFEPLEPIANLPEGDFDGFDIDEMLRMMEADPQNEGGAGAGMEQPFYFDGLDSSLLESMLQSEPEPYSLSEEQDMFLAGFESPGFFEGL

>Si024059m Setaria italica MCRRDADTQEGNTCSARAVAAAAARRCTPAKGAGNGQASSGVEGTRTHVRAGGLPSGSGSPGPSPPHRQADLTRSPRAQAAGGATWPPEVRPRRRPTLHGGAMQCQAPAGRPATRRPPLSLSIPFRLHCIGMHANCTPPSPPCAYKYRRQAARPSHHQQQQQQRLPLPPFHLVSQQPPQHQQGGSGKVAGGGGGRKCCPLRRSRKGCMKGKGGPENQRCPFRGVRQRTWGKWVAEIREPNRGARLWLGTFATALDAARAYDAAARALYGDCARLNLLPAAALPAAAAAAPTNSNSMVVKASPAASPSSSPDAAVADHHHQYYDYKQEPTTMAMNMMMVAAVPSPSCCSADGASPNSNYSNSSSSAAPTPTAMQQMMMADELAAAEQHQQAEADDFEDYVTRLPKAEDFGLGGFQEVPPEVFDEAAGGGIWDHTVGWPSAMMSGGPSQIVPF

>Si024427m Setaria italica GTGTQQARANGGANGTPGAGRSRSVSAQRGVGGPDNTRHNYRGVRQRRWGKWVAEIREPNCGRRHWLGTFDTPVDAALAYDRAAVAYHGNLARLNFPADNAAAVTIATAAPAQRQPSSCAPATTADVFEEHEVKPLVAVSQGGGGAETVSQQQQQQGASWLSPELLFDDDPNDIAMYIDFDAVAHMVPCYPGIKIEDCQPDGFDGDAIHSPLWPLGD

>Solyc04g050750.1.1 Solanum lycopersicum MEECGKSRIESVESKRKSRSRKGCMRGKGGPENASCTYRGVRQRTWGKWVAEIREPNGGARIWLGTFNTSVEAARAYDDAARRLYGSDAKLNLSEQESTDVGIIEDGDGECSVLEEASIFKDGNGKYLVWDTPAPSLLGVDFHGDATTCFNWKNQTEMMYF

>Solyc04g080910.1.1 Solanum lycopersicum MSSNSKKLIPATSRKGCMRGKGGPENANCTYKGVRQRTWGKWVAEIREPNRGARLWLGTFDNSYDAAVVYDAAALKLYGAEAKLNLPHLYNNQAQAQAQIQNSKPITIMSPSLSPVSTTTAQASSPSVTSVYNVASPSTWSVGSDDSSFYFNSHDFGIHNDIPSAFNLIDINKTADDYSVDNATNNNQSSEVLGGEMFRDLNMNLPEIDDSSIWEEAKATTSFQEAVNDPGIGGYNLDDDLNFPPWCG

>Solyc05g052410.1.1 Solanum lycopersicum MMLPMDYTRKKKSRSRKDAPKNVAETLAKWKEVNEKLDACDDDGRKPVRKVPAKGSKKGCMKGKGGPDNGRCKYRGVRQRTWGKWVAEIREPHRGRRLWLGTFDTAIEAALAYDEAARAMYGPCARLNLPDYYASSKESSKDDSSLPTVSRSDSNTASSFSEVCPAGDMMRGRANVPAARHEDRSIEIDGARTGSNEIGTPLSSLREEAEDETKEVSDKSETFTPLSSLREQAEDEAKQVLDKSETFEIKDEPAACSYDSWDIGQEDLGNFCLDDEMFDVNELLGMMDSTPVDASAPSQDVGFVPPKQEQYAYDPSYQLHSAAYDANQLSNPAYQLDNADDQFSNPLYQLDNAGVDTLEGLQQMEQQSPIEVDYDFDFLRPGRQEDFHFCLDELDVLDF

>Solyc06g050520.1.1 Solanum lycopersicum MAIMDEAANMVCVPLDYSRKRKSRSRRDRTKNVEETLAKWKEYNEKLDNEGKGKPVRKVPAKGSKKGCMRGKGGPENWRCKYRGVRQRIWGKWVAEIREPKRGSRLWLGTFGTAIEAALAYDDAARAMYGPCARLNLPNYACDSVSWATTSASASASDCTVASGFGEVCPVDGALHEADTPLSSVKDEGTAMDIVEPTSIDEDTLKSGWDCLDKLNMDEMFDVDELLAMLDSTPVFTKDYNSDGKHNNMVSDSQCQEPNAVVDPMTVDYGFDFLKPGRQEDLNFSSDDLAFIDLDSELVV

>Solyc10g076370.1.1 Solanum lycopersicum MDFYDNRKVKRRRNGSDSIEEILLRWKNFNQEVNWNHEQVKKKRKSPGNGSNKGCMPGKGGPENSGCKYRGVRQRTWGKWVAEIREPVYISGQYKSKGKRLWLGTYSTAGDAAVAYDEAAKVMYGSNAILNFPNSSNGNITRTSSGQSSIDHEESVVDDEKKTEIESDLKDDDGGVVVNMDLSYDYANHGSPACSWTEEELEVITEENSEIELTNLECDSRFFHKSHVKVERPIMEEEIDEDEFVHNDVSNTIDVEPTVMFSKDDFSRLDETRNSNDQIVLQDMDFRSSENLNEDVSTRLEYMEHFLMDDNCSMEAANISDIICLTENHDEAFDFQRFLEESFDFELNYAKNEEQFDCTYAYNQQIDHQNSETNFEIRSDGIRKEKNLHGFGLDDFGASNNQRKI

>Solyc10g076380.1.1 Solanum lycopersicum MQGKGGPENSSCKYRGVRQRTWGKWVAEIREPAYISGDNKSKGKRLWLGSFDSADEAAIAYDEAAKVMYGSNATLNFPNYSSNGSITRTSSLELSGQSCVDHEDLVLDGSKNDEIESDLKTSDTPNTDLSYDYVNHGSPACSWNEEDLEVIMEENSKNELIDSEFETPLTKEDEFVQCLNFNDVSNTKNVNEDVGARLEYMEHCVMDDNCSMEATNVLDTFCLTENHDEAFDFQRFLEESFDFKLNYAEQFDCTYAYNQQIDLQNSETDSEIRSDGIWKEQNLGGFGLDNFGASNSTQPEDNIEDLSMFSSDFDVSSFLNDII

>PGSC0003DMP400006612 Solanum tuberosum MSTTSKKLIPATSRKGCMRGKGGPENASCTYKGVRQRTWGKWVAEIREPNRGARLWLGTFDNSYDAAVVYDAAALKLYGAEAKLNLPHLYSIPAQDQSQAQSQIQNSRPITIMSPSLAPVSATAPASVPAPPVPSVYNVASPSTWSVGTDSSLYFNDHDFGISNFSANDIPSAFNLIDINKTADDHPDEFVVKNDNATNNNQSSQGLGGEMFRDLNMNLPEIDDSSIWEEAKATTSFQEAVNDPGIGGYNLDDGLNFPPWCG

>PGSC0003DMP400027844 Solanum tuberosum MDFYVNRKLKRRRNGSDSIDEILLRWRNFNPEVNSNNEQVKKKKRKSPGNGSNKGCMPGKGGPENSGCKYRGVRQRTWGKWVAEIREPVYISGEFKSKGKRLWLGTYSTAGDAAIAYDEAAKVMYGSNAILNFPNCCDSPITRTSSLESSGQSSVDHEDLVIDNAKNIEIESDLKTSDTPEDDGGGVVNTDLSYDYANHGSPACSWTEEELEVITEENSVIELTNLEYDSRFFPKSQKSCVKVETPIMEEEIDKDEFVHNDVSNTIDMKPTVMFNKDDFSRPEEICNSNDQIVLQDMDFRSSENLNEDVSTRLEYMEHFLMEDNCSMEATNISDIFSMTENHDEAFDFQRFLEESFDFKPMVVPQESAELNYAKNQEQFECKYAYNQQIDLQNSETNSEIRLDRIISNQADSKEQNLRAFGLDDFGASNSWQPEDNIEDFSMLNFDFDISSYINDIS

>PGSC0003DMP400027846 Solanum tuberosum MPGKGGPENSSCKYRGVRQRTWGKWVAEIREPVYISGEYKNRGKRLWLGSFATADEAAIAYDEAAKVMYGSNATLNFPNYCGSSSNDTSSLESSGQSSVDHEDLVVDDAEKIEIESDL

>PGSC0003DMP400041039 Solanum tuberosum MDSYKRNPLKPWKKGPARGKGGPQNALCEYRGVRQRTWGKWVAEIREPKKRTRLWLGSFATAEEAAMAYDEAARRLYGPDAYLNLPHMRANFNPLNKSQKFKWFSSTNNFVSSLIPNTTGLLNLSAQPNVHVIHQRLQELKRAEAAAATASSSSSSISDLPKNGTQNYSKLQPEYLTMTSPQVMIKEKEDEFSSHNNVAVRGDQAEKPQIDLNEFLQQIGIMKRDDHQQQPPDKNQDHDHNNNNSISSFTESGVSLKDDSSIAHLFGDTTSYNWDTLGAITGIEEDHEVAAEEANSFYNVNDELMFPSSIWNF

>PGSC0003DMP400041406 Solanum tuberosum MAIMDQAANMATLPLDYSGKRKSRSRRDRTKNVEERLAKWKEYNEKLDSVDDEGKPVRKVPAKGSKKGCMRGKGGPENSRCKYRGVRQRIWGKWVAEIREPKRGSRLWLGTFGTAIEAALAYDEAARAMYGPCARLNLPNYPSRDDSDSASWATTSASDCTVASGGFGGEVCPGEGATTAAGTPLSSVKDEGKDETAGLPGEMEIVEPTSIDQDTLKSGWDCLDNLNLDEMFDVDELLAMLDSTPVSTNDFGSDGKQYAYDNNLLSNSPCQPLGDPQEMGQEAPMTFDYAFDFLKPGRQEDLNFNSDDLTFMDLDSELVV

>PGSC0003DMP400047100 Solanum tuberosum MAVLDQTSNMMLPMDYTRKKKSRSRKDAPKNVADTLAKWKEVNEKLDACDDDGRKPVRKVPAKGSKKGCMKGKGGPDNGRCKYRGVRQRTWGKWVAEIREPHRGRRLWLGTFDTAIEAALAYDEAARAMYGPCARLNLLDYYPSSKESSKDDSSLPTESHSDSNTASSFSEVCPADDMMRGRANVPEIRYEDGASEIKFDGTRTGSNKVGTPLSSLREEAKDEAKEVLDKSETFTPLRSLREEAEDKAKEVLDKSETFEIKEEPVACSYDSWDIGQEDLGNLCLDDEMFDVNELLGMMDSTPLDASAPSQDVGFVPPKQEQYAYDPSYRLQNAAYDNNQLSNPSYQLENADDQFSNPYQLDNADVNTLEGLQQMEQQPPIEIDYDFDFLRPGRQEDFNFCLDDLGVLDF

>PGSC0003DMP400049990 Solanum tuberosum MAECVESKKSIVGRSRKGCMRGKGGPENALCTYRGVRQRTWGKWVAEIREPNRGARIWLGTFNTSVEAARAYDDAARRLYGSDAKLNLSEEEESIISSDTEISCMENNIIEECSVLEEASIFKDGNGEYLVWDTPAPNLLGLDFHGDATSFNWTDEPEIMY

>Sobic.001G378900.1.p Sorghum bicolor MDHQLPPVAMEVAAMQRQRQRQQQQFVNHLQVHQQQGTHHQPPPPQQQQQQCFRLRQSRKGCMKGKGGPDNQQCPYRGVRQRTWGKWVAEIRKPNRGTRLWLGTFGSALEAARAYDNMASQIYGDHALLNLQLPPPAVAAAAAGRGGPAVMVSSPSPNTVVAGPGAGAAAGHNRHHQYLQQQQKQAAMAAAPMMMMPYYYSYSAGASSSNSGSFSNYSSSSPVTTAAVAASPTYNYNNHQTFHMDHRPRHAAAVPQAQGCHIDDTTTTTTMEMQRHQQIIRELAAALLHQEPDDFMTRLPEAEDFGLQGFQEVPPEVFDEAARSIWDHTAAAWSTPTMMIDSTAGAAQHQQ

>Sobic.001G486800.1.p Sorghum bicolor MESHGGRKRAWKKGPTRGKGGPQNAACEYRGVRQRTWGKWVAEIREPNKRTRLWLGSFATAEEAALAYDEAARRLYGPDAFLNLPHLRASVSAAAAHQRLRWLPASAARGAAAAAVPAYGLLNLNAQHNVHVIHQRLQELKNGGSTAKPPPARQVVAPVDHLPAAASTSPCSTVTTHAALPPPMSCFHALEQAVATAAMTTVDDDAEPCEAGGACPPGADKPQLDLREFLQQIGVLKTDDDDGTATAKASFHGDAADAAGCFGGNGEFDWDALAADLNDIAGAHGGAVGVNGGFQMDDLHEVDQFGTCLPIPVWDV

>Sobic.003G058200.1.p Sorghum bicolor MELGDATAGQGAQGDAASGALVRKKRMRRKSTGPDSIAETIKWWKEQNQKLQDESGSRKAPAKGSKKGCMTGKGGPENVNCVYRGVRQRTWGKWVAEIREPNRGRRLWLGSFPTAVEAAHAYDEAAKAMYGPKARVNFSDNSADANSGCTSALSLLASSVPVATLQRSDEKVETEVESVETEVHEVKTEGNDDLGSVHVACKTVDVIQSEKSVLHKAGEVSYDYFNVEEVVEMIIIELNADKKIEAHEEYHDGDDGFSLFAY

>Sobic.007G162700.1.p Sorghum bicolor MDRSVAMEVAEMQRQRQQFVHHLQVHHQQQGTTHHQPLLSSPPPHHQQQQNSGSSSRAGGGGRRCCPLRRSRKGCMKGKGGPDNQQCPYRGVRQRTWGKWVAEIREPNRGARLWLGTFGSALEAARAYDNAARQLYGDCARLNLQQMPVPPPSAAAAGGGGGGGGGGAAAVVVSSAPSPDAVAAAGPGGGGGGGHNRHHQYLQQQQQQQAAMASSAPIMMMQYSASSYSADASSSNSDSFSNSYSSSSPVTMAPSPTTNNYNHHQTFQMTPPPSSCGVVMAPAAAPQAQQGCHVDDTTTTTLAMQHQQMIRELAAVPLHQEADDDFEDFMTRLPKAEDFGLQGFQEVPPEVFDEAAGIWDHTAAAWSTPATMMTIDSAAGAAHQHQQQVVVPLS

>Sobic.009G101400.1.p Sorghum bicolor MTLDQNQATPMQPPALQPGSRKKRPRRSRDGPTSVAAVIQQWAEHNKQLEYAPEGSKRPRKAPAKGSKKGCMRGKGGPDNTQCGYRGVRQRTWGKWVAEIREPNRVNRLWLGTFPTAEDAARAYDEAARAMYGDLARTNFPRPHAATYAQAALVSTSSQAAPMAVGALWPGTSCESTTTSNHSDIASTSHKPEASDSSSSLKAEWPEALEAGSSCIQAGTPSVADKVFGTLEPITKLPDGGVTNLPDGGVINLPDGGDDCFDIGEMLSMMEADPHNEGGADVGMGQPWCLDGLDSSVLESMLQSEPEPFLMSEEQEMFLAGFESPSSFFEDLERLK

>Sobic.009G170800.1.p Sorghum bicolor MGVGLKRQEMWRAAAAAAAAAPAPTASAAGRQQAVASNSATAKPLAERFRPAQRGMGGPDNACHNFRGVRQRRWGKWVAEIREPNRGKRHWLGTFDNPFDAAVAYDRAAVSIHGAHYAHLNFPADHAAAAPAQCHPSSCSAAATTADVFQEHEAKPRVAAALGGGAVTVSQQQPQQQGTPWISPDAPFGDDSHDIAMYLDFDAVSDMVPFYPGIKREDCQHQVFDANAVHSPVWALGD

>Thhalv10006489m Thellungiella halophila MEKPSSMKQWKKGPARGKGGPQNALCRYRGVRQRTWGKWVAEIREPKKRARLWLGSFATAEEAAMAYDEAALKLYGHDAYLNLPHLQRKPGPPLSNTQRFKWVPSRKFISMFPSRVMLNVNAQPSVHIIQQRLEELKRNGLLSQSYSSSSSSTESKTNTSLLDEKTSKGATDKMLEGDDDDDPKKPEIDLNEFLQQMGIMKDKSQAEAREVAECHSTPPWNEQEESGSPFEAQSLCWDTMIEMPTSESSTMQFDFSNFGSYDFEDDLGFPSIWNFCGSLD

>Thhalv10015907m Thellungiella halophila MEEEQPPTKKRNMGRSRKGCMKGKGGPENATCTFRGVRQRTWGKWVAEIREPNRGTRLWLGTFNTSVEAAMAYDEAAKKLYGHEAKLNLLHQQQQHQVVNKNLSFSSHGSGSWAYKLDTVRGLDLGLGTSSGSRGLWSSRLQDDDHQNSYRSLSSSGSNLSWLLPKRSSSQDQESVDAASGCGGEGGGFTFSTKLKPKNLMMTPNYGTSNGKTEYDVSSSCGSSDNKESVLVPSGGGGGEGMHRPEVEEGTGYLEMDDLLEIDDLGLLIGKNGDFKNWCCDEFQHPWSWY

>Thhalv10016913m Thellungiella halophila MPSEIVVRKRKSRDGTTVAETLQKWRDYNEQTKAASCNDDGGLKSLRKAPAKGSKKGCMKGKGGPENGICDYRGVRQRTWGKWVAEIREPGRGSRLWLGTFPSSYEAALAYDEAARAMYGQAARLNLPDITDGSSLIAPTVSSSITTLSDESEVCALEDTHVRSGFGHVKLEDGGDELVPLKSSMCVKEEVEVKEEMRELNSADASGIELAPKEETLDEWLVGIGNEQEPWDFGVDEVFDVDELLGLLSEIDVSGQETAQGQVDRESNLTYQMQFPDANLLGSLDHTETAHHFVQPSETENTGMDLDPPRFDDLDIEDIDFEGGEKDDQGGK

>Thhalv10017614m Thellungiella halophila MDPLPSQKHHLDDDNLHQQTLTNNPQSSDSTTTTTDSTSSAQQRKRKGKGGPDNSKFRYRGVRQRSWGKWVAEIREPRKRTRKWLGTFATAEDAARAYDRAAVFLYGSRAQLNLSPSSPTSVSSTSSSSVSASSTSPSSSSSSTQTLRPLLPRPSASGASAFGPYGLPLTNNNIFLSGGPSMLCPSYGLFSHQQQQQQQQSQMTQVGQFHQQQFQNLQPSNNSNKIGDVELTDVPVVNSTSFHQEVALGQEQRGCNNKMEDLNSLAGSVGSSLSISTAVVDPVGSMGMDTIGDVSSTVWPFGGEDEYSHWGNIWDFVDPFLLDF

>Thhalv10017658m Thellungiella halophila MEKEDNGSKQSSSSRRRRRAVEPVEATLKRWVKDEDEEGLEKVRRVQAKGSRKGCMRGKGGPENPVCRFRGVRQRVWGKWVAEIREPVSHRDVNSSRSKRLWLGTFDTAAEAALAYDRAASAMYGRYARLNFPEDLGNGPGREDMKKNDETESSTSYYGNAMIGMKDQKNDNGIEFGQDKTENLDPLVYENSAVKSEGDYSFDQFELDNGLLYNESSYYQGAGFDSYLEYSRF

>Thhalv10019142m Thellungiella halophila MSSIDPKVMMVGANKKQRTVQASSRKGCMRGKGGPENASCTYKGVRQRTWGKWVAEIREPNRGARLWLGTFDTSREAALAYDSAARKLYGPEAHLNLPESKRSYPDTASSGNTPSSNTGGKSGDSVNEESPCSSNEMMSSWGTEEISWPEHMNVDLPVTDDSSIWEEATMSLGFPWVHEGDDDISRFNTCISGGYSNWDSFHSPL

>Thhalv10021161m Thellungiella halophila MAVYEQTGIDTSKKRKPRARADGTTVADRLKKWREYNETVDASSIEEGEKPRRKVPAKGSKKGCMKGKGGPDNSHCSFRGVRQRVWGKWVAEIREPNRGSRLWLGTFPTAEEAASAYDEAAKAMYGTLARLNFPECVGSEFTSTSSHSEVCTVEDKAVLGGEVCVKQEDADCESKPFSQTLDVKEESSETSRLADEHRDANRMLNYDWLNEFEQQYLKEKEKPKEEDKEVIQQLEKQETDLLSVADYGWLNDMEKEQGFWNSHEFFDVDELLGDMNEGMLPDPSPNQDQNRIYDSYPLQLEPHDGHEFFDLSSLDL

>Tp3g09350 Thellungiella parvula MTVYEQTGADTTSKKRKSKARADGTTVADRLKKWKEYNKIVEASSIKEGEKPRRKVPAKGSKKGCMKGKGGPENSHCSFRGVRQRIWGKWVAEIREPNRGSRLWLGTFPTAEEAASAYDEAAKAMYGPLARLNFPQCVGFDIPSTSNQSEVCTVEDKAVLPGDVCVKQEDADSESRPVSQILDVKEGYSDIRWDNFAGERRDANSRLNSDLLNEFDEDYWSRGPREKEKPKEEDEKVIQPQPEPGMLTVSDYGWPYNMDIEPGLWDPNEVFDVDELLGDMDEGMITGPGPWQGQNQVHPGGYDSQPLQLEPHDGHEFFDLSSLDL

>Tp4g20670 Thellungiella parvula MQKEDNGSRQSSSASVESSRRRRRPAEPVEATLSRWEKEEGLEKVRRVQAKGSKKGCMRGKGGPENPVCRFRGVRQRVWGKWVAEIREPVNHRGGNSNRSKRLWLGTFDTAAEAALAYDKAASAMYGRYARLNFPKELGDVSGGEMKKNDEPECSRSYWLETNNVSETSNGIIKRKDEKDHLVYDYTIELGQEETENLDPMTDNEIVKSMVYPAVKTEEDYTFDRFELDNGLLYNESGYYHGGGFDPYSEYFGF

>Tp4g22680 Thellungiella parvula MPSKIVERKRKSRDGATSVTEILKKWREYNEQTEPASCNDDGGLKPIQKAHPKGSKKGCMKGKGGPENGICNYRGVRQRTWGKWVAEIREPGRGSRLWLGTFPGAYEAALAYDEAAKAMYGQSARLNLPDISNGSSSTAATVSGSITTLSDESEVCALEDMNLRSGLGQVKLEDGSDEHVPLNTSVCIKDELKVKYEMRVPLKSSQCVKEELDVKEEVRELNSADTFGIGLDPRKETLDEWLIGNGNEQEPWDFGVDEVFDANELLGLLGEIDVSGQETMQGQVDRPSNVTYQMQFPDANLLGSLNHTEIAHPGVDFGYPIVQPSEMENNCLDLERCKFQDLDIKDMGFEGQEKDVHG

>Tp5g05010 Thellungiella parvula MEKSSSMKQWKKGPARGKGGPQNALCRYRGVRQRTWGKWVAEIREPKKRARLWLGSFATAEEAAMAYDEAALKLYGHDAYLNLPHLQRKQGSHLSNIQRFKWVPSRKFISMFPSRGMLNVNAQPSVHVIQQRLEELKKNGLLSQSNSSSSSSTESKTKTSLLDERTSKGAIDKMLEGDDNDQKKPEIDLNEFLQQMGILKDKNQAEASEVAECHSTPPWNEQEETGSPFTAQSVSWDTLIEMPGIETSAMMFDSSNFGSYDFEDDLGFSSIWNFCGSLDE

>Tp5g30620 Thellungiella parvula MMVGANKKQRTVQASSRKGCMRGKGGPENASCTYKGVRQRTWGKWVAEIREPNRGARLWLGTFDTSREAALAYDSAARKLYGPEAHLNLPESLRTKPETASPGTHVSQTPSSNTGGKSSDSVNDSSPCSSNEMSSWGTRQEISWEHMNVDLPITDDSSIWEEATMSLGFPWVHEGDDDISPFNTCISGGYTNWDSFHSPL

>Tp6g25870 Thellungiella parvula MAEEQPPAKKRNMGRSRKGCMKGKGGPENATCTYRGVRQRTWGKWVAEIREPNRGTRLWLGTFNTSVEAAMAYDEAAKKLYGHEAKLNLLHPQQQQQQVNRNLSFSGHGSGSWPYRFDTVRGLDLGLGPSNGSRSSWSGSFSFLQEDDHHNSDRCLSSSGSNLSWLLPKRSNSQDQESFDAASVCGSEGGCITFSTKLKPNNLMTAPDYGSSNGVWSRFLVGQEKNDVSSSCGSSDNKESISVPSGRGDGEEGMHRPEMEEGTGYLEMDELLEIDDLGLLIGKNGDFKNWCCDEFQHPWNWF

>Tp6g37420 Thellungiella parvula MAVYDHSEDINRTQLDTSRKRKSRSRRDGITVAERLQIWKEYNGTVEEASTKKRKVPAKGSKKGCMKGKGGPDNSLCSFRGVRQRVWGKWVAEIREPNRGSRLWLGTFPTAEGAAAAYDEAAKAMYGPLARLNFPQCSVSDVASTSSQSEVCTAETPGVVHVKTEDADCESEPFFGEARPMYCMDNCEEMKKDVEVDVNASLPRNDWLSEFEQKYWSEVLEEKEKQKKQEIVVEPSQKRSDSLSVSDYGWPEDLDQCQWDSSEMFDVSELLGDLNGDIYTGLNQNQYAEDNVGGGLSEAEKQKIGLHPLQSFDSDYELPPLQLEAQDGNEFFDLSFLDL

>Thecc1EG021596t1 Theobroma cacao MIDKAAVISTTKSSKKKMVFNTTSRIRIWSTALKTEPCKRKTILPRQENDGIYVGRGLATSHRSAYDPYKRRHGINVGYFSYPPDGVVTHINTQNKRKLKGTTCKATGCLRLFMSMSSPMIRNSGSSVMEDGVRARNKKPRRRRNGRESVEDTIEKWKKYNNDQLQLGEEVGLKKVGKVPAKGSKKGCMQGKGGPENSRCKYRGVRQRIWGKWVAEIRQPINGVRVGNKGNNRLWLGTFSNAIEAALAYDKAAKAMYGPYARLNFPDHSEESAVHNSNNESASSTNETCSTESTSISNSFAEKAKESSVHYSSPVEEPELHVVEESKVCLVDKPMEKRDCSQVYINEEAGCIAEDTATETRGTEYNSRNDCKPYKEQGVEVETLKEAMDHELTELMRLHNDTNDYLHNELKDEGCQLSINFLDSDDYNLQTPFKKKEMESEVELSQNMLSSAYSGFNFRPNYMDNEEQDAGISIIDLEPSNDVKVEMPVTRENWKCELAGSIESIDYNIFSGKDDNLQTELTGGNLSLGFCYKPSSEMKAEAPVLMEEVEVEHGGFTDFNSYKGFGKTYDHMPYEPTDMICERQMNATTPTDFKAQTPINYGGFNSFKDKLDFLHSWPAEAITDVKPFALIRNDNCGLRPKESYNSDQFESSSTSYPERGGLQDPIAEAQGGFNQYETGFGEDYKLEFSRPDVDLDLGTDLWFPEHGF

>Thecc1EG024756t1 Theobroma cacao MESYRRSPLKPWKKGPTRGKGGPQNASCQYRGVRQRTWGKWVAEIREPKKRTRLWLGSFATAEEAAMAYDEAARRLYGPEAYLNLPHLQPNSNPPNKLQKFKWIPSKNFVSMFPSCGLLNINAQPSVHVIHQRLQELKKNGVLNQTGSSSSSSCESKTDIQIINDKTPAEDPQMKEKDVEISSDKMAGAYEDKPQIDLHEFLQQLGILREEKQSEGAETTESLTATDSSIKDYDEVAVFAENSFNWDAMIEMHGVADYQGAEASFQVHDAQEDLTFPASIWNF

>Thecc1EG026121t1 Theobroma cacao MEAEAKANCTKKVVISKRPSSLGKSRKGCKRGKGGPENAMCTYRGVRQRTWGKWVAEIREPNRGNRLWIGTFSTSLDAVLAYDEVARKLYGPSAKLNLPQPRDFPSITSFPGNLVNSCKETGMLGSPAVGESSGSSGSSIQSEERLVRRKISTEGFKGSVFLGNDGEEDFYWPEFSLENDFLKMNDIEVLMGQEFKGNWNGNEIAGIQSQWFF

>Thecc1EG034132t1 Theobroma cacao MSTSLMGGFGERKQIKKPAQASSRKGCMRGKGGPENALCTYKGVRQRTWGKWVAEIREPNRGARLWLGTFDTSHEAATAYDAAARKLYGLDAKLNLPELCVNPQYPPSSANTPIAPIGNQTQNVNNSGTSSSNSQIIMANDVKPVYNNDSVMSFPNENIGSQGNLAENNAKFGQNEDGIDGFWENMSVNLPVLDDSIWAEAAMSLDFPVMDNPGSFTGTFVDATGWDALQSPWCM

>Thecc1EG042471t1 Theobroma cacao MDQDQDQSPLSHPPQETTAKTTTTTTTSPDQDTKSSESESVIISPNTNPPGSSCTTTTSRKCKGKGGPDNNKFRYRGVRQRSWGKWVAEIREPRKRTRKWLGTFATAEDAARAYDRAAIILYGSKAQLNLQPSVSSSSSSSASSSRGGSSSSSSTQTLRPLLPRPSGFAFSFASSSNPVTHPASLIAGGSSSKFMPYGVYPNLLGPAALYPNIVQNPQQALQIVQQTEPSLVVNPGDPTGTLTSYTNPNPQQTQQHHQGSLYEDINSLVGSVGSSLSLSAQTSVAPAVPDPGLTVGPGSPSVWPLTNDDDYPPACIWDYGDPNFFFDF

>Thecc1EG043275t1 Theobroma cacao MVFVGDQNPKVGSTLSMDSSRKRKRRNGLSVADTLKLWSQNEEAKHPRKAPAKGSKKGCMRGKGGPQNQSCNYRGVRQRTWGKWVAEIRAPNRGKRLWLGTFPTAYEAALAYDEAARMMYGENAILNMPYVSDSDSVATTSNAFSEADFMTVWPKSEICGYHAFGEGERMNVEFPVDSEAPSTSGVINTTDEFQPAKPEEECMKETDYSWLNGLDFSEDIPIIGAPGVWDVGSYFSEDEVFNIDEILG

>Thecc1EG043302t1 Theobroma cacao MSFISNSKQGFAIGNGLTDPAIQNKAYLDYALDMGVIKKSDYNPINKLVPVCETAIKLCAGVDILYIVMDIQCNEILVVQLIKEIKVIFGDVASMGRGFQIPELLFTTLDHDCKKDQNLKVASTLSSDSCRKRKRRDGLSVADTLKLWSQSKDAKQSCKAPAKGSKKGCMKGKGGPQNQSCNYRGVRQRTWGKWVAEIRAPNGGKRIWLGTFPTACEAALAYDEAARTMYGENAILNMPHISGSDSVATTSHAFSEATTCAASNSMTVPSNYEICGRDVDREGEPSRMNVEYTVDSETAWTMDGENAMLNMPHVSDFDSVTITSPVFLEATTCTASNSMTGSSNSEICGRDVDGEGEPSKMNVEYTVDSEAATTSQDVKTDAKSEEHTDDSWLTNGLDYAKNIAIDFGETADWFEDYCFNVPEFFD

>Thecc1EG043306t1 Theobroma cacao MDQNLKVASTLSSDSCRKRKRRDGLSVADTLKLWSQGKDAKQSCKAPAKGSKKGCMKGKGGPQNQRCNYRGVRQRTWGKWVAEIRAPNGGKRIWLGTFPTACEAALAYDEAARTMYGENAIINMPHISRFDSVATTSHAFSEASTCSASNSMTVPSNYEICGRDVDGEGEPSRMNVEYTVDSETAWTMHGENATLNMPHVSDFDSVSITSPVFSEATTCSASNSMTVPINYEICGRDVDGEGEPSGMNVEYTVDSETAWTMDGENATLNMPHVADFDSVTITSPVFSEATTCAPSNSVTVSSNSEICERDVDGEGEPSKMNIEYTADSEAATTSQDVKTDAKSEDHTDDSWLTNGLDFAKNIAIDFGETADWFEDYYFDVPEFFD

>Scf00016.g2353.t1 Utricularia gibba MSTEIARRDLNLTHQPMDFLKMRKSRGGKVGTNSIAETLAKWREYNSKLDTVDKDNKKVYKAPAKGSKKGCMRGKGGPENARCNYRGVRQRTWGKWVAEIREPHRGSRLWLGTFNTGHEAAVAYDEAARAMYGPGARLNFPCCSSSKDSCSLPTTSDSGNSEVLLGDRGPDTGVSEIKKEADEACPENMDYRHPSVGNETISICKQQGIDETPAKIRMEDEAAAEESVSTQSTMYIHDPSALSGQQQLDEMFDVDDLLAALDSAPHLTRRPQVSLATSFPQHADRMPTESLSLPNQQPPYDYGLDFLKPGRQEDYNTMFLNLFMDFDKDMML

>Scf00044.g4995.t1 Utricularia gibba MLNSVEDKRIRKLAQASSRKGCMRGKGGPENALCTYKGVRQRTWGKWVAEIREPNRGSRVWLGTFDTAYDAAVAYDAAARKLYGPEAKVNLPELYEDGGAVYVNPDQASVNHGGGGGDSPVFSFSPEEMMSVQDSLSSSSGGGSGEYVNCSGILRSLNMSLPEVDDSYLWAEAAKGTSFGIVHDPGAFASSLDCFKDNNGVTFPWLY

>Scf00209.g12627.t1 Utricularia gibba MDNGRKLPFKPWKKGPARGKGGPQNAMCEYRGVRQRTWGKWVAEIREPQKRTRLWLGSFATAEEAAMAYDEAARRLYGPEAYLNLPHLRANFNPLNKSQKFKWFPAKDINDFPSTGLLNLNAQPSVHVIHQRLQELKKTGVIGQNSSTWSLSNKPKEVLHDTIEHPQTESYNEQNKDAETSSGNKEIASKEKPQIDLNEFLQQIGVLKREGQLPASEIPKILENTKSSTKDSVEELGNVIEKDINWETLSATVGGSIMSLGAEESSFQSHDGSGELVFPSSIWDFHENQDIFKGFDLPYIE

>Scf00215.g12838.t1 Utricularia gibba MRRRVLSFILSVSSHGKEGERQLGNKRMIDKERKKKLEFNQAVDENDERRTSFTTVWGMNLTHQAMELAKMRKSRGRKSGTANIAETLAKWKEYNTRNDVVDSDDKKVYKAPAKGSKKGCMRGKGGPENARCNYRGVRQRTWGKWVAEIREPHRGSRLWLGTFSTGHEAALAYDEAARAMYGPGARLNFPCCSSSMDSCSTGTTSDSMKSEGLLGERGHSADVSVIKKEVNEGCSQNVDSRNPSAVNETATHQVLDEKPIKDTKEEVAEESVPTQSTMYLHNGQHPRTLSGQHPSDVMFDVDDLLAALESAPPHPTHRPPEASFQEPLDAIPTESLSLPNLHDNGLDFLKPGRQEDYNTMFLDLFMDLDKDMVL

>GSVIVT01009801001 Vitis vinifera MRGKGGPENAMCTYKGVRQRTWGKWVAEIREPNRGARLWLGTFDTSYEAAIAYDAAARKLYGPEAKLNLPELCAQPPANTQFTHNQIPPNPNITPPSLTPLPRPNIQTPPYHKYSVVPSPATQEVPPHAKPTAINEKNGENDDGMEGVWANMNVTLPKVDDSLWTETLMSMDFPAFDDTEIFGSNLVDGNSWEMLQSPWRT

>GSVIVT01032746001 Vitis vinifera MSSGVIERKRKSRSRRSGPNSVAETLARWKQYNDILDSVRKAPAKGSKKGCMKGKGGPENSICGYRGVRQRTWGKWVAEIREPNRGSRLWLGTFPTAIEAALAYDEAARAMYGSSARLNLPNYTTSLKDSSSAPTTSVSDSTTTTSNYSEVCAYEDSKKPVLPSIKHEKNGHSLDAMYFKNEDGGQDFLEGFPMDEMFDVDEFLRAIDSDPLASYGTRQELGHDSGQVGSFETDNMQWEKPTDLSYQLQNPDAKLLGSLNHMEQVPSDFDYCYGFLQPGKQLDPSSPESGFTLAVFSVLQVYHLIPFAAVNIEEIELTLETICGRGIYVKTSIHFMHTRNLCRNQHPFHAHPFEAFYFGSWQAIELMRIEDGTVALHLAESEYVIEEKSPIPNLRIRPRKATLSDCTCFLRPGTEITVLWTLQQSESSDEENREPVWIDAKISSIERRPHEPECSCQFFVNFYITQDPLGTEKGTLSKDISVVELDQISILQKLGKYPCEDEHYRWKFSEDCSLLQRTKLFLGKFSSDLSWLVVTSVLKQAVFDVRSVQNRIVYQIVGGDHDKVSLNAVNFRVDNGISTPVIFPFVPADTIEADPLNGTNEAGPLPFCDIVDLRRSKRRNVQPDRFFSLGGFSESDIGSVRAGIHKVDYWRKEEMPLALPDEGDVHSIFSEKHIIDYEKGAHSLQIDSYEDFLVCKSKDRSREVKPILAAQNEDQHQFAIVPVPLIIEPIAHGEDHLHDETPWNESGEIGEISPKYYCTNGVPKLQRKNMSDLYMEVESRWEGKGPIRKLRRKRGFTIRTKTESYGEVRPHKKRPFSEPGYKEVIEAYMKNIESTINKEQPLVIDQWKELQVRNDLNQRRDCNSPSSVGDQEESSETEMLWREMEFSIASSYLLEENEGSNVEVLKEVVQESSNISEQVCQHEYILDEEIGVLCQLCGFVSTEIKDVSPPFFQPTGWITNREWRDEENSKRKQAENDGFNLFSIPASSDTPLSEGNDNVWALVPDLRKKLRLHQKKAFEFLWKNIAGSMVPALMEQEVKRRGGCVISHSPGAGKTFLVISFLVSYLKLFPGKRPLVLAPKTTLYTWYKEIIKWKVPVPVYQIHGCRTYRYEIYKHKVETSPGIPRPNQDVMHVLDCLEKIQKWHAHPSILLMGYTSFLSLMREDSKFIHRRYMGEVLRQSPGILVLDEGHNPRSTGSRLRKALMKVKTNLRILLSGTLFQNNFSEYFNTLCLARPKFVNEVLRELDPKFKRNKNRRKRRYSSTESRARKFFTDEIAKRINSNVPEEQIEGLNMLRNLTSKFIDVYEGGSSDNLPGLQVYTLLMKSTTIQQQFLSKLQKKKDEYKGYPLELELLVTLGSIHPWLITTAACADKYFSREELLELKKHKDDVKKGSKVKFVLSLVNRCIIRKEKILIFCHNISPINLFVDIFDKLYKWKKGEDVLVLQGDLELFERGRVMDQFEEPGGASKVLLASITACAEGISLTAASRVILLDTEWNPSKQKQAVARAFRPGQERVVYVYQLLETDTLEEEKNSRTNWKEWVSSMIFSEAFVEDPSCWQAEKIEDDLLREIVEEDWAKSIHMIMKNEKASNGLIRDVVKEMSLQNIQHIMVIMCVYCIISMYNMHNFVCIQNSLV

>GRMZM2G006745 P02 Zea mays MTLDQNHAMPMQPPALQPGRAYGAEGSAVVHGSIRTVAGGPTLALNECQILVQQKPQGDSRLLASNLWKKRPRRSRDGPTSVAAVIQRWAERNKHLEYEESEEAKRPRKAPAKGSKKGCMKGKGGPDNTQCGYRGVRQRTWGKWVAEIREPNRVDRLWLGTFPTAEDAARAYDEAARAMYGDLARTNFPGQDATTSAQAALSSTSAQAAPTAVEALQTGTSCESTTTSNHSDIASTSHKLEASDISSYLKEKCPAGSCGIQDGTPIVADKEVFGPLEPITNLPDGGDGFDIGEMLRMMESDPHNAGGADAGMGQPWYLDELDSSVLESMLQPEPEPEPEPFLMSEEPDMFLAGFESAGFVEGLERLN

>GRMZM2G028386 P01 Zea mays MDRVPPPVSMQVAAMQRHQQQQQFVHHLQQVHQQGTQHEQPPPPHQNGSSSSGRTGGGRKCCPLRRSRKGCMKGKGGPDNQQCPFRGVRQRTWGKWVAEIREPNRGARLWLGTFGSALEAARAYDAAARTLYGDCARLNLQLVPPSAAAAAAGGGGPAVVASPSPDTVAGPAAAAGGGGHNCHHQYLQQQHAMAAPMMMMHSSCCSADGSSSNSDSISNSCSSPVTTAASPAYSHHQTMFQTPALQPSCGAMTMAAAAPHVQGFHVGDDDTTTAMAMHRHQQMMRELAEAPLHQEADDFEDFVTRLPKAEDFGLQGFQEVAPEVFDDAAGIWDHAAAWEPPTMMIDSGAQPQQQLVVPL

>GRMZM2G156737 P01 Zea mays MDRLAPPVAVEVPVVHRQQQQQFVHHSQVHQQQRTHQQQQHQNGGGSKPGGRKCCPLRRSRKGCMKGKGGPDNQQCPFRGVRQRTWGKWVAEIREPNRGARLWLGTFGSALEAARAYDAAARALYGDCARLNLQLPPPSAAAGGGPAIVASPSPDTVAGPAAAAAGGHSRHHQYLQQQQPSMAAPMMMVHSSCCSADASSSNSDSNSNSSSSPVTMAAAATSPTYTHHQTTFQMPPPPPSSCGVMMAAVQGCHVDDTTTTTMAMQRHQQMLRELAAAPLHQEADDFEDFMTRLPKAEDFGLQGFQEVPPEVFDEAAGIWDHTAAWSSPTMMIDSAGAQRQQVVDSSSLFNSR

>GRMZM2G376255 P01 Zea mays MESYGHKRAWKKEPTRGKGGPQNAACEYRGVRQRTWGKWVAEIREPNKRARLWLGSFATAEEAALAYDEAARRLYGTDVFLNLPHLRASGVSAAGAGNAHERLRWLPAASARGAAVPAYGLLNLNAQHNVHVIHQRLQELKNNGSPADHLPAAASTSPCSTVTTRAAAALPAPMSCFHALEQAVTTVATRTTVDDAEPCEGGACPSGADKPQIDLSEFLQQIGVLNNTDDDGIRTTAKASFRGDAAEAGCFGGNGEFDWDALAADLNDIAGAGVHGGVNGGFQMDDLHEHEVEQFGTCLPIPVWDVQ

>GRMZM2G399098 P01 Zea mays MQSYGRKRAWKKGPTRGKGGPQNAACEYRGVRQRTWGKWVAEIREPNKRTRLWLGSFATAEEAALAYDEAARRLYGPDAFLNLPHLRASAVSSAAAHQRLRWLSASARGAAAVPAYGLLNLNAQHNVRVIHQRLQELKNNGSPAKPPPSPSLRVSPSAPHKQLAPADHLPAVATTSPCSIVVVTHVHAAPAMSCFHALEQTVATVAMRTVDDAEPCEGGACPPAADKPQIDLREFLQQIGVLNTDDDGITATAKAAFHAAGDAAVSCFVSGNGDFDWDVLAADLNDIAGAHGGANGGVQMDDLHEHAVDQFGTCLPIPVWDV

>GRMZM2G419901 P01 Zea mays MESYGRKRAWKKGPTRGKGGPQNAACEYRGVRQRTWGKWVAEIREPNKRTRLWLGSFATAEEAALAYDEAARRLYGPDAFLNLPHLRVSAAAAHQRIRWLPASARGAAAAAVPAYGLLNLNAQHNVHVIHQRLQELKNNGSPAKPPPPAPRVSPAARQQQLAPADHLPVASTSPCSTVTTHAAAAALPPPMSCFHALDLDLEQAVATAAMTTVDDAEPFEGGGAFPPGADKPQLDLREFLQQIGVLKTDDDGTATAKGSLHGDAADAGCFGGNGEFDWDALAADLNDIAGAHGGANGGFQMDDLHEVDQFGTCLPIPVWDV

**Supplementary Table S2 The sequences of all the primers and probes used in this study**

| primers | sequences of primers | usage |
| --- | --- | --- |
| LcDREB2-F | AGCGGCCCCTTCTGCGCCCTTC | semi-quantitive PCR |
| LcDREB2-R | TCCAGTTGTTGGTTCACTTCTTT |  |
| LcDREB2a-F | CTTCGCAAGACCCTTCCTCTA | real-time PCR |
| LcDREB2a-R | GTCGCTTTTTCCTTCCAGGCT |  |
| LcDREB2b-F | CTTCGCAAGACCCTTCCTCTA |  |
| LcDREB2b-R | GTCGCTTTTTCCTATTGGTTCG |  |
| LcDREB2c-F | CGAACCAATAGGAAGCAGTGAT |  |
| LcDREB2c-R | TGGTACAGCCACAGCAGGAG |  |
|  |  |  |
| SR protein name | SR protein primers name | Sequences of primers |
| OsSRp32 | OsSRp32-F | CGGGATCCATGAGCAGGCGGTGGAGC |
|  | OsSRp32-R | CCCAAGCTTTTACTGCACAGCATATTCAATATG |
| OsSRp33b | OsSRp33b-F | GGAATTCATGAGTAGGCGCTGGAGCAGGAC |
|  | OsSRp33b-R | CCCAAGCTTTCATTCGGACTTGGCATCAG |
| OsSRp20 | OsSRp20-F | GGAATTCATGGCCATACGCAAGTTCATGTATG |
|  | OsSRp20-R | ACGCGTCGACTCACCGGAGAGGGAGAG |
| OsRSp29 | OsRSp29-F | GGAATTCATGAGGCCCGTGTTCGTGG |
|  | OsRSp29-R | ACGCGTCGACTCAATCATAAGCTCTTCTGTTG |
| OsRSp33 | OsRSp33-F | GGAATTCATGAGGCCAGTTTTCTGTGGAAAC |
|  | OsRSp33-R | CCCAAGCTTTCATTCTCTCTCGTACCTCGC |
| OsRSZ36 | OsRSZ36-F | GGAATTCATGCCGCGCTATGATGATCGTTATG |
|  | OsRSZ36-R | CCCAAGCTTTCAGGCTTCAGGAGACTCGC |
| OsRSZ37a | OsRSZ37a-F | GGAATTCATGCCTCGCTATGATGACCGTGATC |
|  | OsRSZ37a-R | CCCAAGCTTTCAAGGGGATGCGCTTCCT |
| OsRSZ39 | OsRSZ39-F | GGAATTCATGTCTCGGTATGTTGACCCTAATG |
|  | OsRSZ39-R | CCCAAGCTTTCAACGGTCGCGGGACAG |
| OsRSZp21a | OsRSZp21a-F | GGAATTCATGGCCCGCGTGTACGTCG |
|  | OsRSZp21a-R | CCCAAGCTTCTAACTTCTGCTGCGGCGATATCC |
| OsRSZp21b | OsRSZp21b-F | GGAATTCATGGCCCGCTTGTATGTTGGC |
|  | OsRSZp21b-R | CCCAAGCTTCTAGCTCCTGCTACGGCGGT |
| OsRSZp23 | OsRSZp23-F | GGAATTCATGGCTCGCGTGTACGTGG |
|  | OsRSZp23-R | CCCAAGCTTTTACGCGTTATTTGCGTAGGG |
| OsSC35b | OsSC35b-F | GGAATTCATGTCGCGCTTCGGCCGCT |
|  | OsSC35b-R | CCCAAGCTTTTACGTCGAAGGGCTACGAGAGCG |
| OsSR45-1 | OsSR45-1-F | GGAATTCATGAGAACAGCAAAAAAAAAAAAAAA |
|  | OsSR45-1-R | CCCAAGCTTTTAATTGCGGATAGGGGAAC |
| OsSCL25 | OsSCL25-F | GGAATTCATGGGGAGAGGCTACAGTTACAGC |
|  | OsSCL25-R | CCCAAGCTTTCATTCACTGACAGAGAGCG |
| OsSCL26 | OsSCL26-F | GGAATTCATGGGAAGAGGCTATGATTATGGTCC |
|  | OsSCL26-R | CCCAAGCTTTCACCGGCTAACAGAAAGAG |
| OsSCL30a | OsSCL30a-F | GGAATTCATGAGGAGGTATAGTCCACCTTATCG |
|  | OsSCL30a-R | CCCAAGCTTTTAGTCACTGCGGGCAGG |
| OsSCL30b | OsSCL30b-F | GGAATTCATGAGGAGGTACAGCCCACCATATC |
|  | OsSCL30b-R | CCCAAGCTTTCAGTCGCTGCGGGCAGG |
| OsSCL-1 | OsSCL-1-F | GGAATTCATGGCCGGGTACCGGAGCC |
|  | OsSCL-1-R | CCCAAGCTTTCAGCTTTTCCCATCAACTGGAG |
| OsSCL-2 | OsSCL-2-F | GGAATTCATGAGGAGCCGCAGCCCCAG |
|  | OsSCL-2-R | CCCAAGCTTCTAGTTGCTGCTGGCGGCG |
| OsSR45-2 | OsSR45-2-F | GGAATTCATGGCGAAGCCGCGCCGCG |
|  | OsSR45-2-R | CCCAAGCTTCTACCTGCGCCTCGGGGAAGGTG |
|  | | |
| RNA probes | RNA probes sequences | |
| E3-1 | TAGGCGTATTCGTATGTCCTTAT | |
| E3-2 | CTGTCGTATTTCTAGGCGTATGTCCTTAT | |
